# Necrotrophic lifestyle of *Rhizoctonia solani* AG3-PT during interaction with its host plant potato as revealed by transcriptome analysis

**DOI:** 10.1038/s41598-020-68728-2

**Published:** 2020-07-28

**Authors:** Rita Zrenner, Franziska Genzel, Bart Verwaaijen, Daniel Wibberg, Rita Grosch

**Affiliations:** 1 Leibniz Institute of Vegetable and Ornamental Crops Großbeeren (IGZ), 14979 Großbeeren, Germany; 2 Genome Research of Industrial Microorganisms, Center for Biotechnology (CeBiTec), Bielefeld University, Universitätsstr. 27, 33615 Bielefeld, Germany; 3 Present Address: Institute of Bio- and Geosciences IBG-2, Plant Sciences, Forschungszentrum Jülich GmbH, 52425 Jülich, Germany; 4 Present Address: Faculty of Biology/Computational Biology, Bielefeld University, 26 Universitätsstr. 27, 33615 Bielefeld, Germany

**Keywords:** Fungal pathogenesis, Transcriptomics

## Abstract

The soil-borne pathogen *Rhizoctonia solani* infects a broad range of plants worldwide and is responsible for significant crop losses. *Rhizoctonia solani* AG3-PT attacks germinating potato sprouts underground while molecular responses during interaction are unknown. To gain insights into processes induced in the fungus especially at early stage of interaction, transcriptional activity was compared between growth of mycelium in liquid culture and the growing fungus in interaction with potato sprouts using RNA-sequencing. Genes coding for enzymes with diverse hydrolase activities were strongly differentially expressed, however with remarkably dissimilar time response. While at 3 dpi, expression of genes coding for peptidases was predominantly induced, strongest induction was found for genes encoding hydrolases acting on cell wall components at 8 dpi. Several genes with unknown function were also differentially expressed, thus assuming putative roles as effectors to support host colonization. In summary, the presented analysis characterizes the necrotrophic lifestyle of *R. solani* AG3-PT during early interaction with its host.

## Introduction

The soil-borne fungus *Rhizoctonia solani* (J. G. Kühn) [teleomorph *Thanatephorus cucumeris* (A. B. Frank) Donk] belongs to the phylum *Basidiomycota*. *R. solani* is a soil-borne plant pathogenic fungus with worldwide distribution and infects many economically important crops like rice, soybean, potato, maize, sugar beet, cabbage, tomato, and lettuce^1^. *R. solani* is a species complex of various groups called anastomosis groups (AG), some of which are subdivided into additional subgroups^2,3^. Hyphae of isolates belonging to the same AG are able to anastomose. In total, 13 different AGs of *R. solani* have been described with their subgroups differing in morphological and genetic characteristics^4,5^. Members of the different AGs and the various subgroups show a distinct degree in host specificity^6,7^.

Diseases on potato caused by *R. solani* occur in all areas, where potatoes are grown and affect the qualitative and quantitative yield of potato tubers. About 30% of tuber yield loss has been reported^8^. *Rhizoctonia solani* AG3 was found to be the predominant AG associated with potato^9–13^, and the so-called *R. solani* AG3-potato type (PT) was developed^14^. Infection of potato by *R. solani* AG3-PT may result in the characteristic disease symptoms of stem canker and black scurf^15^. Shortly after planting, necroses on germinating sprouts are the typical symptoms of stem canker, and results in late emergence of potato plants in the field, lower numbers of stems, shorter stolons and deformation of progeny tubers^16^. It is postulated that necrotic lesions on the stolons disrupt the delivery of photosynthates leading to this characteristic tuber phenotype^17^. Black scurf symptoms appear later in the season, when sclerotia start to cover ripening potato tubers^8^. These tuber-borne *R. solani* inocula can be the main source of primary infection causing stem canker symptoms of below-ground plant tissue of the next crop. Increasing incidence of this plant disease and a current lack of effective control measures requires an improved understanding of the biology behind the processes.

Based on their feeding strategies, plant pathogenic fungi are divided into biotrophs, which feed on living host tissue and necrotrophs, which kill the host tissue and feed on the remainders^18^. So-called hemi-biotrophs use both strategies initially infecting the host biotrophically and then shifting to the necrotrophic stage^19^. Necrotrophic pathogens show aggressive and wide-ranging virulence strategies that result in host cell death^20^. To this end necrotrophs secrete proteinaceous, non-ribosomal peptides, metabolite toxins, and cell wall-degrading enzymes to induce host cell necrosis and leakage of nutrients^21^. These virulence mechanisms targeting diverse host cellular processes are countered by a complex host response involving cellular, histological, biochemical, and molecular answers. In early stages of necrotrophic interaction, plant host cell death is associated with production of various secondary metabolites, antimicrobial peptides, and hormones, accumulation of reactive oxygen species, callose, and cell wall modifications^21^. The soil-borne pathogen *R. solani* is considered to be a necrotrophic plant pathogen with broad host-range. However, recent studies on *R. solani* AG-1 IA causing sheath blight on rice and *Brachipodium distachyon* suggest a hemi-biotrophic nature of the *R. solani* isolate, since protective effect of the plant can be induced by treatment with salicylic acid^22^.

At present, there is only limiting information about the molecular responses of *R. solani* AG3 during the pathogenic interaction with its host plant. Histological observations of potato in response to *R. solani* infection were described^23^, and there is some information of phytotoxins (3-methylthiopropionic acid, 3-methylthioacrylic acid) produced by *R. solani* AG3 with relation to disease symptoms^24^. The expression of selected pathogenesis-related genes of the potato plants´ response upon infection has been analyzed^25^ and there is information of selected *R. solani* AG3 genes whose expression is correlated with the interaction process^26,27^. However, RNA sequencing technologies can give a deeper insight into the gene regulatory networks mediating disease outcomes. Dual sequencing of host–pathogen interactions provides a snapshot of the underlying transcriptional programs from both the host and the pathogen. For instance, such analysis showed that the important pathogen *Septoria tritici* on wheat alters gene activity to suppress plant defense response during the biotrophic phase before changing to a necrotrophic life style^28^. So far, RNAseq experiments are often limited to in vitro infection systems that do not fully reflect the conditions of the in vivo environment. For instance, a comprehensive transcriptome analysis of the soil-borne pathogen *R. solani* has been performed with the *R. solani* AG1-IB isolate 7/3/14 grown on root exudates^29^, during interaction with its host plant lettuce in an in vitro leaf model system^30,31^ and with *R. solani* AG1-IA during infection of soybean using a detached leaf assay^32^. A global transcriptome profiling with *R. solani* AG3-PT isolate Rhs1AP also carried out in vitro during interaction with plant associated bacteria (*Serratia plymuthica* AS13 and *S. proteamaculans* S4) revealed major shifts in gene expression with a simultaneous alteration of primary metabolic processes and activation of defense and attack mechanisms^33^.

In this study, we are focusing on transcriptome changes in *R. solani* AG3-PT during interaction with a medium resistant potato cultivar. The used isolate in this study, Ben3, originated from sclerotia on mature potato tubers and was characterized as *R. solani* AG3-PT. The draft genome of isolate Ben3 was analyzed and the size of the diploid genome was estimated to correspond to 116 Mb^34^. Gene prediction resulted in 12,567 identified genes, which is in the same range as for other completely sequenced AG3 isolates named Rhs1AP^35^ and RS-20^36^. With this global expression studies using RNAseq, we want to understand in its entirety the molecular interaction in the pathosystem *S. tuberosum* and *R. solani* AG3-PT*.* Together with the concomitant transcriptome analysis in the potato host this extensive study will finally broaden the basis for functional and comparative analyses of the host pathogen interaction of *R. solani*.

## Results and discussion

### Transcriptome analysis

The interspecific interaction between *R. solani* AG3-PT isolate Ben3 with the medium resistant potato cultivar 'Arkula' was analyzed on transcriptome level using RNA sequencing. In order to find factors important for the establishment of the interaction and further interaction progression, we used three different samplings: pure mycelium of *R. solani* AG3-PT isolate Ben3 cultured without being attracted by a growing potato plant (Ben3); potato sprouts at 3 dpi of the tubers with *R. solani* (early) and 8 dpi (late). At both sampling dates of *R. solani* in interaction with the potato sprout all emerging sprouts were harvested and used in the analysis. Necrotic lesions on sprouts become first visible at 8 dpi. Subsequently, RNA extraction, RNA sequencing (RNAseq) and mapping of the reads to the genome of isolate Ben3^34^ was performed to calculate reads per kilobase per million mapped reads (RPKM) values. In general, for the inoculated samples between 1 and 23% of each dataset was mapped on the Ben3 draft genome. For the control samples, between 70 and 75% of these datasets could be mapped on the Ben3 genome.

In all three samplings a comparable number of genes could be detected as being expressed (Table 1). In pure mycelium 11,206 genes out of the identified 12,567 genes on the *R. solani* AG3-PT isolate Ben3 genome were expressed, while reads could be mapped on 10,181 and 9,939 genes at 3 dpi and 8 dpi, respectively. In summary, in all transcriptomes within this experiment 11,287 genes out of the identified 12,567 genes on the *R. solani* AG3-PT isolate Ben3 genome were expressed in any of the samples analyzed.

**Table 1:** Summarized mapping statistics on the *R. solani* AG3-PT isolate Ben3 genome.

	Ben3 (pure mycelium)	early (3 dpi)	late (8 dpi)
Total mapped reads to the *R. solani* AG3-PT isolate Ben3 genome	108,263,968	4,016,186	2,651,869
Corresponding number of genes with expression	11,206	10,181	9,939

The values of the biological replicates are summarized in this table.

As obvious from Table 1, the amounts of total mapped reads differed considerably. This is owed to the fact that in the Ben3 sampling the transcriptome of the pure mycelium of *R. solani* AG3-PT isolate Ben3 has been sequenced, while in the early and late samplings (3 and 8 dpi) a dual RNAseq approach has been used to access the transcriptomes of both interacting organisms simultaneously^30^. Since library sizes and the amount of sequences produced from each library were comparable, in the Ben3 sampling almost all of the reads could be mapped to the isolate Ben3 genome. In the dual RNAseq approaches of the samplings at 3 and 8 dpi, only a fraction of the produced reads could be mapped to the Ben3 genome, while the others mainly mapped to the potato genome. Due to these different amounts of total mapped reads to the *R. solani* AG3-PT isolate Ben3 genome per sampling the following has to be considered: (1) genes with low RPKM value only in Ben3 are not necessarily not expressed when the fungus challenges the plant, it might be just owed to the library sizes; (2) genes that are found to be expressed in all three samplings could be assigned as being commonly expressed; (3) genes that are only found to be expressed in the much smaller libraries of the 3 and 8 dpi samplings can be assumed as being interaction specific.

**Figure 1:**
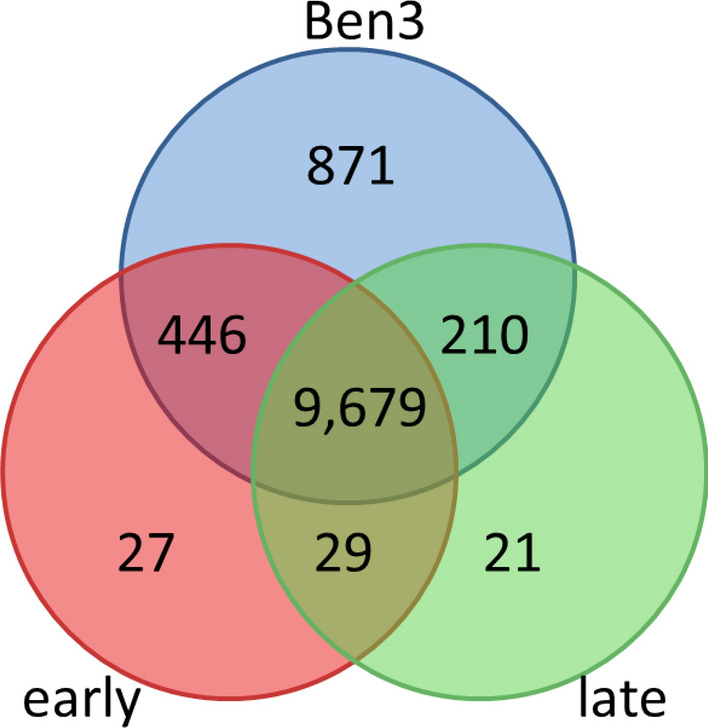
Venn diagram of transcribed genes in the three analyzed samplings. Pure mycelium of *R. solani* AG3-PT isolate Ben3 cultured without being attracted by a growing potato plant (Ben3); Ben3 in interaction with potato sprouts at 3 dpi (early); Ben3 in interaction with potato sprouts at 8 dpi (late).

The summarized mapping results (Table 1) already indicated that there must be many genes commonly expressed in all three samplings. Therefore, a Venn diagram was constructed to compare the three samplings and visualize the calculated number of genes they have in common versus the number of genes that distinguishes the individual samplings (Fig. 1). More than 9,000 genes were found to be expressed in all three samplings, while 871 were exclusively transcribed in the mycelium without plant contact. Expression of 29 genes was found to be in common in the 3 and 8 dpi samples, representing genes only expressed in the presence of the living potato plant. In addition, at 3 dpi 27 genes were exclusively expressed, and sequence reads mapping to a small set of 21 genes were exclusively detectable at 8 dpi. Lists of these genes together with their respective RPKM values and descriptions are given in Supplementary Tables 1 – 4.

From the 29 genes that were commonly expressed in the presence of the living potato plant, four genes are coding for proteins involved in plant cell wall degradation. This matches the virulence strategy of a necrotrophic pathogen with secretion of cell wall-degrading enzymes to induce host cell necrosis and leakage of nutrients^21^. In addition, one gene coding for a protein involved in protein degradation further supports this line of attack. Moreover, two genes coding for proteins with DNA-binding domains and putative function in transcriptional regulation are also in this list and are likely to play a role in transcriptional regulation of the offence (e.g. *Ben3g4553*, see Table 2). The majority of the 27 genes exclusively expressed at 3 dpi are coding for hypothetical proteins where no putative function can be assigned. From the 21 genes that were exclusively expressed in later stages of the interaction (8 dpi), eight genes coding for proteins involved in plant cell wall degradation and two are coding for proteases. This again demonstrates the typical strategy of a necrotrophic pathogen.

**Table 2:** Validation of DESeq2 analysis with qRT-PCR of 3 genes with different expression pattern.

SeqName	ΔCq (a)	ΔCq (b)	ΔΔCq	BaseMean	Log2fold change	Comparison (a):(b)
*Ben3g3530*	− 1.92 ± 0.59	6.38 ± 0.32	8.30*	451	8.93	(early):(Ben3)
	− 1.00 ± 0.92	6.38 ± 0.32	7.37*	181	7.79	(late):(Ben3)
	− 1.92 ± 0.59	− 1.00 ± 0.92	− 0.92	–	n.s	(early):(late)
*Ben3g2070*	− 4.17 ± 0.95	5.59 ± 0.53	9.76*	1,102	9.00	(early):(Ben3)
	0.47 ± 2.03	5.59 ± 0.53	5.12*	109	5.27	(late):(Ben3)
	− 4.17 ± 0.95	0.47 ± 2.03	− 4.64*	222	− 2.29	(early):(late)
*Ben3g4553*	− 5.83 ± 0.29	n.d	–	64	9.97	(early):(Ben3)
	n.d	n.d	–	–	n.s	(late):(Ben3)

ΔCq, relative expression value calculated as Cq_*gene*_ − Cq_*TUB2*_; ΔΔCq, differences in relative expression levels calculated as (ΔCq (b) − ΔCq (a)); *, stands for significant difference of ΔΔCq (n = 3; *P* ≤ 0.05; *t*-test); BaseMean, average calculated with DESeq2 package as implemented in the ReadXplorer software; Log2fold change, calculated with the DESeq2 package as implemented in the ReadXplorer software; Comparison, as indicated, pure mycelium of *R. solani* AG3-PT isolate Ben3 cultured without being attracted by a growing potato plant (Ben3) compared to Ben3 in interaction with potato sprouts at 3 dpi (early) or at 8 dpi (late); Values are given in Supplementary Tables 8–10; n.s., not significant; n.d., not detected.

In summary, a first examination of the transcriptome data of the three samplings revealed distinct and reasonable differences, thus demonstrating the potential of this study to find factors important for the establishment of the interaction between *R. solani* AG3-PT isolate Ben 3 with a susceptible potato cultivar and further interaction progression.

### The most abundant *R. solani* AG3-PT transcripts in the three samplings

The interspecific interaction between *R. solani* AG3-PT isolate Ben 3 with the medium resistant potato cultivar 'Arkula' was initially evaluated by examining the most abundant transcripts in the three different samplings. In growing mycelium cultivated in liquid culture, transcripts of 11,206 genes of the isolate Ben3 genome were found. In total, 698 transcripts of these genes were detected with median RPKM values of 100 and higher, 37 with median RPKM values greater than 1,000. At early interaction phase (3 dpi) of tuber sprouts with the pathogen*,* transcripts of 10,181 genes were detected with 729 showing median RPKM values of 100 and higher, 25 median RPKM values greater than 1,000. A number of 9,939 genes of *R. solani* AG3-PT were transcribed with 742 showing median RPKM values of 100 and higher, 26 median RPKM values greater than 1,000 at 8 dpi (Supplementary Tables 5–7). In total, 9,679 genes of the isolate Ben3 genome were found to be expressed in all three samplings, among them are the most abundant transcripts in each of the individual sampling analyzed. Five of these highest expressed genes (*Ben3g9573*, *Ben3g6448*, *Ben3g5323*, *Ben3g2326*, and *Ben3g675*) are coding for *R. solani* specific hypothetical proteins with unknown functions. Hence, these proteins were not expected to play specific roles during the interaction with the plant, these proteins may rather be important for general growth and cellular metabolism. Among the highest abundant transcripts in each of the individual sampling are also several proteins containing lectin domains (*Ben3g9146*, *Ben3g9350*, and *Ben3g8869*). A number of such proteins containing a ricin-type beta-trefoil lectin domain have also been reported previously as most abundant in the *R. solani* AG1-IB isolate 7/3/14 during interaction with its host plant lettuce^30^. While specific roles of these lectin domain proteins are undetermined it has been proposed that *R. solani* lectins could have a function as storage protein within the mycelium^37^. In addition, two genes coding for proteins with thuringiensis toxin domain (*Ben3g8806*, and *Ben3g11931*) were also strongly transcribed in all three samples analyzed. *Bacillus thuringiensis* toxins are bacterial proteins known for their biocidal activity against insects^38^, but a range of further organisms are targeted as well^39^. The strong expression of such toxin domain homologues in *R. solani* indicates that these toxins may be of general importance for *R. solani* AG3-PT isolate Ben3 rather than playing a specific role in the fungus plant interaction. A gene coding for a septal pore cap protein (*Ben3g7115*) was also among the most abundant transcripts in all three samplings, indicating its contribution to hyphal homeostasis in basidiomycetous fungi^40^. A transcript similar to the septal pore cap protein (RSOLAG1IB_6054) was also highly abundant in *R. solani* AG1-IB isolate 7/3/14 transcripts found in symptomless zone of interaction with lettuce^30^. This protein specific for *R. solani* is part of the plugging material that closes the perforations within the septal pore cap of hyphal cells and prevents transport of cytoplasmic fluids between neighboring cells. Furthermore, a gene encoding a hemopexin domain protein (*Ben3g6614*) was also among the highest expressed genes in common to all three samplings. This domain denotes zinc-dependent metalloproteinases, which are widely recognized to play an important role in the homeostatic regulation of the extracellular environment^41^, but their biological functions may also extend beyond extracellular matrix degradation^42^. Since all these proteins showed high abundance in *R. solani* AG3-PT with and without contact to the host plant, they may be important for overall growth and metabolism but do not seem to be of a specific relevance in the fungal interaction with the plant.

### The interaction between *R. solani* AG3-PT and potato

**Figure 2:**
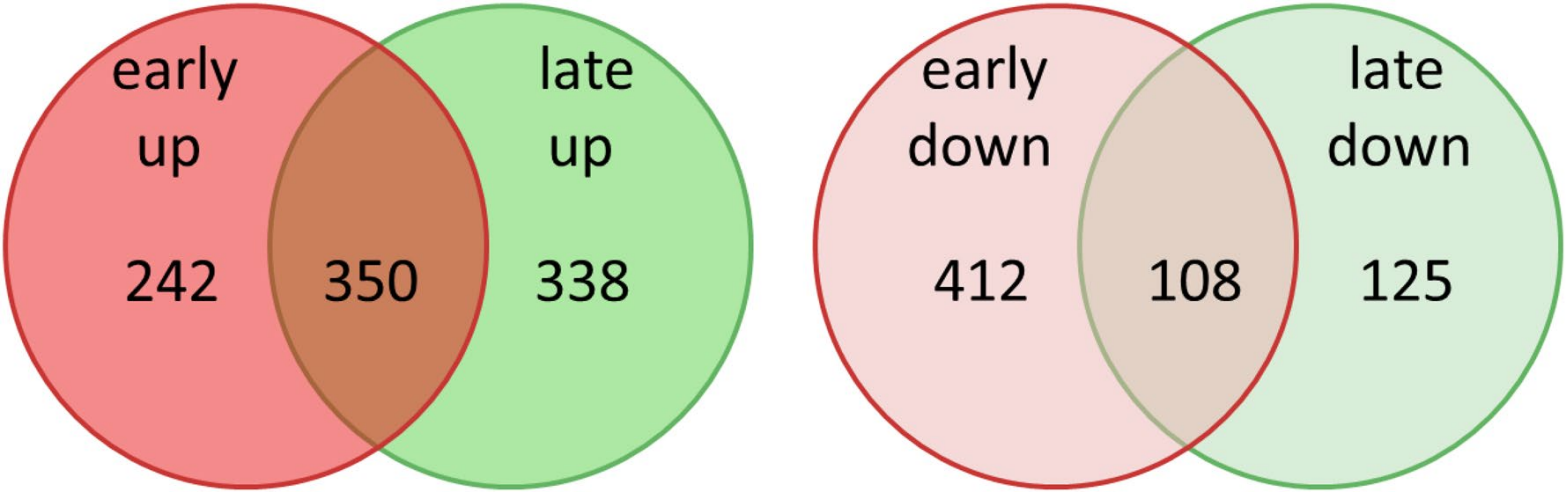
Venn diagrams of differentially expressed genes between the samplings. Pure mycelium of *R. solani* AG3-PT isolate Ben3 cultured without being attracted by a growing potato plant (Ben3) compared to Ben3 in interaction with potato sprouts at 3 dpi (early); pure mycelium of Ben3 compared to Ben3 in interaction with potato sprouts at 8 dpi (late).

In order to find elements with relevance for supporting interaction of *R. solani* AG3-PT isolate Ben3 with the potato sprout, differential gene expression was performed as integrated in the ReadXplorer platform (v2.2)^43^. This pairwise comparison of transcriptomes of pure mycelium of isolate Ben3 with transcriptomes of either 3 dpi or 8 dpi of interaction with potato sprouts was accomplished using the DESeq2 program. Genes were assigned as differentially expressed with an adjusted *P* value of less than 0.05 and a minimum fold change of |2| or more. Using these criteria 592 genes could be assigned as differentially induced at 3 dpi (sum of 242 genes exclusively early induced and 350 genes induced at 3 dpi and also at 8 dpi; early up) while 520 genes are differentially reduced at 3 dpi (sum of 412 genes exclusively early reduced and 108 genes reduced at 3 dpi and also at 8 dpi; early down). At 8 dpi, 688 transcripts were found to be differentially induced (sum of 338 genes exclusively late induced and 350 genes induced at 8 dpi and also at 3 dpi; late up) and 233 are differentially reduced (sum of 125 genes exclusively late reduced and 108 genes reduced at 8 dpi and also at 3 dpi; late down). Venn diagrams were constructed to compare and visualize the differentially upregulated as well as downregulated genes at both time points during the interaction (Fig. 2). Lists of these genes together with their respective values of fold change are given in Supplementary Tables 8–9.

Expression differences found with this DESeq2 analysis were validated in experiments using qRT-PCR. Therefore a suitable control gene with invariant expression pattern has to be established. Glyceraldehyde-3-phosphate dehydrogenase (*Ben3g7151*, *GAPDH*), ubiquitin-conjugating enzyme E2 (*EUC63156*, *UBC*), ubiquitin-protein ligase E3 (*Ben3g494*, *UBI*), elongation factor 2 (*Ben3g3364*, *EF-2*), and beta tubulin genes (*Ben3g4099*; *TUB1*) and (*Ben3g5288*, *TUB2*) were tested and the beta tubulin gene *Ben3g5288* (*TUB2*) showed to be the most appropriate reference in our experiments. Relative transcript levels of three candidate genes with different expression patterns were normalized on the basis of expression of this invariant control. ΔΔCq values were calculated and compared with the respective DESeq2 analysis (Table 2).

For all three candidate genes with very different expression pattern the calculated ΔΔCq values were in accordance with the respective log2fold change values computed in the DESeq2 analysis thus demonstrating the validation of expression differences.

In addition, gene ontology (GO) annotations were performed in order to assign respective functions to the particular genes. In the further analysis, a specific focus was laid on significantly induced genes to preferentially catch molecular candidates that might be important for initiation and establishing the interaction with the plant.

### Differentially expressed genes (DEGs) in isolate Ben3 at 3 dpi of potato sprouts

**Figure 3:**
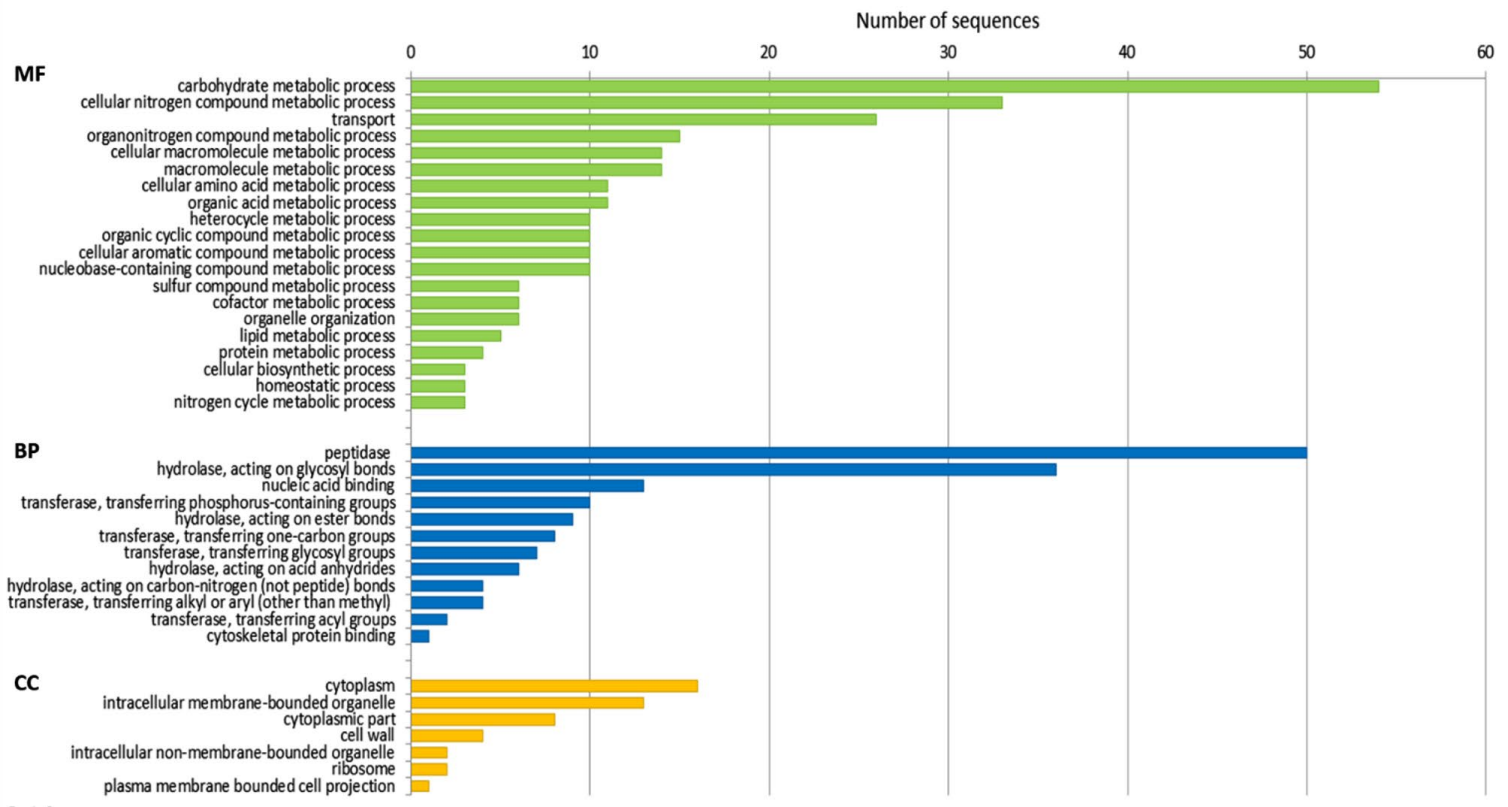
GO term distribution of differentially increased genes for biological process (BP), molecular function (MF), and cellular component (CC) at 3 dpi. Pure mycelium of *R. solani* AG3-PT isolate Ben3 cultured without being attracted by a growing potato plant (Ben3) compared to Ben3 in interaction with potato sprouts at 3 dpi (early).

Differentially induced genes in isolate Ben3 in interaction with potato sprouts at 3 dpi compared to pure mycelium of the isolate were selected assuming functions in initiating and supporting of the interaction process with the potato sprouts. Putative functions of the particular gene products and their distribution into the common GO aspects for biological process (BP), molecular function (MF), and cellular component (CC) are given in Fig. 3.

At 3 dpi, differentially induced genes are mainly assigned as being involved in carbohydrate and cellular nitrogen compound metabolic processes and in transport. Almost 10% (50 genes) of the differentially upregulated genes are encoding various peptidase activities (e.g. *Ben3g2070*, see Table 2), while 53 genes are encoding cell wall degrading enzymes including several different hydrolases acting on glycosyl bonds, and pectate lyases (e.g. *Ben3g3530*, see Table 2.) or xylanases. In order to further specify these cell wall degrading enzymes, all of the DEGs were also annotated according to the Carbohydrate Active enZyme (CAZy) database (Supplementary Table 11). Secretion of a large arsenal of hydrolytic enzymes such as proteases and cell wall degrading enzymes during the course of interaction^44,45^ is required for necrotrophic fungal plant pathogens like *R. solani* to induce cell necrosis by breakdown of structural protein and carbohydrate components of the plant cell walls and to cause leakage of nutrients^9,30,46,47^. Thus, it is hypothesized that the induced cell wall degrading enzymes and several of the induced peptidases function in supporting interaction of *R. solani* AG3-PT with tissue of the potato sprout. However, secreted peptidases have also been extensively studied for their roles as effectors of gram-negative bacteria^48^ and also fungi^47,49^. Pathogen effectors are usually delivered as virulence factors into the host cells to suppress the basal defense responses and create a suitable environment for pathogen propagation^50–52^. Thus, the post-translational modification of host proteins through proteolytic processing is a widely used mechanism in regulating the plant defense response. With the current stage of knowledge it might be supposed that one or more of these induced proteases have putative roles as effectors that support the interaction of *R. solani* AG3-PT on the potato host. However, further functional analyses of the individual proteases are needed to clearly assign their functional role in the pathogen host interaction.

Another big group composed of 100 members of differentially upregulated genes of the interaction at 3 dpi is described as coding for hypothetical proteins. Most of these genes are *R. solani* specific and homologues could be found in the other five annotated genomes of *R. solani* AG3 Rhs1AP, *R. solani* AG2-2IIIB, *R. solani* AG8, and *R. solani* AG1-IA and AG1-IB. From our differential gene expression analysis, it is expected that some of these genes are involved in supporting interaction of *R. solani* AG3-PT on the potato sprout. It is already known that secreted effectors of fungal pathogens target host immunity using various strategies e.g. by hydrolyzing a salicylic acid precursor^53^, or by binding to transcription factors thus inhibiting their activity^54^. Further analyses are needed to reveal putative functions of the so far hypothetical proteins for their various possible roles in the interaction of *R. solani* AG3-PT with potato.

Interestingly, the strongest differentially increased gene (*Ben3g6247*) is homologous to lipid-translocating exporter (LTE) family proteins, like RTA1 from *Saccharomyces*. The RTA1 protein contains seven potential membrane-spanning segments^55^ and is predicted an integral membrane protein with function in cell resistance to xenobiotics^56^. Other LTE family genes may encode transporters or sensors that facilitate the excretion of biosynthetic intermediates, either directly or indirectly^56^. These putative functions of the LTE family proteins make the gene *Ben3g6247* a favorite candidate involved in secretion of components important for the pathogen attack.

Early phase of necrotrophic interaction is associated with cell death of the plant host and production of various secondary metabolites and the accumulation of reactive oxygen species^21^. It has been shown that antioxidant processes and respective gene expression were correlated to necrotic tissues in several *R. solani* pathosystems (potato sprout-*R. solani* AG3; soybean hypocotyl-*R. solani* AG4 and soybean leaves-*R. solani* AG1-IA)^27^. At early phase of interaction of the potato host with isolate Ben3 (3 dpi) no strong evidence for induction of antioxidant processes in pathogen hyphae could be observed on transcriptional level, because there was no strong increase in glutathione S-transferase gene expression or upregulation of other genes known to be involved in the scavenging of reactive oxygen species. Therefore, it could be postulated that in our system to colonize the potato sprout with *R. solani* AG3-PT, tissue analysis at 3 dpi resembles an early stage of the plant pathogen interaction maybe prior infection of sprout tissue. No visible symptoms were observed at this time point.

### Differentially expressed genes in isolate Ben3 at 8 dpi of potato sprouts

**Figure 4:**
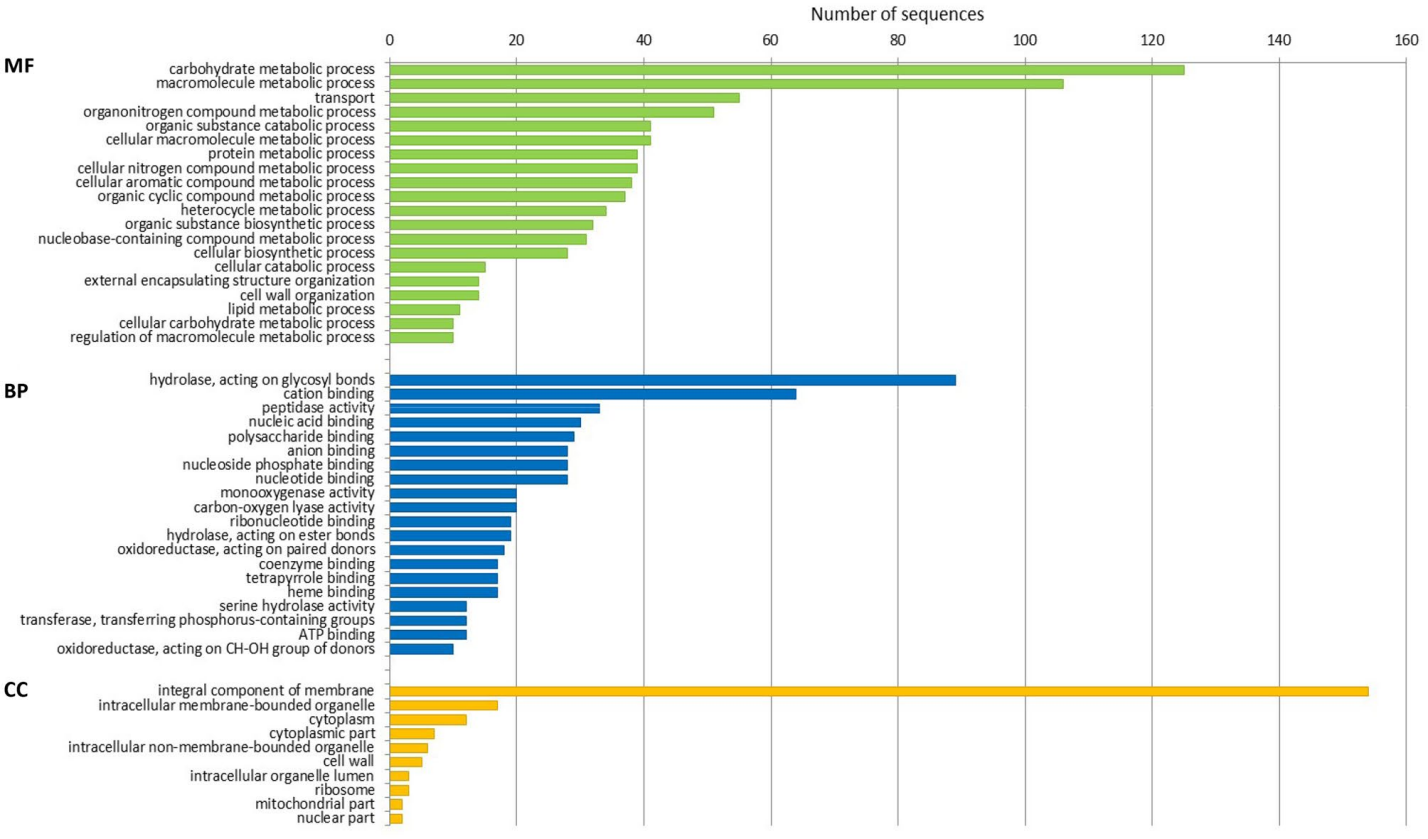
GO term distribution of differentially increased genes for biological process (BP), molecular function (MF), and cellular component (CC) at 8 dpi. Pure mycelium of *R. solani* AG3-PT isolate Ben3 cultured without being attracted by a growing potato plant (Ben3) compared to Ben3 in interaction with potato sprouts at 8 dpi (late).

In order to find transcripts that are important in an advanced stage of the interaction, differentially induced genes between pure mycelium of isolate Ben3 and Ben3 attracted to potato sprouts at 8 dpi were screened. Respective functional annotations of the gene products and their distribution into the common GO features are shown in Fig. 4. At 8 dpi, differentially induced genes are mainly assigned as being involved in carbohydrate and macromolecular metabolic processes and in transport. At this later stage of interaction, the upregulated 152 genes (> 22%) are encoding various cell wall degrading enzymes (e.g. *Ben3g3530*, see Table 2). In addition, all of the DEGs were also annotated according to the Carbohydrate Active enZyme (CAZy) database (Supplementary Table 11). This increased expression of genes coding for cell wall hydrolytic enzymes nicely demonstrates the increasing pathogenic activity of the isolate Ben3 as well as the importance of breakdown of cell wall components to access the nutrients during the course of interaction, thus confirming the described virulence strategy of a necrotrophic pathogen^21,57^. This destructive tactic was accompanied by an induction of expression of genes encoding integral components of membranes. These 154 mostly uncharacterized integral membrane proteins and putative transporters were presumably involved in the uptake of nutrients and degradation products of the hydrolase activities. At this stage of interaction the predominant importance of genes coding for peptidases seemed to be decreasing, but still 33 genes encoding peptidases were differentially upregulated (e.g. *Ben3g2070*, see Table 2). This could also be explained by the fact that whole sprouts were harvested, including the sprouts with up to 8 days of interaction with the challenging pathogen and the subsequent emerging sprouts with a shorter interaction period. In general, this is also represented in the 350 genes that were in common differentially increased at 3 and 8 dpi (Fig. 2).

In addition, another big group of DEGs at 8 dpi is composed of 98 genes with unknown function. Since these genes are Rhizoctonia specific without any regions of similarity to sequences with assigned functions, it can only be speculated about their role in supporting the pathogen plant interaction. Further functional comparisons of these genes and e.g. their differential expression in the respective pathosystems might give hints to their putative function.

In the here described experimental system to colonize the potato sprout with *R. solani* AG3-PT isolate Ben3 lesions become first visible at 8 dpi. It has been shown in other experiments^25,27^ that necrotrophic interaction and cell death in the plant host was correlated with respective gene expression in the plant and in the fungus. Samsatly and co-workers^27^ demonstrated by using quantitative RT-PCR, that the expression of antioxidant genes coding for glutathione S-transferase and catalase were significantly increased in *R. solani* AG3 five days after inoculation of detached potato sprouts. But such strongly increased expression of these antioxidant genes could not be found in the here described experiments. Reasons for these differences could be due to inoculation using isolates of *R. solani* AG3 exhibiting differences in pathogenicity. However, another distinction is the fact that Samsatly and co-workers^27^ performed experiments with detached sprouts in a limited in vitro system while our experimental setup fully reflects the conditions of the in vivo environment with growing sprouts on cultured seed tubers. Further investigations together with the concomitant transcriptome analysis in the potato host will finally increase the understanding of a mutual relationship in the host pathogen interaction in an environment resembling the natural situation.

### Differentially expressed genes in isolate Ben3 comparing 3 and 8 dpi of potato sprouts

To differentiate between *R. solani* AG3-PT transcripts that were mainly relevant at early time point and those becoming more important at advanced stages of the interaction differentially expressed genes between 3 and 8 dpi of the interaction were also analyzed with DESeq2. Using the above mentioned criteria with adjusted P-values of less than 0.05 and a minimum fold change of |2| or more 173 genes could be assigned as differentially reduced expressed between 3 and 8 dpi while 400 genes are differentially increased expressed at the later timepoint. Complete lists of these genes together with their respective baseMean values and values of fold change are given in Supplementary Table 10, lists of the 20 most differentially expressed genes between 3 and 8 dpi are presented in Tables 3 and 4. While 10 out of the 20 most differentially reduced expressed genes between 3 and 8 dpi are coding for proteins involved in protein degradation and nitrogen uptake and assimilation (Table 3), the majority of genes differentially increased expressed between 3 and 8 dpi are involved in polysaccharide degradation with focus on copper-dependent lytic polysaccharide monooxygenases for cleavage of cellulose chains with oxidation of various carbons (Table 4).

**Table 3:**
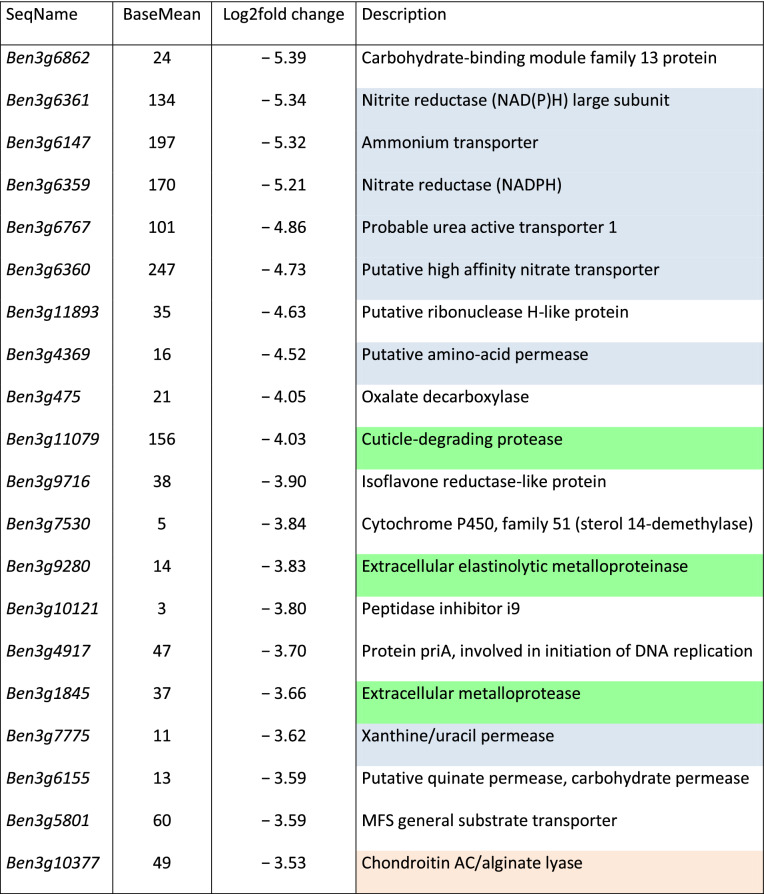
List of genes most differentially reduced expressed in *R. solani* AG3-PT isolate Ben3 between 3 and 8 dpi.

Log2fold change: calculated with the DESeq2 package as implemented in the ReadXplorer software.

Mean RPKM: average RPKM calculated with DESeq2 for both timepoints.

Description: Putative functions based on BLAST2GO protein BLAST. Blue, gene product involved in nitrogen uptake and assimilation; green, involved in protein degradation; orange, involved in polysaccharide degradation; grey, hypothetical protein.

**Table 4:**
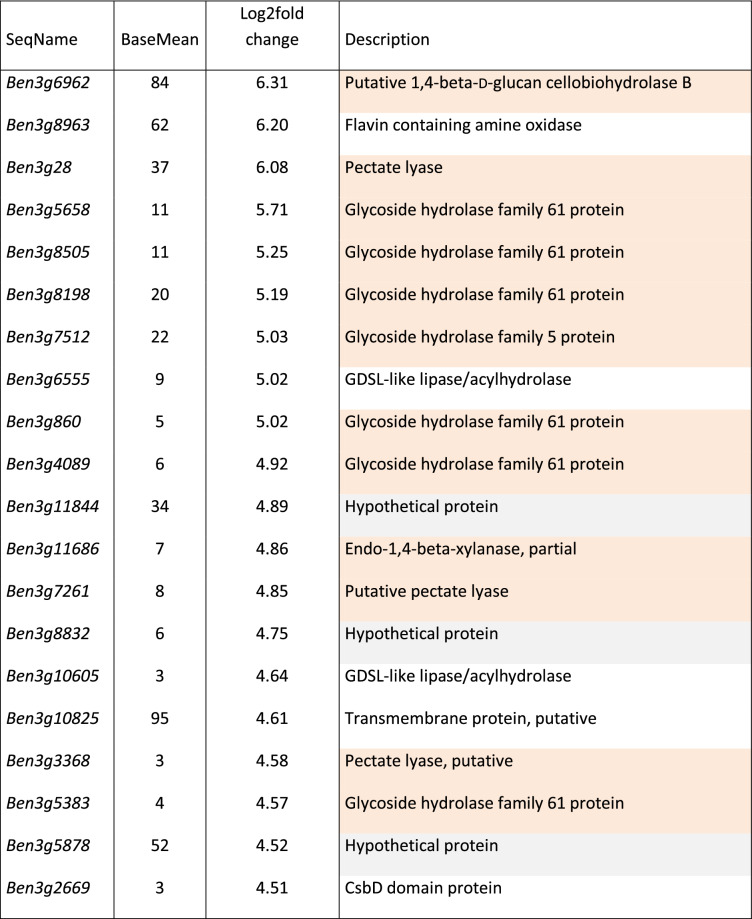
List of genes most differentially increased expressed in *R. solani* AG3-PT isolate Ben3 between 3 and 8 dpi.

Log2fold change: calculated with the DESeq2 package as implemented in the ReadXplorer software.

Mean RPKM: average RPKM calculated with DESeq2 for both timepoints.

Description: putative functions based on BLAST2GO protein BLAST. Orange, gene product involved in polysaccharide degradation; grey, hypothetical protein.

It is known that nitrogen metabolism and nitrogen-regulated gene expression in the plant pathogenic fungi is of great importance for the establishment of the disease in the host plant^58^. However, nitrate is the less preferred nitrogen source compared to ammonium and L-glutamine with respect to nutrient utilization in fungi at least during infection of leaves^59^. In fungi, this preferred nutrient utilization is regulated via nitrogen metabolite repression and ensures the transcription of ammonium and urea active permease encoding genes. Besides the strong transient expression of genes encoding ammonium and nitrogenous compounds transporting permeases (*Ben3g6147, Ben3g6767, Ben3g4369*, and Ben3g7775) at the early interaction stage at 3 dpi, *R. solani* AG3-PT isolate Ben3 exerted also high induction and expression of genes involved in nitrate uptake and assimilation (*Ben3g6360*, *Ben3g6359*, and *Ben3g6361*). Whether this is a distinctive feature of the isolate Ben3 or a characteristic of soil-borne plant pathogenic fungi would need further investigations.

The majority of genes with differentially increased expression between 3 and 8 dpi are involved in cell wall degradation coding for hydrolases acting on glycosyl bonds, and pectate lyases. This was expected and again demonstrates the increasing importance of breakdown of cell wall components designating the virulence strategy of a necrotrophic pathogen^21^.

## Conclusions

The results of the molecular responses of *R. solani* AG3-PT isolate Ben 3 during the interaction with a medium resistant host genotype of potato resemble the typical necrotrophic interaction. Specific induction of gene expression at the initial stages of interaction and during the course of interaction with the host plant was related to distinct functions. Expression induction of exporters facilitating excretion and strong upregulation of genes coding for peptidases and cell wall digesting enzymes was significant for the initial stage, while an additional increase of expression of cell wall hydrolyzing enzymes and genes encoding various integral membranes proteins with transporter function was linked to interaction progression. Interestingly, several Rhizoctonia specific yet unknown genes were differentially expressed, but further functional analysis will be needed to resolve their role in the *R. solani* AG3-PT potato interaction.

The used transcriptome sequencing approach allows addressing the expression response of the fungus as well as the plant simultaneously. In a following step the transcriptional answers of the potato sprouts towards the interaction with *R. solani* AG3-PT will give valuable information about the plants’ defense response. In addition, the same system to colonize the potato sprout with *R. solani* AG3-PT isolate Ben3 can be used with potato varieties showing differences in degree of field resistance to *R. solani* AG3-PT or to analyze the response of different *R. solani* AG3 isolates. A comparison of the interaction responses will lead the way to new candidates that can be used for the development of breeding strategies for new less susceptible varieties.

## Methods

### Plant material

The potato cultivar 'Arkula' (Norika GmbH, Sanitz, Germany) was used in this study. It has been demonstrated previously that 'Arkula' is susceptible to *R. solani* AG3-PT^25^, and is described as medium resistance to stem canker as stated in the European Cultivated Potato Database (https://www.europotato.org). To study interactions with *R. solani* AG3-PT, the use of pathogen free plant material is a prerequisite, thus potato mini-tubers were produced from in vitro plantlets (kindly provided by Norika GmbH). Mini-tubers were pre-sprouted in the dark using a cycle of changing temperatures (8 °C 1 week, 4 °C 3 weeks, 8 °C 3 days, 20–22 °C until sprouting) and a treatment with 10 mg/L gibberellic acid for 20 min. One mini-tuber was planted into each pot (pot size: 12 × 12 × 20 cm) containing a quartz sand/grit mixture (Euroquarz, Dorsten, Germany) and cultivated in a growth chamber (York, Mannheim, Germany) at 16/8 h day/night cycle at 18/15 °C day/night temperature and 400 μmol m^-2^ s^-1^ light and a relative humidity of 80%. The tubers were poured with B’cuzz Hydro A + B nutrient solution twice a week (Atami B.V., Rosmalen, The Netherlands), which had been adjusted to provide an EC of 2.1 dS m^-1^ and a pH of 5.8. If required, tubers were additionally watered with osmotic water. Each treatment (potato tubers without and with *R. solani* AG3-PT inoculation) included three replicates with ten plants per replicate, which were arranged in a completely randomized block design.

### Pathogen inoculation and sampling

The *R. solani* AG3-PT isolate Ben3 (kindly provided by Marianne Benker, North Rhine-Westphalia–Plant Protection Service, Germany) used in this study was cultured on Petri dishes on potato dextrose agar (PDA, Merck, Germany) at 20–22 °C for 5 days.

About 27 days after transferring mini-tubers into pots, when their sprouts had reached a length of 3–4 cm, an agar plug (Ø 10 mm) grown with isolate Ben3 was placed on the tuber next to the first emerging sprout^25^. Afterwards tubers were completely covered with quartz sand and each pot was sprayed with osmotic water to maintain humidity and promote fungal growth.

At both sampling dates [3 and 8 days post inoculation (dpi)], three biological replicate pools composed of all emerging sprouts from 10 plants per replicate were used. Subsequently, sprout samples were shock frozen in liquid nitrogen and stored at − 80 °C. All sprouts were examined regarding the incidence of necrotic lesions, while these lesions become first visible at the second sampling date.

For transcriptome analysis of the control isolate Ben3 mycelium without being attracted by a growing potato plant, the fungus was grown in liquid culture as follows: *R. solani* AG3-PT plugs (5 day old agar plugs of Ø 10 mm) were taken from the margin of a Petri dish culture and placed in 100 ml Erlenmeyer flasks in 30 ml of B’cuzz Hydro A + B nutrient solution, which was also used to culture the plants. The mycelium was grown without shaking for 8 days at the same time and conditions alongside the infected plants. Fungal hyphae were harvested and immediately shock frozen in liquid nitrogen and stored at − 80 °C prior to RNA extraction.

### RNA extraction

Prior to RNA extraction from inoculated sprout tissue the samples were ground using a mixer mill (2 min, 30/s; Retsch MM400, Haan, Germany) with two grinding balls (7 mm, 3 mm; Askubal, Korntal-Münchingen, Germany) under constant cooling in liquid nitrogen. Total RNA was extracted from 70 to 90 mg of ground sprout material using the RNeasy Plant Mini Kit (QIAGEN, Hilden, Germany) including DNase treatment (QIAGEN). After measuring the quantity of extracted RNA with the NanoDrop ND-1000 spectral photometer, quality and intactness of the RNA was analyzed using the bioanalyzer (Agilent Technologies Deutschland GmbH, Waldbronn, Germany).

RNA of fungal pellet was extracted using the same material and method.

### Sequencing

Sequencing of the prepared stranded cDNA libraries performed on an Illumina HiSeq 1500 platform (Illumina Inc., San Diego, U.S.A.). In total, 36 cDNA libraries were sequenced in six runs. The cDNA libraries were single-end sequenced in rapid mode with 1 × 50 cycles. Data analysis and base calling were accomplished with in-house software based on CASAVA 1.8.2^47^. In total 276 Gb data were obtained for 36 resulting libraries, with an average of 7.6 Gb mappings per library. The sequencing raw data for all libraries has been made available on the EBI ArrayExpress server, with the accession E-MTAB-7137, https://www.ebi.ac.uk/arrayexpress/experiments/E-MTAB-7137.

### Transcript mapping and expression analysis

Read mapping was carried out as described recently^30^. In brief, the obtained reads were quality filtered (> Q30) and subsequently mapped to the *R. solani* AG3-PT Ben3 draft genome^33^ [EMBL: FXZJ01000001-FXZJ01001390] using tophat2^60^. Two mismatches were allowed to handle possible sequencing errors and allelic variants of the diploid *R. solani* Ben3 genome. For transcript abundance analysis, resulting data were imported and analyzed using the ReadXplorer platform (v2.2)^43^. Reads per kilobase per million mapped reads (RPKM) values were calculated, using the single best match options (single perfect match mapping and single best match mapping of only uniquely mapped reads) for each of the separate libraries^61^. To determine the most abundant transcripts per sample, means of the RPKM values were recorded of the three biological replicates. As threshold, only transcripts with more than three raw reads in at least two of the three biological replicates were included and defined as being expressed.

For DESeq2 calculations^62^ the genes were counted as differentially expressed with an adjusted P-value of less than 0.05^63^ and a minimum fold change of 2 or more. Selected genes based on differential expression and RPKM results were annotated in detail using Blast2GO version 5.0^64^ applying default settings with an expect value of 1 × 10^–6^ and the fungiDB taxa 4751.

### Validating expression differences with RT-qPCR

RNA was extracted and quality controlled as described above. Single-stranded cDNA synthesis was carried out with 1 µg of total RNA using iScript cDNA Synthesis Kit (Bio-Rad Laboratories GmbH, Feldkirchen, Germany) in a 25 µl reaction following manufacturer´s instructions. Subsequently cDNA was diluted tenfold. RT-qPCR was performed using 96-well reaction plates on a Thermal Cycler CFX96 C1000 Touch (Bio-Rad). The thermal profile was 95 °C for 5 min, 40 cycles of 95 °C for 15 s and 60 °C for 1 min, followed by dsDNA melting curve analysis to ensure amplicon specificity. Each reaction was done in a 10 µL volume containing 200 nM of each primer, 3 µL of cDNA (1:10) and 5 µL of Sensi Fast SYBR NO ROX Kit (Bioline GmbH, Luckenwalde, Germany). Data collection and analysis was performed using CFX Manager Software 3.0 (Bio-Rad). At least three biological replicates were measured in duplicates, uninfected control plants and also non-template controls were included.

Relative transcript levels were normalized on the basis of expression of an invariant control. Therefore oligonucleotide primer sets of putative reference genes were tested with various infected plant samples and with pure mycelium grown without plant contact: Glyceraldehyde-3-phosphate dehydrogenase (*Ben3g7151*, *GAPDH*), Rs-GAPDHf: CATCATTCCATCGTCCACTG, Rs-GAPDHr: GAGGCAGATTTCTCCAATCG; Ubiquitin-conjugating enzyme E2 (*EUC63156*, *UBC*), Rs-UBCf: TAATCCAGGCGAGAGCAAGT, Rs-UBCr: CCCGCGATAGTTTAATCGAC; Ubiquitin-protein ligase E3 (*Ben3g494*, *UBI*), Rs-UBIf: AACGATTCTGGAGGGTTGTG, Rs-UBIr: GGTCATCGGACGTAGCATCT; Elongation factor 2 (*Ben3g3364*, *EF-2*), Rs-EF-2f: GTCTTCTCGGAAGAGCAACG, Rs-EF-2r: ACTCCCAGTGGTCAAAGACG; Beta tubulin (*Ben3g4099*; *TUB1*), Rs-TUB1f: ATGAAGGAAGTCGAGGAGCA, Rs-TUB1r: GCGGTAGAGTTGCCGATAAA; Beta tubulin (*Ben3g5288*, *TUB2*), Rs-TUB2f: AACTCGGCATCCTTCGTAGA, Rs-TUB2r: AGCAAATTGTCCATGGCTTC. While primer sets of *EF-2* and *TUB1* were not suitable because of amplification products in the uninfected plant background, primer sets of all other tested references gave reliable amplifications with efficiencies of close to 2. Reference gene stability was calculated with the CFX Manager software 3.0 (Bio-Rad) and with NormFinder^65^, selecting *TUB2* as the best invariant control.

ΔCq was calculated as the difference between control and target products (ΔCq = Cq_*gene*_ − Cq_*TUB2*_). Differences in relative expression levels between the treated samples were calculated as ΔΔCq = ΔCq (Ben3) − ΔCq (early or late). Oligonucleotide primer sets used for RT-qPCR are as follows: pectate lyase (*Ben3g3530*), Rs-3530f: TCCAACGTTATTGCAAACGA, Rs-3530r: GGCTCGTCACCATTGCTATT; extracellular metalloprotease (*Ben3g2070*), Rs-2070f: TTTCCCCTCCGACTATGCTA, Rs-2070r: CCCTGGAAGGTGTGGTAGAG; C2H2-type zinc-finger protein (*Ben3g4553*), Rs-4553f: CCCTCATGTGTGTGAGCACT, Rs-4553r: TGGACTGCGTCCTTCCTACT.

## Supplementary information

Supplementary Information.
