# Supplementary material for: Necrotrophic lifestyle of Rhizoctonia solani AG3-PT during interaction with its host plant potato as revealed by transcriptome analysis
Source: Sci Rep. 2020 Jul 28;10:12574. doi: 10.1038/s41598-020-68728-2 (PMC7387450; doi:10.1038/s41598-020-68728-2)
Supplement: Supplementary file 1 — Supplementary Information. [file 41598_2020_68728_MOESM1_ESM.pdf]

Necrotrophic lifestyle of *Rhizoctonia solani* AG3-PT during interaction with its host plant potato as revealed by transcriptome analysis

Rita Zrenner, Franziska Genzel, Bart Verwaaijen, Daniel Wibberg, Rita Grosch

Supplemental Table 1: List of genes exclusively transcribed in mycelium without plant contact

SeqName	RPKM	Description
Ben3g5404	15.70	hypothetical protein
Ben3g12171	15.57	hypothetical protein
Ben3g3378	14.59	hypothetical protein
Ben3g6615	14.11	Albumin-2
Ben3g6631	14.07	hypothetical protein
Ben3g4705	13.69	Diacetyl reductase [(S)-acetoin forming]
Ben3g9629	12.82	hypothetical protein
Ben3g11477	12.20	hypothetical protein
Ben3g11543	11.36	hypothetical protein
Ben3g5202	10.51	hypothetical protein
Ben3g9190	10.45	hypothetical protein
Ben3g5443	10.26	hypothetical protein
Ben3g8915	10.21	hypothetical protein
Ben3g6460	9.49	hypothetical protein
Ben3g5970	9.00	hypothetical protein
Ben3g9373	8.78	hypothetical protein
Ben3g8587	8.69	hypothetical protein
Ben3g10385	8.39	hypothetical protein
Ben3g10363	7.11	hypothetical protein
Ben3g8957	7.06	hypothetical protein
Ben3g8754	6.97	hypothetical protein
Ben3g6991	6.51	NADH dehydrogenase transmembrane subunit
Ben3g12536	6.36	3-oxoacyl-[acyl-carrier-protein] reductase FabG
Ben3g9385	6.12	hypothetical protein
Ben3g1702	5.92	hypothetical protein
Ben3g11337	5.80	Pre-mRNA-splicing factor rse1
Ben3g11133	5.77	hypothetical protein
Ben3g10681	5.70	hypothetical protein
Ben3g8235	5.68	hypothetical protein
Ben3g11127	5.56	hypothetical protein
Ben3g4803	5.34	hypothetical protein
Ben3g3408	5.33	hypothetical protein
Ben3g8376	5.27	hypothetical protein
Ben3g5659	5.07	hypothetical protein
Ben3g4161	5.01	hypothetical protein
Ben3g9851	4.99	Probable serine/threonine-protein kinase
Ben3g9206	4.56	hypothetical protein
Ben3g9262	4.54	Alpha-muurolene synthase
Ben3g9494	4.52	hypothetical protein
Ben3g9852	4.48	hypothetical protein
Ben3g6931	4.47	hypothetical protein
Ben3g9458	4.40	hypothetical protein
Ben3g10445	4.32	hypothetical protein
Ben3g6523	4.25	hypothetical protein
Ben3g10185	4.15	hypothetical protein
Ben3g10640	4.02	hypothetical protein
Ben3g12236	3.85	hypothetical protein

Ben3g12530	3.75	hypothetical protein
Ben3g2674	3.73	Lysine acetyltransferase
Ben3g12389	3.60	hypothetical protein
Ben3g11714	3.51	hypothetical protein
Ben3g11122	3.47	hypothetical protein
Ben3g11203	3.41	hypothetical protein
Ben3g6327	3.32	hypothetical protein
Ben3g11908	3.30	hypothetical protein
Ben3g12377	3.29	hypothetical protein
Ben3g6792	3.27	hypothetical protein
Ben3g11492	3.19	Putative glycine dehydrogenase
Ben3g8961	3.18	hypothetical protein
Ben3g11855	3.17	hypothetical protein
Ben3g10540	3.17	1,3-beta-glucan synthase component bgs3
Ben3g6816	3.13	hypothetical protein
Ben3g8491	3.12	hypothetical protein
Ben3g10577	3.12	Peptidyl-Lys metalloendopeptidase
Ben3g7654	3.11	hypothetical protein
Ben3g12518	3.10	hypothetical protein
Ben3g3479	3.10	hypothetical protein
Ben3g7694	3.09	Nitrogen assimilation transcription factor nit-4
Ben3g12020	3.02	hypothetical protein
Ben3g11339	3.00	hypothetical protein
Ben3g3236	2.98	1,3-beta-glucan synthase component FKS1
Ben3g3580	2.95	hypothetical protein
Ben3g4260	2.89	hypothetical protein
Ben3g1493	2.87	hypothetical protein
Ben3g12261	2.81	hypothetical protein
Ben3g10861	2.79	hypothetical protein
Ben3g12254	2.79	hypothetical protein
Ben3g10600	2.75	hypothetical protein
Ben3g10848	2.74	hypothetical protein
Ben3g6846	2.70	Uncharacterized WD repeat-containing protein
Ben3g10037	2.70	Cellulose-growth-specific protein
Ben3g10143	2.69	hypothetical protein
Ben3g4298	2.69	hypothetical protein
Ben3g2533	2.68	hypothetical protein
Ben3g10847	2.65	hypothetical protein
Ben3g6942	2.60	hypothetical protein
Ben3g9421	2.59	hypothetical protein
Ben3g12212	2.59	hypothetical protein
Ben3g8958	2.55	hypothetical protein
Ben3g6722	2.54	hypothetical protein
Ben3g5193	2.52	hypothetical protein
Ben3g5880	2.49	hypothetical protein
Ben3g7856	2.49	hypothetical protein
Ben3g10994	2.45	hypothetical protein
Ben3g12081	2.45	hypothetical protein
Ben3g10602	2.40	hypothetical protein
Ben3g10834	2.40	hypothetical protein

Ben3g3808	2.39	hypothetical protein
Ben3g10089	2.31	hypothetical protein
Ben3g11685	2.27	hypothetical protein
Ben3g1750	2.23	hypothetical protein
Ben3g9810	2.23	hypothetical protein
Ben3g11656	2.22	hypothetical protein
Ben3g7985	2.20	hypothetical protein
Ben3g12390	2.18	hypothetical protein
Ben3g45	2.14	hypothetical protein
Ben3g10419	2.10	hypothetical protein
Ben3g6075	2.10	hypothetical protein
Ben3g12155	2.07	hypothetical protein
Ben3g9501	2.06	hypothetical protein
Ben3g10740	2.05	ATPase 11, plasma membrane-type
Ben3g4765	2.04	hypothetical protein
Ben3g9417	2.01	hypothetical protein
Ben3g6374	2.01	Putative 30S ribosomal protein S17P-like
Ben3g9972	1.99	hypothetical protein
Ben3g809	1.97	hypothetical protein
Ben3g8519	1.96	hypothetical protein
Ben3g768	1.95	Hydroxyacyl-coenzyme A dehydrogenase, mitochondrial
Ben3g4336	1.95	DNA repair protein RAD2
Ben3g9899	1.92	E3 ubiquitin-protein ligase RNF14
Ben3g7844	1.90	hypothetical protein
Ben3g4721	1.86	hypothetical protein
Ben3g8597	1.84	hypothetical protein
Ben3g11516	1.82	hypothetical protein
Ben3g12292	1.81	hypothetical protein
Ben3g4159	1.78	hypothetical protein
Ben3g9452	1.77	hypothetical protein
Ben3g11340	1.77	hypothetical protein
Ben3g12375	1.76	Tyrosine-protein kinase CSK
Ben3g4561	1.75	hypothetical protein
Ben3g7898	1.75	Uncharacterized protein
Ben3g12189	1.75	hypothetical protein
Ben3g979	1.74	hypothetical protein
Ben3g12279	1.73	hypothetical protein
Ben3g10906	1.73	hypothetical protein
Ben3g5666	1.73	hypothetical protein
Ben3g12178	1.72	hypothetical protein
Ben3g11031	1.72	hypothetical protein
Ben3g12328	1.71	hypothetical protein
Ben3g11156	1.71	hypothetical protein
Ben3g12013	1.69	hypothetical protein
Ben3g10578	1.68	hypothetical protein
Ben3g7137	1.68	hypothetical protein
Ben3g10997	1.65	hypothetical protein
Ben3g12467	1.65	hypothetical protein
Ben3g8669	1.64	hypothetical protein
Ben3g11814	1.64	hypothetical protein

Ben3g11162	1.64	hypothetical protein
Ben3g10427	1.63	hypothetical protein
Ben3g11518	1.62	hypothetical protein
Ben3g7257	1.61	Uncharacterized protein
Ben3g7593	1.60	hypothetical protein
Ben3g12313	1.60	hypothetical protein
Ben3g6372	1.58	hypothetical protein
Ben3g6655	1.58	hypothetical protein
Ben3g10262	1.57	hypothetical protein
Ben3g550	1.56	Pectinesterase
Ben3g9748	1.54	hypothetical protein
Ben3g12407	1.54	Serine/threonine-protein kinase
Ben3g4345	1.53	hypothetical protein
Ben3g7026	1.51	hypothetical protein
Ben3g11725	1.50	hypothetical protein
Ben3g1940	1.50	hypothetical protein
Ben3g370	1.48	hypothetical protein
Ben3g2864	1.48	hypothetical protein
Ben3g3358	1.47	hypothetical protein
Ben3g3504	1.47	hypothetical protein
Ben3g9866	1.47	hypothetical protein
Ben3g6373	1.45	hypothetical protein
Ben3g11978	1.44	hypothetical protein
Ben3g9561	1.41	H-type lectin domain
Ben3g10631	1.40	hypothetical protein
Ben3g8275	1.38	hypothetical protein
Ben3g4206	1.38	hypothetical protein
Ben3g6452	1.38	Cyclin-dependent kinase 5 homolog
Ben3g7225	1.37	hypothetical protein
Ben3g9201	1.37	hypothetical protein
Ben3g10389	1.37	hypothetical protein
Ben3g10184	1.37	hypothetical protein
Ben3g10535	1.36	hypothetical protein
Ben3g10070	1.36	Retrovirus-related Pol polyprotein transposon TNT 1-94
Ben3g1607	1.35	hypothetical protein
Ben3g5713	1.34	hypothetical protein
Ben3g4820	1.34	hypothetical protein
Ben3g11373	1.34	hypothetical protein
Ben3g4753	1.33	hypothetical protein
Ben3g8570	1.33	hypothetical protein
Ben3g12232	1.32	hypothetical protein
Ben3g6627	1.31	hypothetical protein
Ben3g9684	1.30	hypothetical protein
Ben3g10798	1.29	hypothetical protein
Ben3g11629	1.29	Putative 30S ribosomal protein S17P-like
Ben3g11848	1.29	hypothetical protein
Ben3g4899	1.29	hypothetical protein
Ben3g11854	1.29	hypothetical protein
Ben3g6517	1.28	dynein heavy chain
Ben3g8234	1.28	hypothetical protein

Ben3g9245	1.28	hypothetical protein
Ben3g8217	1.26	hypothetical protein
Ben3g8910	1.25	Probable xyloglucan-specific endo-beta-1,4-glucanase A
Ben3g10533	1.24	Nucleoside-triphosphate binding/transmembrane receptor
Ben3g7668	1.23	hypothetical protein
Ben3g10868	1.23	hypothetical protein
Ben3g10894	1.22	hypothetical protein
Ben3g5388	1.22	hypothetical protein
Ben3g7548	1.20	hypothetical protein
Ben3g1083	1.20	Meiotic recombination protein rec8
Ben3g11522	1.20	hypothetical protein
Ben3g10403	1.19	hypothetical protein
Ben3g2957	1.19	hypothetical protein
Ben3g9523	1.19	hypothetical protein
Ben3g4162	1.18	Calcium-channel protein cch1
Ben3g8974	1.18	hypothetical protein
Ben3g7352	1.16	Putative 30S ribosomal protein S17P-like
Ben3g8816	1.16	hypothetical protein
Ben3g9801	1.16	hypothetical protein
Ben3g10204	1.16	hypothetical protein
Ben3g1679	1.16	hypothetical protein
Ben3g7193	1.15	hypothetical protein
Ben3g12525	1.15	hypothetical protein
Ben3g417	1.15	Putative NADPH-dependent methylglyoxal reductase
Ben3g5726	1.15	hypothetical protein
Ben3g3538	1.14	hypothetical protein
Ben3g11172	1.13	Uncharacterized WD repeat-containing protein
Ben3g12324	1.11	hypothetical protein
Ben3g7730	1.11	O-methylsterigmatocystin oxidoreductase
Ben3g10253	1.11	hypothetical protein
Ben3g4749	1.10	Subtilisin-like protease
Ben3g9784	1.10	hypothetical protein
Ben3g12127	1.10	hypothetical protein
Ben3g10141	1.10	hypothetical protein
Ben3g6950	1.10	hypothetical protein
Ben3g9355	1.09	Manganese peroxidase 2
Ben3g10259	1.09	hypothetical protein
Ben3g10981	1.08	hypothetical protein
Ben3g4406	1.08	Probable alpha-galactosidase B
Ben3g1503	1.08	hypothetical protein
Ben3g11642	1.07	hypothetical protein
Ben3g9512	1.06	hypothetical protein
Ben3g965	1.06	LRR receptor-like serine/threonine-protein kinase
Ben3g4192	1.06	hypothetical protein
Ben3g3356	1.05	hypothetical protein
Ben3g9651	1.03	hypothetical protein
Ben3g12366	1.02	Serine/threonine-protein kinase HT1
Ben3g11559	1.02	hypothetical protein
Ben3g11897	1.01	hypothetical protein
Ben3g6941	1.01	hypothetical protein

Ben3g3588	1.01	hypothetical protein
Ben3g4835	0.99	hypothetical protein
Ben3g11412	0.99	hypothetical protein
Ben3g9289	0.98	hypothetical protein
Ben3g1080	0.98	hypothetical protein
Ben3g11975	0.97	hypothetical protein
Ben3g8029	0.95	hypothetical protein
Ben3g1659	0.95	hypothetical protein
Ben3g11344	0.95	hypothetical protein
Ben3g11290	0.94	Vegetative incompatibility protein HET-E-1
Ben3g11160	0.93	hypothetical protein
Ben3g10765	0.90	hypothetical protein
Ben3g2993	0.89	hypothetical protein
Ben3g6431	0.89	Tigger transposable element-derived protein 6
Ben3g10056	0.89	hypothetical protein
Ben3g7472	0.89	hypothetical protein
Ben3g38	0.89	hypothetical protein
Ben3g8316	0.89	hypothetical protein
Ben3g7965	0.89	WD repeat domain 17
Ben3g6819	0.88	Kinesin light chain
Ben3g10998	0.88	hypothetical protein
Ben3g12488	0.86	hypothetical protein
Ben3g11947	0.86	hypothetical protein
Ben3g12528	0.86	Vegetative incompatibility protein HET-E-1
Ben3g9918	0.86	hypothetical protein
Ben3g3663	0.86	hypothetical protein
Ben3g5453	0.85	hypothetical protein
Ben3g11510	0.85	hypothetical protein
Ben3g6136	0.85	hypothetical protein
Ben3g10209	0.84	hypothetical protein
Ben3g11722	0.82	Mitogen-activated protein kinase kinase 6
Ben3g9881	0.82	hypothetical protein
Ben3g4128	0.82	hypothetical protein
Ben3g9263	0.82	hypothetical protein
Ben3g4639	0.81	Probable succinyl-CoA:3-ketoacid coenzyme A transferase
Ben3g7594	0.81	hypothetical protein
Ben3g1851	0.80	hypothetical protein
Ben3g8548	0.80	Putative 30S ribosomal protein S17P-like
Ben3g8944	0.79	hypothetical protein
Ben3g4774	0.79	hypothetical protein
Ben3g4626	0.78	hypothetical protein
Ben3g8215	0.78	hypothetical protein
Ben3g6053	0.77	hypothetical protein
Ben3g7610	0.77	hypothetical protein
Ben3g11641	0.76	hypothetical protein
Ben3g3888	0.76	hypothetical protein
Ben3g6719	0.76	hypothetical protein
Ben3g10280	0.75	hypothetical protein
Ben3g12373	0.75	hypothetical protein
Ben3g6237	0.75	hypothetical protein

Ben3g8411	0.75	hypothetical protein
Ben3g4649	0.74	hypothetical protein
Ben3g1555	0.73	Repressor ROX1
Ben3g11383	0.73	hypothetical protein
Ben3g9516	0.71	hypothetical protein
Ben3g12112	0.71	hypothetical protein
Ben3g11427	0.70	hypothetical protein
Ben3g11872	0.69	hypothetical protein
Ben3g6721	0.68	hypothetical protein
Ben3g3572	0.68	hypothetical protein
Ben3g5548	0.68	hypothetical protein
Ben3g7070	0.68	hypothetical protein
Ben3g5687	0.67	hypothetical protein
Ben3g12202	0.66	hypothetical protein
Ben3g12456	0.66	hypothetical protein
Ben3g6576	0.66	hypothetical protein xynA
Ben3g39	0.66	hypothetical protein
Ben3g8633	0.65	hypothetical protein
Ben3g7258	0.65	Transposable element Tcb2 transposase
Ben3g10090	0.65	Ca2+:H+ antiporter
Ben3g2965	0.65	hypothetical protein
Ben3g6563	0.64	hypothetical protein
Ben3g5458	0.64	hypothetical protein
Ben3g3111	0.64	hypothetical protein
Ben3g12265	0.64	Probable rhamnogalacturonase B
Ben3g6132	0.63	hypothetical protein
Ben3g11530	0.63	hypothetical protein
Ben3g11428	0.63	hypothetical protein
Ben3g2846	0.63	hypothetical protein
Ben3g10069	0.63	hypothetical protein
Ben3g6394	0.62	hypothetical protein
Ben3g11126	0.62	hypothetical protein
Ben3g11651	0.62	hypothetical protein
Ben3g10910	0.62	hypothetical protein
Ben3g10110	0.61	hypothetical protein
Ben3g9170	0.61	hypothetical protein
Ben3g7185	0.61	Cold shock-like protein CspG
Ben3g8308	0.61	hypothetical protein
Ben3g200	0.61	hypothetical protein
Ben3g11232	0.61	hypothetical protein
Ben3g11845	0.60	Vegetative incompatibility protein HET-E-1
Ben3g6632	0.60	hypothetical protein
Ben3g12233	0.60	hypothetical protein
Ben3g5724	0.60	Protein transport protein SEC9
Ben3g11998	0.60	hypothetical protein
Ben3g11130	0.59	hypothetical protein
Ben3g8057	0.59	ATP-dependent DNA helicase PcrA
Ben3g12434	0.59	hypothetical protein
Ben3g8955	0.58	hypothetical protein
Ben3g424	0.58	hypothetical protein

Ben3g7371	0.58	hypothetical protein
Ben3g8567	0.57	hypothetical protein
Ben3g11892	0.57	hypothetical protein
Ben3g10515	0.57	hypothetical protein
Ben3g8446	0.57	hypothetical protein
Ben3g6803	0.56	hypothetical protein
Ben3g6375	0.56	hypothetical protein
Ben3g5146	0.56	hypothetical protein
Ben3g10915	0.56	hypothetical protein
Ben3g12331	0.56	hypothetical protein
Ben3g12165	0.55	hypothetical protein
Ben3g5433	0.55	hypothetical protein
Ben3g2607	0.55	Hydroxyacylglutathione hydrolase
Ben3g5254	0.55	hypothetical protein
Ben3g10528	0.55	recC exodeoxyribonuclease V, gamma subunit
Ben3g12520	0.55	hypothetical protein
Ben3g9609	0.55	hypothetical protein
Ben3g1792	0.55	E3 ubiquitin-protein ligase
Ben3g4191	0.55	hypothetical protein
Ben3g4931	0.54	hypothetical protein
Ben3g11538	0.54	hypothetical protein
Ben3g3184	0.54	hypothetical protein
Ben3g1752	0.54	hypothetical protein
Ben3g6961	0.53	hypothetical protein
Ben3g8283	0.53	hypothetical protein
Ben3g5351	0.52	hypothetical protein
Ben3g7715	0.52	hypothetical protein
Ben3g10701	0.52	hypothetical protein
Ben3g10129	0.52	hypothetical protein
Ben3g11353	0.52	hypothetical protein
Ben3g11358	0.52	similar to zinc finger protein
Ben3g8456	0.52	hypothetical protein
Ben3g11862	0.51	hypothetical protein
Ben3g8943	0.51	hypothetical protein
Ben3g4629	0.51	Polyporopepsin
Ben3g11313	0.51	hypothetical protein
Ben3g1703	0.51	hypothetical protein
Ben3g11473	0.51	hypothetical protein
Ben3g2596	0.50	hypothetical protein
Ben3g11506	0.49	hypothetical protein
Ben3g11291	0.49	hypothetical protein
Ben3g6671	0.49	hypothetical protein
Ben3g58	0.49	hypothetical protein
Ben3g10043	0.49	hypothetical protein
Ben3g11114	0.49	hypothetical protein
Ben3g11793	0.48	hypothetical protein
Ben3g9070	0.48	Casein kinase I homolog 1
Ben3g11675	0.47	hypothetical protein
Ben3g12314	0.47	Putative 30S ribosomal protein S17P-like
Ben3g11047	0.47	hypothetical protein

Ben3g5321	0.47	hypothetical protein
Ben3g11499	0.46	Uncharacterized WD repeat-containing protein
Ben3g567	0.46	hypothetical protein
Ben3g11459	0.46	Translation initiation factor eIF-4F
Ben3g10893	0.45	hypothetical protein
Ben3g8940	0.45	hypothetical protein
Ben3g9407	0.44	hypothetical protein
Ben3g7937	0.44	hypothetical protein
Ben3g11	0.44	hypothetical protein
Ben3g12317	0.43	hypothetical protein
Ben3g2487	0.43	hypothetical protein
Ben3g9069	0.43	hypothetical protein
Ben3g6528	0.43	hypothetical protein
Ben3g7382	0.43	hypothetical protein
Ben3g10423	0.43	Vegetative incompatibility protein HET-E-1
Ben3g5875	0.42	hypothetical protein
Ben3g12420	0.42	hypothetical protein
Ben3g1025	0.42	Carboxylesterase B
Ben3g4733	0.42	Drug resistance protein YOR378W
Ben3g10352	0.42	hypothetical protein
Ben3g9274	0.42	hypothetical protein
Ben3g4029	0.41	hypothetical protein
Ben3g4559	0.41	hypothetical protein
Ben3g4485	0.41	hypothetical protein
Ben3g5653	0.41	hypothetical protein
Ben3g7933	0.41	Epoxide hydrolase, putative
Ben3g6238	0.41	hypothetical protein
Ben3g11515	0.40	hypothetical protein
Ben3g556	0.40	hypothetical protein
Ben3g9562	0.40	H-type lectin domain
Ben3g12546	0.40	hypothetical protein
Ben3g10758	0.40	hypothetical protein
Ben3g6378	0.40	hypothetical protein
Ben3g8466	0.39	Tyrosine-protein kinase FRK
Ben3g8618	0.39	hypothetical protein
Ben3g12497	0.39	hypothetical protein
Ben3g5370	0.39	hypothetical protein
Ben3g11167	0.39	hypothetical protein
Ben3g9525	0.38	hypothetical protein
Ben3g8616	0.38	hypothetical protein
Ben3g7795	0.38	hypothetical protein
Ben3g10486	0.38	hypothetical protein
Ben3g12395	0.37	hypothetical protein
Ben3g10784	0.37	hypothetical protein
Ben3g12124	0.37	hypothetical protein
Ben3g12290	0.37	hypothetical protein
Ben3g6737	0.37	hypothetical protein
Ben3g6407	0.37	hypothetical protein
Ben3g5255	0.37	hypothetical protein
Ben3g12539	0.36	hypothetical protein

Ben3g10668	0.36	hypothetical protein
Ben3g4271	0.36	O-methylsterigmatocystin oxidoreductase
Ben3g880	0.36	hypothetical protein
Ben3g7175	0.36	hypothetical protein
Ben3g3359	0.36	hypothetical protein
Ben3g7052	0.36	hypothetical protein
Ben3g1087	0.35	hypothetical protein
Ben3g10293	0.35	hypothetical protein
Ben3g11526	0.35	hypothetical protein
Ben3g9446	0.35	hypothetical protein
Ben3g10011	0.35	Uncharacterized protein
Ben3g2568	0.35	hypothetical protein
Ben3g9168	0.34	hypothetical protein
Ben3g3032	0.34	hypothetical protein
Ben3g9093	0.34	Retrovirus-related Pol polyprotein transposon TNT 1-94
Ben3g6951	0.34	hypothetical protein
Ben3g9161	0.34	hypothetical protein
Ben3g3237	0.34	ATPase 10, plasma membrane-type
Ben3g2786	0.34	hypothetical protein
Ben3g9617	0.34	hypothetical protein
Ben3g7788	0.33	hypothetical protein
Ben3g5861	0.33	hypothetical protein
Ben3g9950	0.33	hypothetical protein
Ben3g12339	0.33	Uncharacterized WD repeat-containing protein
Ben3g11972	0.33	hypothetical protein
Ben3g11973	0.33	hypothetical protein
Ben3g11245	0.32	hypothetical protein
Ben3g6966	0.32	hypothetical protein
Ben3g10548	0.32	hypothetical protein
Ben3g6720	0.31	hypothetical protein
Ben3g7792	0.31	Calcium-independent phospholipase A2-gamma
Ben3g10654	0.31	hypothetical protein
Ben3g1878	0.31	hypothetical protein
Ben3g2202	0.31	hypothetical protein
Ben3g11481	0.31	hypothetical protein
Ben3g6121	0.31	hypothetical protein
Ben3g9108	0.30	Vegetative incompatibility protein HET-E-1
Ben3g9655	0.30	hypothetical protein
Ben3g11765	0.30	Bloom syndrome protein homolog
Ben3g11264	0.30	Vegetative incompatibility protein HET-E-1
Ben3g6852	0.30	Peptidyl-Lys metalloendopeptidase
Ben3g2959	0.30	Zinc-binding alcohol dehydrogenase domain protein cipB
Ben3g11602	0.30	hypothetical protein
Ben3g7981	0.30	hypothetical protein
Ben3g10739	0.30	hypothetical protein
Ben3g8240	0.30	hypothetical protein
Ben3g10180	0.29	hypothetical protein
Ben3g1603	0.29	hypothetical protein
Ben3g11740	0.29	hypothetical protein
Ben3g8309	0.29	hypothetical protein

Ben3g9445	0.28	Uncharacterized WD repeat-containing protein
Ben3g1870	0.28	Isoflavone reductase-like protein
Ben3g7770	0.28	hypothetical protein
Ben3g8942	0.27	hypothetical protein
Ben3g11824	0.27	hypothetical protein
Ben3g8463	0.27	Multicomponent K+:H+ antiporter subunit A
Ben3g12438	0.27	hypothetical protein
Ben3g7777	0.27	hypothetical protein
Ben3g4168	0.27	hypothetical protein
Ben3g12211	0.27	hypothetical protein
Ben3g8042	0.27	hypothetical protein, Jacalin-like lectin domain
Ben3g10875	0.27	hypothetical protein
Ben3g7575	0.27	hypothetical protein
Ben3g5505	0.26	hypothetical protein
Ben3g10002	0.26	hypothetical protein, Tetratricopeptide repeat
Ben3g4169	0.26	hypothetical protein
Ben3g11410	0.26	hypothetical protein
Ben3g7289	0.26	hypothetical protein
Ben3g9721	0.26	hypothetical protein
Ben3g11585	0.26	General transcriptional corepressor ssn6
Ben3g12042	0.26	hypothetical protein
Ben3g6658	0.25	hypothetical protein
Ben3g10416	0.25	Uncharacterized WD repeat-containing protein
Ben3g10242	0.25	Casein kinase I
Ben3g10073	0.25	hypothetical protein
Ben3g10869	0.25	hypothetical protein
Ben3g3557	0.25	hypothetical protein
Ben3g7005	0.25	hypothetical protein
Ben3g4645	0.25	hypothetical protein
Ben3g9349	0.25	hypothetical protein
Ben3g8461	0.25	hypothetical protein
Ben3g6203	0.25	hypothetical protein
Ben3g8281	0.25	Protein RTA1
Ben3g8551	0.24	hypothetical protein
Ben3g4198	0.24	hypothetical protein
Ben3g11546	0.24	hypothetical protein
Ben3g7041	0.24	hypothetical protein
Ben3g6579	0.23	NADPH-dependent methylglyoxal reductase GRE2
Ben3g10760	0.23	hypothetical protein
Ben3g10030	0.23	hypothetical protein
Ben3g5661	0.23	hypothetical protein
Ben3g11618	0.23	hypothetical protein
Ben3g1669	0.23	hypothetical protein
Ben3g11015	0.23	hypothetical protein
Ben3g3561	0.22	NADPH 1-acyldihydroxyacetone phosphate reductase
Ben3g8252	0.22	hypothetical protein
Ben3g6620	0.22	Probable serine/threonine-protein kinase
Ben3g12397	0.22	hypothetical protein
Ben3g11374	0.22	hypothetical protein
Ben3g9947	0.22	hypothetical protein

Ben3g1871	0.22	Isoflavone reductase homolog P3
Ben3g9195	0.22	hypothetical protein
Ben3g2346	0.22	hypothetical protein
Ben3g11980	0.22	similar to putative tyrosine recombinase
Ben3g7229	0.21	hypothetical protein
Ben3g8706	0.21	hypothetical protein
Ben3g9665	0.21	Uncharacterized protein
Ben3g11158	0.21	hypothetical protein
Ben3g10542	0.21	mynn, non-specific serine/threonine protein kinase
Ben3g4648	0.21	Protein RTA1
Ben3g7493	0.21	hypothetical protein
Ben3g8546	0.20	MutS protein homolog 4
Ben3g10845	0.20	UDP-glucose 4-epimerase
Ben3g5890	0.20	Cytochrome P450 3A6
Ben3g7442	0.20	hypothetical protein
Ben3g7639	0.20	hypothetical protein
Ben3g12083	0.20	hypothetical protein
Ben3g2791	0.20	hypothetical protein
Ben3g944	0.20	hypothetical protein
Ben3g9080	0.20	Uncharacterized WD repeat-containing protein
Ben3g4653	0.20	Metacaspase-1A
Ben3g6358	0.19	hypothetical protein
Ben3g9353	0.19	Rhamnogalacturonan acetylesterase
Ben3g10541	0.19	hypothetical protein
Ben3g9000	0.19	hypothetical protein
Ben3g10153	0.19	Putative 30S ribosomal protein S17P-like
Ben3g3037	0.18	hypothetical protein
Ben3g9576	0.18	Acyl-CoA desaturase
Ben3g12392	0.18	hypothetical protein
Ben3g5854	0.18	Probable transporter MCH4
Ben3g9288	0.18	hypothetical protein
Ben3g12226	0.18	hypothetical protein
Ben3g2969	0.17	hypothetical protein
Ben3g6047	0.17	O-methylsterigmatocystin oxidoreductase
Ben3g9739	0.17	hypothetical protein
Ben3g12447	0.17	hypothetical protein
Ben3g11531	0.17	hypothetical protein
Ben3g8414	0.17	hypothetical protein
Ben3g2262	0.17	hypothetical protein
Ben3g11022	0.17	Ovarian abundant message protein
Ben3g4764	0.17	hypothetical protein
Ben3g10126	0.17	O-methylsterigmatocystin oxidoreductase
Ben3g3673	0.17	hypothetical protein
Ben3g2847	0.17	hypothetical protein
Ben3g9643	0.17	hypothetical protein
Ben3g3003	0.16	hypothetical protein
Ben3g1013	0.16	Flavin-containing monooxygenase FMO GS-OX3
Ben3g4063	0.16	Chitinase 1
Ben3g10109	0.16	hypothetical protein
Ben3g4686	0.16	hypothetical protein

Ben3g10216	0.16	Structure-specific endonuclease subunit SLX1
Ben3g10460	0.16	hypothetical protein
Ben3g9648	0.16	hypothetical protein
Ben3g7542	0.16	hypothetical protein
Ben3g11565	0.16	hypothetical protein
Ben3g11970	0.16	hypothetical protein
Ben3g4737	0.16	hypothetical protein
Ben3g587	0.16	hypothetical protein
Ben3g7425	0.15	hypothetical protein
Ben3g5789	0.15	Aminomethyltransferase
Ben3g4488	0.15	Probable iron/ascorbate oxidoreductase
Ben3g12380	0.15	hypothetical protein
Ben3g10831	0.15	hypothetical protein
Ben3g4160	0.15	hypothetical protein
Ben3g7846	0.15	hypothetical protein
Ben3g7044	0.15	Thaumatin-like protein 1a
Ben3g482	0.15	hypothetical protein
Ben3g8554	0.15	hypothetical protein
Ben3g11775	0.15	hypothetical protein
Ben3g12215	0.15	hypothetical protein
Ben3g10231	0.14	hypothetical protein, Bacterial-like globin
Ben3g7458	0.14	Probable alpha-fucosidase A
Ben3g6784	0.14	hypothetical protein
Ben3g10763	0.14	hypothetical protein
Ben3g12204	0.14	hypothetical protein
Ben3g3033	0.14	hypothetical protein
Ben3g10279	0.14	Vegetative incompatibility protein HET-E-1
Ben3g10268	0.14	hypothetical protein
Ben3g3321	0.14	hypothetical protein
Ben3g8026	0.13	hypothetical protein
Ben3g7935	0.13	hypothetical protein
Ben3g1572	0.13	hypothetical protein
Ben3g9821	0.13	hypothetical protein
Ben3g11759	0.13	hypothetical protein
Ben3g5163	0.13	Probable receptor-like protein kinase
Ben3g11469	0.13	hypothetical protein
Ben3g6513	0.13	hypothetical protein
Ben3g9208	0.13	Eukaryotic translation initiation factor 4 gamma
Ben3g3035	0.13	hypothetical protein
Ben3g12126	0.13	Probable glucan endo-1,6-beta-glucosidase B
Ben3g3	0.13	hypothetical protein
Ben3g9366	0.13	Probable rhamnogalacturonate lyase A
Ben3g11068	0.13	hypothetical protein
Ben3g5907	0.12	hypothetical protein
Ben3g9567	0.12	H-type lectin domain
Ben3g6342	0.12	hypothetical protein
Ben3g2509	0.12	hypothetical protein
Ben3g11707	0.12	hypothetical protein
Ben3g11776	0.12	cellulose synthase catalytic subunit (EC:2.4.1.12)
Ben3g9448	0.12	Vegetative incompatibility protein HET-E-1

Ben3g3305	0.12	lcc8 laccase, multicopper oxidase
Ben3g10064	0.12	hypothetical protein
Ben3g9503	0.12	hypothetical protein
Ben3g5910	0.11	hypothetical protein
Ben3g11001	0.11	hypothetical protein
Ben3g12160	0.11	hypothetical protein
Ben3g4621	0.11	hypothetical protein
Ben3g7960	0.11	hypothetical protein
Ben3g7440	0.11	Transposable element Tc1 transposase
Ben3g12240	0.11	hypothetical protein
Ben3g11919	0.11	hypothetical protein
Ben3g8829	0.11	hypothetical protein
Ben3g8755	0.11	hypothetical protein
Ben3g5496	0.11	hypothetical protein
Ben3g12095	0.11	hypothetical protein
Ben3g12451	0.11	hypothetical protein
Ben3g8545	0.10	MutS protein homolog 4
Ben3g4829	0.10	CENP-B homolog protein 2
Ben3g11179	0.10	hypothetical protein
Ben3g9723	0.10	hypothetical protein
Ben3g36	0.10	hypothetical protein
Ben3g8256	0.10	hypothetical protein
Ben3g7825	0.10	hypothetical protein
Ben3g425	0.10	hypothetical protein
Ben3g8624	0.10	Benzoate 4-monooxygenase
Ben3g4583	0.10	nitric-oxide reductase subunit B
Ben3g9916	0.10	hypothetical protein
Ben3g6492	0.10	hypothetical protein
Ben3g12110	0.10	Vegetative incompatibility protein HET-E-1
Ben3g409	0.10	Probable transporter MCH1
Ben3g9504	0.10	hypothetical protein
Ben3g2511	0.10	hypothetical protein
Ben3g12031	0.10	hypothetical protein
Ben3g5167	0.10	Serine/threonine-protein kinase Chk2
Ben3g7441	0.10	Probable RNA-directed DNA polymerase
Ben3g6422	0.10	Probable serine/threonine-protein kinase
Ben3g10166	0.09	hypothetical protein
Ben3g10686	0.09	hypothetical protein
Ben3g8978	0.09	hypothetical protein
Ben3g6663	0.09	hypothetical protein
Ben3g12398	0.09	hypothetical protein
Ben3g12054	0.09	hypothetical protein
Ben3g6536	0.09	Superoxide dismutase [Cu-Zn]
Ben3g12435	0.09	Cytochrome P450 4C1
Ben3g11509	0.09	hypothetical protein
Ben3g12222	0.09	Mitogen-activated protein kinase kinase kinase ANP1
Ben3g9408	0.09	Extracellular protease
Ben3g4713	0.09	Glucosylceramidase
Ben3g10644	0.09	hypothetical protein
Ben3g5906	0.09	hypothetical protein

Ben3g5600	0.09	hypothetical protein
Ben3g5940	0.09	hypothetical protein
Ben3g12218	0.09	hypothetical protein
Ben3g5249	0.09	Cell wall protein PRY3
Ben3g10323	0.09	hypothetical protein
Ben3g10649	0.09	hypothetical protein
Ben3g1714	0.09	hypothetical protein
Ben3g8226	0.09	hypothetical protein
Ben3g5315	0.09	hypothetical protein
Ben3g6679	0.09	hypothetical protein
Ben3g1601	0.09	hypothetical protein
Ben3g11688	0.09	hypothetical protein
Ben3g8924	0.08	Retrotransposon-derived protein PEG10
Ben3g11955	0.08	hypothetical protein
Ben3g4580	0.08	Betaine lipid synthase
Ben3g11954	0.08	Putative AC transposase
Ben3g4657	0.08	hypothetical protein
Ben3g6402	0.08	Retrotransposon-derived protein PEG10
Ben3g11319	0.08	Protein-tyrosine-phosphatase
Ben3g11441	0.08	hypothetical protein
Ben3g5038	0.08	hypothetical protein
Ben3g11438	0.08	Probable transporter MCH2
Ben3g6042	0.08	hypothetical protein
Ben3g6978	0.08	Zinc-type alcohol dehydrogenase-like protein C2E1P3.01
Ben3g11178	0.08	hypothetical protein
Ben3g8412	0.08	Casein kinase I isoform alpha
Ben3g9243	0.08	Vegetative incompatibility protein HET-E-1
Ben3g4300	0.08	GMP synthase [glutamine-hydrolyzing]
Ben3g11574	0.07	NADH dehydrogenase transmembrane subunit
Ben3g12182	0.07	hypothetical protein
Ben3g11521	0.07	hypothetical protein
Ben3g7716	0.07	Retrovirus-related Pol polyprotein transposon TNT 1-94
Ben3g9889	0.07	hypothetical protein
Ben3g11318	0.07	hypothetical protein
Ben3g8566	0.07	hypothetical protein
Ben3g7559	0.07	Polyporopepsin
Ben3g981	0.07	hypothetical protein
Ben3g7457	0.07	Alpha-fucosidase A
Ben3g11769	0.07	hypothetical protein
Ben3g1310	0.07	Arabinan endo-1,5-alpha-L-arabinosidase
Ben3g9505	0.07	hypothetical protein
Ben3g12533	0.07	hypothetical protein
Ben3g10006	0.07	Minor extracellular protease vpr
Ben3g6451	0.06	hypothetical protein
Ben3g10984	0.06	Vegetative incompatibility protein HET-E-1
Ben3g6827	0.06	Uncharacterized transporter
Ben3g5561	0.06	hypothetical protein
Ben3g3020	0.06	hypothetical protein
Ben3g5549	0.06	Fruiting body protein SC7
Ben3g11444	0.06	hypothetical protein

Ben3g9363	0.06	hypothetical protein
Ben3g3652	0.06	Vegetative incompatibility protein HET-E-1
Ben3g10125	0.06	hypothetical protein
Ben3g7318	0.06	Lipase
Ben3g10839	0.06	hypothetical protein
Ben3g8419	0.06	O-methylsterigmatocystin oxidoreductase
Ben3g10770	0.06	hypothetical protein
Ben3g5504	0.06	hypothetical protein
Ben3g6437	0.06	ATP-dependent helicase homolog
Ben3g9914	0.06	hypothetical protein
Ben3g6398	0.06	hypothetical protein
Ben3g6776	0.06	Putative AC transposase
Ben3g11821	0.06	ATP-dependent helicase
Ben3g6444	0.06	hypothetical protein
Ben3g6661	0.06	Tyrosinase R2
Ben3g4121	0.06	Transmembrane protein 188
Ben3g9883	0.05	hypothetical protein
Ben3g5929	0.05	hypothetical protein
Ben3g3421	0.05	Chitin deacetylase
Ben3g7778	0.05	hypothetical protein
Ben3g11717	0.05	Putative 12-oxophytodienoate reductase protein 1
Ben3g6234	0.05	ATP-dependent DNA helicase Q-like 1
Ben3g12565	0.05	Pogo transposable element with ZNF domain
Ben3g4064	0.05	hypothetical protein
Ben3g5424	0.05	hypothetical protein
Ben3g7840	0.05	hypothetical protein
Ben3g9340	0.05	hypothetical protein
Ben3g11625	0.05	Probable RNA-directed DNA polymerase
Ben3g2203	0.05	hypothetical protein
Ben3g5409	0.05	hypothetical protein
Ben3g11019	0.05	hypothetical protein
Ben3g7051	0.05	Pathogenesis-related protein 5
Ben3g8585	0.05	hypothetical protein
Ben3g9499	0.05	3-hydroxyacyl-CoA dehydrogenase type-2
Ben3g3021	0.05	hypothetical protein
Ben3g3220	0.05	hypothetical protein
Ben3g11940	0.05	hypothetical protein
Ben3g8420	0.04	Glucan endo-1,6-beta-glucosidase B
Ben3g2874	0.04	hypothetical protein
Ben3g12065	0.04	Putative AC transposase
Ben3g12026	0.04	hypothetical protein
Ben3g10931	0.04	Vegetative incompatibility protein HET-E-1
Ben3g5976	0.04	hypothetical protein
Ben3g8119	0.04	Protein RTA1
Ben3g11659	0.04	hypothetical protein
Ben3g10891	0.04	hypothetical protein
Ben3g6137	0.04	hypothetical protein
Ben3g11783	0.04	hypothetical protein
Ben3g11384	0.04	hypothetical protein
Ben3g10914	0.04	hypothetical protein

Ben3g3221	0.04	hypothetical protein
Ben3g11514	0.04	hypothetical protein
Ben3g11628	0.04	hypothetical protein
Ben3g10567	0.04	hypothetical protein
Ben3g10312	0.04	78 kDa glucose-regulated protein
Ben3g3025	0.04	Endochitinase 1
Ben3g9966	0.04	hypothetical protein
Ben3g6669	0.04	Uncharacterized protein
Ben3g9166	0.04	hypothetical protein
Ben3g12074	0.04	hypothetical protein
Ben3g8941	0.04	hypothetical protein
Ben3g10613	0.04	Probable pectate lyase A
Ben3g7038	0.03	hypothetical protein
Ben3g10405	0.03	hypothetical protein
Ben3g8623	0.03	O-methylsterigmatocystin oxidoreductas
Ben3g10791	0.03	TPR repeat-containing protein
Ben3g8799	0.03	hypothetical protein
Ben3g11621	0.03	Sec1 family protein
Ben3g4065	0.03	hypothetical protein
Ben3g11791	0.03	hypothetical protein
Ben3g7784	0.03	Tyrosinase
Ben3g8660	0.03	hypothetical protein
Ben3g11018	0.03	hypothetical protein
Ben3g10788	0.03	hypothetical protein
Ben3g11856	0.03	hypothetical protein
Ben3g7603	0.03	Polygalacturonase
Ben3g5283	0.03	Probable rhamnogalacturonate lyase B
Ben3g12021	0.03	hypothetical protein
Ben3g8750	0.03	hypothetical protein
Ben3g1350	0.03	hypothetical protein
Ben3g10988	0.03	hypothetical protein
Ben3g11986	0.03	hypothetical protein
Ben3g7034	0.03	hypothetical protein
Ben3g9818	0.03	hypothetical protein
Ben3g7791	0.03	hypothetical protein
Ben3g10726	0.03	hypothetical protein
Ben3g12022	0.03	Putative uncharacterized oxidoreductase YDR541C
Ben3g11927	0.03	hypothetical protein
Ben3g12442	0.03	hypothetical protein
Ben3g11812	0.03	hypothetical protein
Ben3g11933	0.02	hypothetical protein
Ben3g4618	0.02	hypothetical protein
Ben3g11123	0.02	hypothetical protein
Ben3g4259	0.02	hypothetical protein
Ben3g12180	0.02	hypothetical protein
Ben3g1962	0.02	Sterigmatocystin biosynthesis P450 monooxygenase
Ben3g5334	0.02	Pectin lyase
Ben3g6354	0.02	Polygalacturonase
Ben3g9348	0.02	hypothetical protein
Ben3g6537	0.02	hypothetical protein

Ben3g6569	0.02	hypothetical protein
Ben3g6202	0.02	hypothetical protein
Ben3g9010	0.02	Microtubule-actin cross-linking factor 1
Ben3g11626	0.02	hypothetical protein
Ben3g11788	0.02	ATP-dependent DNA helicase PIF1
Ben3g11524	0.02	hypothetical protein
Ben3g11449	0.02	hypothetical protein
Ben3g7681	0.02	hypothetical protein
Ben3g11350	0.01	E3 ubiquitin-protein ligase
Ben3g11280	0.01	hypothetical protein
Ben3g11106	0.01	hypothetical protein
Ben3g9577	0.01	hypothetical protein
Ben3g7848	0.01	hypothetical protein
Ben3g4851	0.01	hypothetical protein
Ben3g9461	0.01	hypothetical protein
Ben3g11064	0.01	hypothetical protein
Ben3g10976	0.01	hypothetical protein
Ben3g10971	0.01	hypothetical protein
Ben3g11464	0.01	hypothetical protein
Ben3g7589	0.01	hypothetical protein
Ben3g9521	0.01	hypothetical protein
Ben3g8851	0.01	hypothetical protein
Ben3g8511	0.01	Peptidyl-prolyl isomerase H
Ben3g11099	0.01	hypothetical protein

hypothetical proteins
putative transcriptional regulators
involved in protein degradation
involved in cell wall degradation
lectin domain containing proteins
specifically mentioned proteins
nitrogen assimilation

Supplemental Table 2: List of genes transcribed at both dpi with plant contact

SeqName	RPKM sum	Description
Ben3g2563	127.18	Sterol 14-demethylase
Ben3g7568	69.23	Homeobox domain protein
Ben3g4553	61.04	C2H2-type zinc-finger protein
Ben3g9965	52.02	hypothetical protein
Ben3g8431	42.49	Aminopeptidase
Ben3g10264	38.35	Putative endoglucanase type K
Ben3g6837	33.99	Cytochrome b2, mitochondrial
Ben3g5920	28.24	Sulfite efflux pump SSU1
Ben3g8979	25.69	Putative transmembrane protein
Ben3g12402	23.73	hypothetical protein
Ben3g8486	17.79	hypothetical protein
Ben3g8133	16.78	hypothetical protein
Ben3g2756	16.66	hypothetical protein
Ben3g8475	14.08	Endoglucanase type K
Ben3g10881	11.70	Ubiquitin
Ben3g7223	10.91	Pectin lyase, pelD
Ben3g9518	8.99	hypothetical protein
Ben3g2757	8.70	L-ornithine 5-monooxygenase
Ben3g7035	8.40	hypothetical protein
Ben3g9172	5.46	Hydrolase
Ben3g2601	4.54	Benzoate 4-monooxygenase
Ben3g6323	4.05	Endochitinase 1
Ben3g11520	4.01	hypothetical protein
Ben3g11935	3.23	Protein TOXD
Ben3g7036	2.70	High-affinity glucose transporter
Ben3g11537	2.66	hypothetical protein
Ben3g3950	2.25	Aquaporin-1
Ben3g11298	1.48	hypothetical protein
Ben3g10096	0.97	hypothetical protein

hypothetical proteins
putative transcriptional regulators
involved in protein degradation
involved in cell wall degradation
lectin domain containing proteins
specifically mentioned proteins
nitrogen assimilation

Supplemental Table 3: List of genes transcribed exclusively at 3 dpi with plant contact

SeqName	RPKM	Description
Ben3g2370	29.60	hypothetical protein
Ben3g1991	11.96	hypothetical protein
Ben3g10121	8.11	Peptidase inhibitor i9
Ben3g2370	6.40	Glutathione S-transferase-like protein, putative
Ben3g7381	5.56	hypothetical protein
Ben3g302	4.91	hypothetical protein
Ben3g7995	4.58	hypothetical protein
Ben3g11519	3.71	DDE family endonuclease
Ben3g9248	2.82	Putative 12-oxophytodienoate reductase-like protein 1
Ben3g9413	2.82	hypothetical protein
Ben3g2801	2.74	hypothetical protein
Ben3g7864	2.28	Jacalin-like lectin domain
Ben3g7068	1.81	Putative uncharacterized oxidoreductase
Ben3g6930	1.78	hypothetical protein
Ben3g7333	1.75	hypothetical protein
Ben3g3307	1.31	hypothetical protein
Ben3g11214	1.31	hypothetical protein
Ben3g12192	1.11	Glucosylceramidase
Ben3g12491	1.08	hypothetical protein
Ben3g12264	0.89	hypothetical protein
Ben3g5495	0.75	Oxalate decarboxylase
Ben3g6244	0.74	NADH:flavin oxidoreductase/12-oxophytodienoate reductase
Ben3g11299	0.72	hypothetical protein
Ben3g7494	0.67	hypothetical protein
Ben3g7567	0.58	Uncharacterized transporter
Ben3g12237	0.53	O-methylsterigmatocystin oxidoreductase
Ben3g11926	0.25	SWI/SNF-related matrix-associated actin-dependent regulator

hypothetical proteins
putative transcriptional regulators
involved in protein degradation
involved in cell wall degradation
lectin domain containing proteins
specifically mentioned proteins
nitrogen assimilation

Supplemental Table 4: List of genes transcribed exclusively at 8 dpi with plant contact

SeqName	RPKM	Description
Ben3g732	58.56	hypothetical protein
Ben3g3368	30.49	Probable pectate lyase E
Ben3g5383	12.61	Cellulose-growth-specific protein
Ben3g1717	10.09	6-phosphogluconolactonase
Ben3g4865	9.84	Probable pectate lyase E
Ben3g2852	9.41	Probable cutinase
Ben3g1457	8.64	Probable endo-1,3(4)-beta-glucanase
Ben3g9325	8.11	Bifunctional xylanase/deacetylase
Ben3g5299	7.63	Endo-beta-1,4-glucanase D
Ben3g8490	7.52	Cellulose-growth-specific protein
Ben3g4785	6.36	Lipase
Ben3g12473	6.34	Pectate lyase, putative
Ben3g4874	4.04	hypothetical protein
Ben3g4625	4.01	hypothetical protein
Ben3g7263	3.84	hypothetical protein
Ben3g5302	3.62	hypothetical protein
Ben3g10258	2.72	hypothetical protein
Ben3g3590	2.51	hypothetical protein
Ben3g9854	2.03	Extracellular metalloprotease
Ben3g11300	2.03	hypothetical protein
Ben3g12096	0.70	hypothetical protein

hypothetical proteins
putative transcriptional regulators
involved in protein degradation
involved in cell wall degradation
lectin domain containing proteins
specifically mentioned proteins
nitrogen assimilation

Supplemental Table 5: List of most highest transcribed sequences in mycelium without plant contac

SeqName	RPKM	Description
Ben3g9368	10341	hypothetical protein
Ben3g9573	8976	hypothetical protein
Ben3g9146	7960	h-lectin domain containing protein
Ben3g8806	7706	Thuringiensis toxin domain protein
Ben3g8352	7227	Anucleate primary sterigmata protein B, putative
Ben3g11931	4097	Thuringiensis toxin domain protein
Ben3g8800	3754	Thuringiensis toxin domain protein
Ben3g12158	3329	hypothetical protein
Ben3g6448	3309	hypothetical protein
Ben3g10825	2921	Transmembrane protein, putative
Ben3g924	2820	transmembrane protein, putative
Ben3g7115	2760	Septal pore cap protein SPC18
Ben3g8592	2641	hypothetical protein
Ben3g1637	2242	Phosphatidylserine decarboxylase proenzyme, putative
Ben3g7899	2072	hypothetical protein
Ben3g7031	2071	Hemerythrin HHE cation-binding domain protein
Ben3g1558	1868	Ubiquitin-conjugating enzyme E2
Ben3g66	1794	Translation elongation factor eEF3
Ben3g2326	1706	hypothetical protein
Ben3g5323	1640	hypothetical protein
Ben3g1995	1633	Aspartic peptidase
Ben3g675	1511	hypothetical protein
Ben3g3829	1461	Peptidase inhibitor clitocypin protein
Ben3g2970	1385	Subtilisin-like serine protease
Ben3g5304	1383	Subtilisin-like serine protease family protein
Ben3g10057	1334	Ricin-type beta-trefoil lectin domain-containing protein
Ben3g644	1318	FliC domain protein
Ben3g8804	1314	D-mannose-binding b-lectin
Ben3g2010	1246	Nucleoside diphosphate kinase
Ben3g9350	1236	Ricin superfamily
Ben3g6583	1210	putative transmembrane protein
Ben3g5207	1207	Peptidyl-prolyl cis-trans isomerase
Ben3g6614	1198	Hemopexin domain protein
Ben3g7555	1192	Lipase class 3
Ben3g8928	1161	Phosphatidylserine decarboxylase
Ben3g8869	1155	Ricin-type beta-trefoil lectin domain protein
Ben3g683	1065	AN1-type zinc finger protein

hypothetical proteins
putative transcriptional regulators
involved in protein degradation
involved in cell wall degradation
lectin domain containing proteins
specifically mentioned proteins
nitrogen assimilation
bold SeqName are in common in all three tabs

:t

Supplemental Table 6: List of most highest transcribed sequences at 3 dpi

SeqName	RPKM	Description
Ben3g6614	4814	Hemopexin domain protein
Ben3g8806	4161	Thuringiensis toxin domain protein
Ben3g7115	3721	Septal pore cap protein SPC18
Ben3g6583	3176	putative transmembrane protein
Ben3g9146	3078	h-type lectin domain-containing protein
Ben3g8452	2910	Lipoic acid synthetase
Ben3g5323	2577	hypothetical protein
Ben3g11931	2410	Thuringiensis toxin domain protein
Ben3g4641	2357	60S ribosomal protein L30
Ben3g6448	2289	hypothetical protein
Ben3g12158	2018	hypothetical protein
Ben3g4642	1898	hypothetical protein
Ben3g2326	1795	hypothetical protein
Ben3g4888	1733	Nuclear ribonucleoprotein G RNA recognition motif protein
Ben3g8317	1719	Heat shock protein
Ben3g9573	1466	hypothetical protein
Ben3g9144	1323	Glycoside hydrolase family 61 protein
Ben3g5207	1238	Peptidyl-prolyl cis-trans isomerase
Ben3g675	1168	hypothetical protein
Ben3g7899	1109	hypothetical protein
Ben3g8869	1097	Ricin-type beta-trefoil lectin domain protein
Ben3g1558	1087	Ubiquitin-conjugating enzyme E2
Ben3g3831	1025	Histone H2A
Ben3g8800	1000	Thuringiensis toxin domain protein

hypothetical proteins
putative transcriptional regulators
involved in protein degradation
involved in cell wall degradation
lectin domain containing proteins
specifically mentioned proteins
nitrogen assimilation
bold SeqName are in common in all three tabs

Supplemental Table 7: List of most highest transcribed sequences at 8 dpi

SeqName	RPKM	Description
Ben3g9573	4869	hypothetical protein
Ben3g7115	3303	Septal pore cap protein SPC18
Ben3g8806	2256	Thuringiensis toxin domain protein
Ben3g9146	1734	h-type lectin domain-containing protein
Ben3g66	1688	Translation elongation factor eEF3
Ben3g683	1685	AN1-type zinc finger protein
Ben3g666	1540	Eukaryotic translation initiation factor SUI1
Ben3g9337	1511	NADH-ubiquinone reductase complex 1 MLRQ subunit
Ben3g924	1442	Transmembrane protein, putative
Ben3g6614	1440	Hemopexin domain protein
Ben3g4773	1426	Transmembrane protein, putative
Ben3g8317	1398	Heat shock protein
Ben3g5323	1393	hypothetical protein
Ben3g8352	1372	Anucleate primary sterigmata protein B, putative
Ben3g11931	1356	Thuringiensis toxin domain protein
Ben3g4888	1235	Nuclear ribonucleoprotein G RNA recognition motif protein
Ben3g1558	1223	Ubiquitin-conjugating enzyme E2
Ben3g9368	1210	hypothetical protein
Ben3g5207	1182	Peptidyl-prolyl cis-trans isomerase
Ben3g9350	1147	Ricin superfamily
Ben3g6448	1126	hypothetical protein
Ben3g2326	1102	hypothetical protein
Ben3g5768	1048	bZIP transcription factor domain-containing protein
Ben3g2448	1039	40S ribosomal protein S12
Ben3g9151	1011	Glutathione S-transferase

hypothetical proteins
putative transcriptional regulators
involved in protein degradation
involved in cell wall degradation
lectin domain containing proteins
specifically mentioned proteins
nitrogen assimilation
bold SeqName are in common in all three tabs

Supplemental Table 8: List of genes differentially transcribed at 3 dpi

SeqName	BaseMean	log2FoldChange	Description
Ben3g6247	256	10.12	Protein RTA1
Ben3g4553	64	9.97	C2H2-type zinc-finger protein
Ben3g1929	198	9.88	putative extracellular dioxygenase
Ben3g6837	83	9.52	FMN-dependent dehydrogenase
Ben3g2070	1102	9.00	extracellular metalloprotease
Ben3g251	1157	8.99	haloacid dehalogenase-like hydrolase, phosphatase
Ben3g3530	451	8.93	pectate lyase, putative
Ben3g10121	18	8.80	aqualysin-1, protease, endopeptidase
Ben3g6269	171	8.57	Guanyl-specific ribonuclease F1
Ben3g8007	135	8.51	polysaccharide lyase family 1 protein
Ben3g2563	65	8.29	cytochrome P450 family 6 protein
Ben3g8133	19	8.07	hypothetical protein
Ben3g818	74	8.06	transmembrane protein, putative
Ben3g6767	571	7.93	solute symporter family transporter
Ben3g4369	92	7.78	APC amino acid permease
Ben3g7678	1866	7.70	cuticle-degrading protease
Ben3g11520	8	7.64	SNF2 family amino-terminal protein
Ben3g5368	80	7.64	alpha/beta hydrolase fold protein
Ben3g9957	132	7.58	GMC oxidoreductase
Ben3g9507	185	7.49	peptidyl-Lys metalloendopeptidase
Ben3g4748	194	7.25	serine protease
Ben3g11537	7	7.18	hypothetical protein
Ben3g1242	27	7.13	Probable cutinase
Ben3g11079	868	7.03	Cuticle-degrading protease
Ben3g2370	7	7.01	glutathione S-transferase-like protein, putative
Ben3g2705	113	7.00	sugar porter (SP) family MFS transporter
Ben3g7568	14	6.98	homeobox domain protein
Ben3g6359	966	6.97	nitrate reductase (NADPH)
Ben3g8979	21	6.94	putative transmembrane protein
Ben3g8431	34	6.88	aminopeptidase
Ben3g4845	65	6.85	transmembrane protein, putative
Ben3g10213	44	6.83	Helix loop helix DNA-binding domain protein, hypothetical protein
Ben3g9248	10	6.83	NADH:flavin oxidoreductase/NADH oxidase
Ben3g935	796	6.81	peptidyl-Lys metalloendopeptidase
Ben3g2639	89	6.80	Aromatic peroxygenase
Ben3g11519	9	6.77	DDE family endonuclease
Ben3g189	23	6.74	lysine N-acyltransferase, putative
Ben3g6361	764	6.73	nitrite reductase (NAD(P)H) large subunit
Ben3g9506	941	6.72	deuterolysin metalloprotease (M35) family containing protein
Ben3g5920	5	6.71	C4-dicarboxylate transporter/malic acid transporter
Ben3g9480	101	6.65	hypothetical protein
Ben3g6147	1122	6.59	ammonium transporter
Ben3g7380	28	6.54	hypothetical protein
Ben3g2501	15	6.49	hypothetical protein
Ben3g710	384	6.48	peptidyl-Lys metalloendopeptidase
Ben3g3286	37	6.45	alpha/beta hydrolase fold protein
Ben3g9172	11	6.42	amidohydrolase
Ben3g2720	14	6.42	5'/3'-nucleotidase SurE family protein
Ben3g2601	5	6.37	Benzoate 4-monooxygenase
Ben3g8452	901	6.27	lipoic acid synthetase
Ben3g7068	5	6.26	NADPH-dependent methylglyoxal reductase GRE2
Ben3g8250	99	6.26	peptidase S8/S53 subtilisin kexin sedolisin
Ben3g8251	37	6.26	Cuticle-degrading protease
Ben3g12402	4	6.24	hypothetical protein
Ben3g54	13	6.23	NADPH-dependent aldehyde reductase
Ben3g5192	75	6.17	concanamycin induced protein C CipC1 protein, putative
Ben3g2881	92	6.13	MFS general substrate transporter
Ben3g5460	19	6.13	hypothetical protein
Ben3g2757	13	6.11	L-ornithine 5-monooxygenase
Ben3g9489	94	6.07	cytochrome P450 family oxidoreductase
Ben3g9675	127	6.05	cytochrome P450 family monooxygenase
Ben3g2090	37	6.03	hypothetical protein, putative transmembrane protein

Ben3g7494	2	6.01	hypothetical protein
Ben3g2096	129	6.01	Uncharacterized MFS-type transporter
Ben3g5495	3	5.94	oxalate decarboxylase
Ben3g302	6	5.94	galactose-proton symport protein, putative
Ben3g2801	3	5.93	glutathione transferase, putative
Ben3g12073	30	5.79	DDE family endonuclease
Ben3g9112	14	5.79	FMN-dependent dehydrogenase
Ben3g11214	4	5.78	hypothetical protein
Ben3g6930	5	5.77	protocatechuate-dioxygenase beta subunit protein, putative
Ben3g7223	5	5.76	pectin lyase
Ben3g1779	91	5.71	MFS multidrug transporter, putative
Ben3g2447	62	5.69	carbohydrate-binding domain protein, putative
Ben3g10325	117	5.66	O-methylsterigmatocystin oxidoreductase
Ben3g8740	18	5.61	glycoside hydrolase family 61 protein
Ben3g6715	2601	5.59	extracellular metalloproteinase MEP
Ben3g2997	29	5.53	carboxyphosphonoenolpyruvate phosphonomutase, putative
Ben3g7870	30	5.50	glycoside hydrolase family 3 protein
Ben3g4404	447	5.49	Ammonium transporter MEP3
Ben3g12491	4	5.45	Phosphatidylinositol 3-kinase VPS34
Ben3g11299	3	5.44	Cell surface glycoprotein 1
Ben3g2071	440	5.44	extracellular metalloprotease
Ben3g3871	249	5.43	nonribosomal peptide synthetase
Ben3g46	101	5.41	Linear gramicidin synthase subunit D
Ben3g8112	8	5.39	isoflavone reductase-like protein
Ben3g9140	881	5.35	extracellular metalloproteinase MEP
Ben3g8213	26	5.33	transmembrane protein, putative
Ben3g7019	207	5.32	MFS sugar transporter, putative
Ben3g7687	266	5.31	bacterial leucyl aminopeptidase
Ben3g2619	5	5.30	peroxidase, putative
Ben3g4361	233	5.26	acetamidase/formamidase
Ben3g1402	1973	5.23	1,4-alpha-glucan branching enzyme
Ben3g4201	88	5.22	putative pheromone-regulated membrane protein
Ben3g4243	128	5.21	endo-polygalacturonase PG1
Ben3g8595	248	5.15	hypothetical protein
Ben3g3800	12	5.15	glycoside hydrolase family 18 protein
Ben3g4688	31	5.15	hypothetical protein, peptidase family S41 domain
Ben3g7612	120	5.14	putative gEgh 16 protein
Ben3g79	326	5.06	pleiotropic drug resistance ABC transporter
Ben3g2821	42	4.95	citrate synthase
Ben3g11935	4	4.94	zinc-binding oxidoreductase ToxD, putative
Ben3g8399	223	4.94	carboxypeptidase a2
Ben3g12427	1	4.94	hypothetical protein
Ben3g11267	1	4.92	Peptidyl-Lys metalloendopeptidase
Ben3g12192	2	4.91	glucosylceramidase
Ben3g9120	65	4.90	guanyl-specific ribonuclease F1
Ben3g6910	60	4.89	putative iron reductase
Ben3g7381	3	4.89	hypothetical protein
Ben3g4242	23	4.84	endo-polygalacturonase PG1
Ben3g4382	13	4.82	4-hydroxybenzoate polyprenyltransferase
Ben3g3307	4	4.81	hypothetical protein
Ben3g7920	45	4.77	putative transmembrane protein
Ben3g5375	16	4.77	AMP-binding enzyme
Ben3g9145	13	4.74	NADH:flavin oxidoreductase/NADH oxidase
Ben3g7995	3	4.73	4-oxalocrotonate tautomerase
Ben3g6456	2	4.73	hypothetical protein
Ben3g7073	50	4.73	feruloyl esterase B
Ben3g3249	43	4.71	cysteine-rich secretory family protein
Ben3g10009	16	4.71	Peptidase family S41
Ben3g7378	22	4.68	lipase from carbohydrate esterase family CE10 protein, putative
Ben3g1991	8	4.67	hypothetical protein
Ben3g11213	1	4.66	DDE family endonuclease
Ben3g6660	4	4.66	tyrosinase tyrosinase: common central domain protein
Ben3g3886	44	4.61	phosphoglycerate mutase family protein
Ben3g2524	8	4.57	Putative cytochrome P450 CYP13A3
Ben3g7036	4	4.54	sugar transporter

Ben3g6155	70	4.53	putative quinate permease
Ben3g11298	2	4.53	hypothetical protein
Ben3g7020	87	4.52	mandelate racemase/muconate lactonizing enzyme family protein
Ben3g4462	109	4.52	hypothetical protein
Ben3g10343	5	4.51	Zinc-type alcohol dehydrogenase-like protein C1773.06c
Ben3g9518	4	4.51	hypothetical protein
Ben3g11957	11	4.49	Putative AC transposase
Ben3g7775	59	4.48	xanthine/uracil permease
Ben3g2756	1	4.47	L-ornithine 5-monooxygenase
Ben3g7864	4	4.46	jacalin-like lectin domain protein, putative
Ben3g8649	7	4.45	glycoside hydrolase family 61 protein
Ben3g2953	79	4.44	hypothetical protein
Ben3g6088	134	4.43	serine carboxypeptidase
Ben3g8430	460	4.41	aminopeptidase
Ben3g8483	1	4.41	deuterolysin metalloprotease (M35) family containing protein
Ben3g8400	13	4.40	glycoside hydrolase family 5 protein
Ben3g11054	344	4.40	glycoside hydrolase family 61 protein
Ben3g6898	75	4.39	glycoside hydrolase family 3 protein
Ben3g6170	4	4.38	hypothetical protein
Ben3g9268	6	4.35	cytochrome P450 family protein
Ben3g8325	28	4.33	putative peptide transporter ptr2
Ben3g3400	4	4.31	phosphotransferase enzyme family protein
Ben3g6360	1410	4.31	putative high affinity nitrate transporter
Ben3g10660	754	4.30	glutamate dehydrogenase (NADP+)
Ben3g9018	15	4.30	S-adenosyl-L-methionine-dependent methyltransferase
Ben3g5152	62	4.28	3-ketoacyl-(acyl-carrier) reductase
Ben3g908	51	4.28	MFS MFS-1 protein
Ben3g7842	288	4.27	NADH:flavin oxidoreductase
Ben3g7787	2	4.27	chitin-binding domain protein
Ben3g12315	46	4.25	hypothetical protein
Ben3g6321	54	4.23	tyrosinase tyrosinase: common central domain protein
Ben3g9413	2	4.22	hypothetical protein
Ben3g1351	5	4.20	Pectinesterase
Ben3g9897	189	4.19	aminopeptidase Y
Ben3g1751	275	4.16	tripeptidyl-peptidase I
Ben3g6249	24	4.16	RTA1-domain protein
Ben3g1829	5	4.15	COP8 protein
Ben3g5921	63	4.14	sterol 24-C-methyltransferase Erg6
Ben3g8907	66	4.14	proline dehydrogenase
Ben3g8715	13	4.13	xyloglucan-specific endo-beta-1,4-glucanase A
Ben3g537	84	4.12	DUF3129 family protein
Ben3g8237	124	4.10	phosphatidylcholine-hydrolyzing phospholipase C
Ben3g7530	27	4.09	cytochrome P450, family 51 (sterol 14-demethylase)
Ben3g8474	2	4.08	hypothetical protein
Ben3g7252	210	4.07	xanthine/uracil permease C887, putative
Ben3g8536	8	4.04	hypothetical protein
Ben3g9387	982	4.04	alpha/beta hydrolase family containing protein
Ben3g9426	785	4.04	deuterolysin metalloprotease (M35) family containing protein
Ben3g6322	72	4.02	tyrosinase tyrosinase: common central domain protein
Ben3g11549	45	4.02	hypothetical protein
Ben3g11926	2	4.02	SNF2 family amino-terminal protein
Ben3g3283	60	4.01	high-affinity nicotinic acid transporter
Ben3g3282	37	3.99	high-affinity nicotinic acid transporter
Ben3g4921	42	3.99	oxalate decarboxylase
Ben3g11866	3	3.98	hypothetical protein
Ben3g7619	114	3.95	FAD/NAD(P)-binding domain protein
Ben3g5516	7	3.92	Monocarboxylate transporter 12
Ben3g5353	8	3.92	putative cyclin-dependent kinase 9
Ben3g10912	1	3.92	hypothetical protein
Ben3g11898	1	3.89	hypothetical protein
Ben3g2731	654	3.89	inorganic phosphate transporter
Ben3g9873	11	3.88	hypothetical protein
Ben3g9674	1	3.87	phenolic acid decarboxylase
Ben3g6323	5	3.87	glycoside hydrolase family 18
Ben3g186	8	3.85	enoyl-CoA hydratase/isomerase

Ben3g4483	23	3.84	WD domain, G-beta repeat protein
Ben3g3048	5	3.83	GDSL-like lipase/acylhydrolase
Ben3g10311	6	3.83	hypothetical protein
Ben3g6724	31	3.82	3-ketoacyl-(acyl-carrier) reductase
Ben3g3872	42	3.82	enoyl-CoA hydratase
Ben3g12446	8	3.81	C2H2-type zinc-finger protein
Ben3g5094	50	3.80	acetyltransferase (GNAT) family containing protein
Ben3g7084	108	3.78	iron permease FTR1
Ben3g4104	5	3.78	hypothetical protein
Ben3g2880	129	3.77	MFS general substrate transporter
Ben3g4735	69	3.76	dihydrodipicolinate synthetase family protein
Ben3g7742	21	3.75	hypothetical protein
Ben3g3298	9	3.74	Dynactin subunit 1
Ben3g12106	1	3.74	hypothetical protein
Ben3g2904	3	3.73	Zinc-type alcohol dehydrogenase-like protein
Ben3g7173	47	3.72	cuticle-degrading protease, putative
Ben3g982	30	3.72	hypothetical protein
Ben3g4060	162	3.72	glycoside hydrolase family 61 protein
Ben3g12534	4	3.70	hypothetical protein
Ben3g8525	41	3.70	glycosyltransferase family 8 protein
Ben3g5801	333	3.70	MFS general substrate transporter
Ben3g3267	63	3.69	epoxide hydrolase
Ben3g4197	123	3.68	S1/P1 nuclease
Ben3g9386	24	3.67	alpha/beta hydrolase family containing protein
Ben3g7009	91	3.67	phosphoadenosine phosphosulfate reductase
Ben3g1686	483	3.66	Thiamine thiazole synthase
Ben3g2177	246	3.66	Uncharacterized permease
Ben3g2879	250	3.65	diphthine synthase
Ben3g12360	1	3.64	Sterol 3-beta-glucosyltransferase
Ben3g1988	39	3.63	putative MFS multidrug resistance transporter
Ben3g10169	10	3.61	hypothetical protein
Ben3g7035	2	3.61	sugar transporter
Ben3g6257	1	3.61	enoyl-(acyl carrier) reductase
Ben3g1687	949	3.61	Thiamine thiazole synthase
Ben3g2749	132	3.60	aminopeptidase Y, putative
Ben3g12016	2	3.59	reverse transcriptase
Ben3g8185	15	3.58	transmembrane protein, putative
Ben3g7563	39	3.58	Phosphatidylinositol 3-kinase VPS34
Ben3g1322	64	3.56	hypothetical protein
Ben3g2996	41	3.55	oxaloacetate acetylhydrolase
Ben3g7024	90	3.55	transmembrane protein, putative
Ben3g12151	75	3.55	glycoside hydrolase family protein
Ben3g4799	9	3.54	transmembrane protein, putative
Ben3g1282	7	3.54	O-methylsterigmatocystin oxidoreductase
Ben3g2443	41	3.54	xylitol dehydrogenase
Ben3g5782	238	3.54	transmembrane protein, putative
Ben3g815	1	3.54	cytidine and deoxycytidylate deaminase zinc-binding region protein
Ben3g8927	3	3.52	putative transmembrane protein
Ben3g8966	2	3.52	hypothetical protein
Ben3g7083	3	3.51	hypothetical protein
Ben3g3391	20	3.50	double-stranded RNA-binding motif protein
Ben3g9854	1	3.47	Extracellular metalloprotease 1
Ben3g8294	15	3.47	macrofage activating glycoprotein, putative
Ben3g6244	2	3.46	NADH flavin oxidoreductase/NADH oxidase
Ben3g4759	130	3.43	glucan 1,3-beta-glucosidase
Ben3g3051	15	3.43	laccase, multicopper oxidase, benzenediol:oxygen oxidorectuctase
Ben3g11225	2	3.43	hypothetical protein
Ben3g3299	3	3.42	Protein NYNRIN
Ben3g9775	15	3.42	drug:H+ antiporter
Ben3g1281	35	3.41	alpha/beta hydrolase family containing protein
Ben3g2183	52	3.41	Alpha-xylosidase
Ben3g6829	62	3.40	alpha-L-rhamnosidase, partial
Ben3g12237	2	3.39	O-methylsterigmatocystin oxidoreductase
Ben3g3519	52	3.39	3-methyl-2-oxobutanoate hydroxymethyltransferase
Ben3g11597	1	3.39	hypothetical protein

Ben3g7809	6	3.36	hypothetical protein
Ben3g8486	11	3.36	hypothetical protein
Ben3g9850	3	3.35	hypothetical protein
Ben3g10860	3	3.34	hypothetical protein
Ben3g10783	256	3.34	glycoside hydrolase family protein
Ben3g1699	3	3.33	putative serine-rich protein C13G6,10c
Ben3g9388	712	3.33	thiamine biosynthesis protein
Ben3g5367	21	3.33	UDP-glycosyltransferase 74C1
Ben3g2285	112	3.33	hypothetical protein
Ben3g9725	50	3.32	isoamyl alcohol oxidase, putative
Ben3g10096	2	3.31	hypothetical protein
Ben3g4628	17	3.31	F-box-like domain protein
Ben3g10113	826	3.31	pyridoxal-dependent decarboxylase domain protein
Ben3g4703	1	3.30	Probable endo-beta-1,4-glucanase
Ben3g8017	4	3.30	retrotransposon gag protein
Ben3g2816	85	3.30	hypothetical protein
Ben3g7364	35	3.30	glycoside hydrolase family 62 protein
Ben3g12004	17	3.30	Retrovirus-related Pol polyprotein from transposon TNT
Ben3g3503	6	3.29	N-acyl homoserine lactonase AttM
Ben3g5103	103	3.28	glycoside hydrolase family 61 protein
Ben3g3347	80	3.28	tyrosinase tyrosinase: common central domain protein
Ben3g9490	56	3.26	cytochrome P450 family oxidoreductase
Ben3g9593	476	3.26	sugar porter (SP) family MFS transporter
Ben3g305	55	3.24	diacylglycerol kinase catalytic domain protein
Ben3g4697	667	3.24	hypothetical protein
Ben3g7638	26	3.23	serine carboxypeptidase
Ben3g858	7	3.23	peptidoglycan-binding domain 1 protein, putative
Ben3g9767	2	3.22	Phosphatidylinositol 3-kinase VPS34
Ben3g10232	273	3.22	Tripeptidyl-peptidase
Ben3g4123	23	3.21	Alpha-1,3-mannosyltransferase
Ben3g70	77	3.19	hypothetical protein
Ben3g2924	148	3.18	Sexual differentiation process protein isp4
Ben3g3950	2	3.18	major intrinsic protein (MIP) family transporter
Ben3g10114	260	3.18	pyridoxal-dependent decarboxylase domain protein
Ben3g2858	192	3.17	FAD-dependent oxidoreductase, putative
Ben3g7067	17	3.16	NADPH-dependent methylglyoxal reductase GRE2
Ben3g8739	6	3.14	meiotic recombination protein DMC1, related protein
Ben3g8596	287	3.14	extracellular elastinolytic metalloproteinase
Ben3g776	6	3.14	rare lipoprotein A-like double-psi beta-barrel protein
Ben3g3312	40	3.14	laccase, multicopper oxidase, benzenediol:oxygen oxidorectuctase
Ben3g2800	1	3.13	hypothetical protein
Ben3g8922	63	3.13	nonribosomal peptide synthetase, putative
Ben3g10404	3	3.12	Zinc-type alcohol dehydrogenase-like protein
Ben3g9280	77	3.12	extracellular elastinolytic metalloproteinase
Ben3g8469	43	3.12	transmembrane protein, putative
Ben3g7230	2	3.11	alpha/beta hydrolase fold protein
Ben3g5101	17	3.11	hypothetical protein
Ben3g2143	110	3.10	Esterase
Ben3g5039	9	3.10	procyclic acidic repetitive domain-containing protein
Ben3g62	119	3.10	ABC transporter, Pdr11p
Ben3g5102	40	3.09	glycoside hydrolase family 61 protein
Ben3g4881	3	3.09	pectate lyase, putative
Ben3g5936	2	3.08	glycoside hydrolase family 18 protein
Ben3g11747	2	3.08	hypothetical protein
Ben3g9502	54	3.07	deuterolysin metalloprotease (M35) family containing protein
Ben3g12015	2	3.07	Maltodextrin phosphorylase [Thermococcus litoralis DSM 5473]
Ben3g7759	4	3.05	peptidase inhibitor clitocypin protein
Ben3g362	430	3.05	MFS sugar transporter
Ben3g8008	8	3.05	4-oxalocrotonate tautomerase
Ben3g2707	139	3.05	serine carboxypeptidase
Ben3g8682	671	3.03	transmembrane protein, putative
Ben3g202	275	3.03	serine carboxypeptidase
Ben3g1654	657	3.03	Bacterial leucyl aminopeptidase
Ben3g7552	10	3.02	glycoside hydrolase family 1 protein
Ben3g10189	3	3.02	hypothetical protein

Ben3g2341	164	3.01	hypothetical protein
Ben3g3486	422	3.01	NAD(P)H-dependent D-xylose reductase xyl1, putative
Ben3g3123	29	2.99	3-hydroxyanthranilate 3,4-dioxygenase
Ben3g10227	95	2.98	tyrosinase tyrosinase: common central domain protein
Ben3g11831	163	2.96	Uncharacterized MFS-type transporter C409.08
Ben3g5974	15	2.96	hypothetical protein
Ben3g4088	661	2.95	glycogen(starch) synthase
Ben3g4656	14	2.95	cytochrome P450 family protein
Ben3g8998	1	2.95	carrier protein
Ben3g3571	3	2.93	endo-polygalacturonase, partial
Ben3g5949	4	2.93	hypothetical protein
Ben3g4274	52	2.92	hypothetical protein
Ben3g2539	3	2.92	NADP-dependent alcohol dehydrogenase 6
Ben3g11300	1	2.92	hypothetical protein
Ben3g1179	35	2.91	alkaline phosphatase, putative
Ben3g3659	312	2.91	L-serine dehydratase
Ben3g8673	1268	2.90	genomic scaffold, msy-sf-12 protein
Ben3g11003	15	2.87	Rhamnogalacturonan acetylesterase RhgT
Ben3g5006	78	2.85	sulfate adenylyltransferase
Ben3g1127	1343	2.85	trehalose 6-phosphate phosphatase
Ben3g8824	158	2.85	trehalose utilization protein
Ben3g4546	9	2.84	RNA recognition motif 3 in fungal Mei2-like protein
Ben3g2663	150	2.83	amino acid transporter
Ben3g9122	1000	2.83	Putative peroxiredoxin
Ben3g7326	174	2.82	delta(24(24(1)))-sterol reductase
Ben3g3014	9	2.82	F-box-like domain protein
Ben3g1833	1	2.82	winged helix turn helix protein
Ben3g8683	406	2.81	transmembrane protein, putative
Ben3g9113	2	2.81	FMN-dependent dehydrogenase
Ben3g4288	52	2.80	vacuole effluxer Atg22-like protein
Ben3g5072	392	2.80	malate dehydrogenase (oxaloacetate-decarboxylating)
Ben3g8023	1	2.80	C-14 sterol reductase ERG24
Ben3g2647	15	2.79	trimethyllysine dioxygenase
Ben3g6920	6	2.79	nucleoside diphosphate kinase
Ben3g7540	25	2.79	glycoside hydrolase family 61 protein
Ben3g12329	4	2.79	hypothetical protein
Ben3g5468	299	2.78	chitin-binding domain protein
Ben3g11002	1	2.78	Retrovirus-related Pol polyprotein from transposon
Ben3g9393	219	2.77	MFS sugar transporter, putative
Ben3g5424	1	2.77	hypothetical protein
Ben3g4813	171	2.76	hypothetical protein
Ben3g2972	20	2.76	aldo/keto reductase family protein
Ben3g7116	16	2.74	carboxylesterase
Ben3g8233	2	2.74	hypothetical protein
Ben3g4337	69	2.74	Protein priA
Ben3g8679	244	2.73	transmembrane protein, putative
Ben3g8470	29	2.73	transmembrane protein, putative
Ben3g9325	3	2.73	carbohydrate esterase family 4 protein
Ben3g4545	28	2.73	RNA recognition motif 3 in fungal Mei2-like protein
Ben3g3285	1	2.72	alpha/beta hydrolase fold protein
Ben3g5851	194	2.72	glycoside hydrolase family 3 protein
Ben3g4641	1639	2.72	NACHT domain protein
Ben3g3951	87	2.72	major intrinsic protein (MIP) family transporter
Ben3g1256	17	2.71	pectin lyase, putative
Ben3g8374	143	2.70	3-ketoacyl-(acyl-carrier) reductase
Ben3g3119	48	2.70	alpha-L-arabinofuranosidase
Ben3g3853	66	2.70	myo-inositol oxygenase
Ben3g732	1	2.70	hypothetical protein
Ben3g9551	9	2.69	aspartyl protease
Ben3g9229	41	2.69	3-beta hydroxysteroid dehydrogenase/isomerase family protein
Ben3g190	15	2.68	Siderophore iron transporter
Ben3g5386	2	2.68	hypothetical protein
Ben3g8538	118	2.68	4-nitrophenyl phosphatase
Ben3g6417	17	2.68	arabinogalactan endo-1,4-beta-galactosidase
Ben3g6444	1	2.67	transmembrane protein, putative

Ben3g4929	606	2.65	glycoside hydrolase family 63 protein
Ben3g1421	8	2.65	hypothetical protein
Ben3g6043	5	2.65	transmembrane protein, putative
Ben3g10264	2	2.65	glycoside hydrolase family 45 protein
Ben3g8621	34	2.65	allergen protein
Ben3g196	67	2.64	kynurenine 3-monooxygenase
Ben3g7836	72	2.64	glycoside hydrolase family 27 protein
Ben3g3435	2	2.63	Pol polyprotein/retrotransposon, putative
Ben3g4330	51	2.62	putative ethanolamine utilization protein (EutQ)
Ben3g4176	2	2.62	DUF3294 family protein
Ben3g6886	26	2.62	NADH flavin oxidoreductase/NADH oxidase
Ben3g6713	9	2.62	extracellular metalloproteinase MEP
Ben3g1658	66	2.62	Probable endo-1,3(4)-beta-glucanase
Ben3g9670	181	2.61	Minor extracellular protease vpr
Ben3g5836	49	2.61	C2H2 domain-containing protein
Ben3g3564	77	2.60	proteasome (prosome, macropain) activator subunit 3 (pa28 gamma)
Ben3g12356	4	2.60	tyrosinase tyrosinase: common central domain protein
Ben3g3569	49	2.60	MUC1 extracellular alpha-1,4-glucan glucosidase-like protein, putative
Ben3g872	45	2.60	guanine nucleotide-binding protein alpha-4 subunit
Ben3g3308	2	2.60	glutathione S-transferase
Ben3g7766	51	2.59	glycoside hydrolase family 1 protein
Ben3g10751	146	2.59	O-acetylhomoserine (thiol)-lyase
Ben3g6077	15	2.58	GDSL lipase/acylhydrolase family protein
Ben3g6625	107	2.57	putative secreted protein
Ben3g12259	10	2.57	hypothetical protein
Ben3g6905	25	2.56	L-lysine 6-monooxygenase (NADPH-requiring) protein
Ben3g2309	65	2.56	hypothetical protein
Ben3g8768	175	2.56	urease
Ben3g6820	36	2.56	choline dehydrogenase
Ben3g10082	1	2.55	hypothetical protein
Ben3g8719	117	2.54	SDA1 domain protein
Ben3g7379	4	2.54	endo-polygalacturonase PG1
Ben3g7799	24	2.53	homeobox domain protein
Ben3g2390	49	2.53	aldo/keto reductase family protein
Ben3g4236	6	2.53	hypothetical protein
Ben3g12228	5	2.53	hypothetical protein
Ben3g3868	64	2.53	cell wall glycosyl hydrolase YteR
Ben3g7287	17	2.52	DUF4598 domain protein
Ben3g6020	1	2.52	salicylate 1-monooxygenase
Ben3g4370	171	2.51	putative amino-acid permease PB24D3,02c
Ben3g5198	497	2.51	thioredoxin reductase (NADPH)
Ben3g5111	25	2.51	UPF0743 protein
Ben3g7510	35	2.50	zinc-binding dehydrogenase
Ben3g1717	1	2.50	lactonase, 7-bladed beta-propeller
Ben3g11806	1	2.49	ATP-dependent DNA helicase PIF1
Ben3g2713	160	2.49	membrane protein, putative
Ben3g7735	141	2.49	peptidase family s41 domain protein
Ben3g10040	0	2.48	4-oxalocrotonate tautomerase
Ben3g7996	0	2.48	HAT family dimerization protein
Ben3g8565	0	2.48	peptidyl-lys metalloendopeptidase
Ben3g9272	17	2.48	DUF1349 family protein
Ben3g7110	46	2.47	glycoside hydrolase family 13 protein
Ben3g6455	0	2.46	hypothetical protein
Ben3g5465	973	2.46	zinc containing alcohol dehydrogenase, putative
Ben3g1457	1	2.46	Family 16 endo-1,3(4)-beta-glucanase from glycoside hydrolase
Ben3g3189	7	2.45	glycosyl hydrolase family 10 protein
Ben3g11212	144	2.45	Putative NADPH dehydrogenase C23G7.10c
Ben3g4865	1	2.45	pectate lyase, putative
Ben3g6197	3	2.45	F-box-like domain protein
Ben3g5306	111	2.45	subtilisin-like serine protease family protein
Ben3g2636	86	2.44	pleiotropic drug resistance ABC transporter
Ben3g4046	1497	2.44	carboxylic acid transporter
Ben3g9090	23	2.44	hypothetical protein
Ben3g3880	89	2.44	putative phosphatase YNL010W
Ben3g1393	114	2.43	dienelactone hydrolase family protein

Ben3g10428	160	2.42	O-methylsterigmatocystin oxidoreductase
Ben3g1587	6	2.42	Apoptosis-inducing factor homolog
Ben3g10020	0	2.41	lactonase, 7-bladed beta-propeller
Ben3g12161	0	2.41	Tetratricopeptide repeat
Ben3g2618	36	2.41	peroxidase, putative
Ben3g44	4	2.40	cytochrome P450 family protein
Ben3g7749	3	2.39	3-ketoacyl-(acyl-carrier) reductase
Ben3g5280	3	2.39	pectin methylesterase family protein
Ben3g11328	51	2.39	ubiquitin C
Ben3g4864	2	2.39	pectate lyase, putative
Ben3g5034	12	2.38	pectate lyase
Ben3g11060	2	2.38	Probable glucan 1,3-beta-glucosidase D
Ben3g4139	181	2.38	laccase, multicopper oxidase, benzenediol:oxygen oxidorectutase
Ben3g6725	207	2.37	3-ketoacyl-(acyl-carrier) reductase
Ben3g11437	8	2.37	GDSL-like lipase/acylhydrolase domain protein
Ben3g136	500	2.36	NAD(P)H:quinone oxidoreductase, type IV
Ben3g3849	112	2.34	gly-Xaa carboxypeptidase
Ben3g12533	1	2.34	hypothetical protein
Ben3g475	123	2.34	BTB domain protein
Ben3g12476	15	2.33	glutathione S-transferase, carboxy-terminal domain protein
Ben3g3893	160	2.33	DNA replication protein YHM2
Ben3g2232	701	2.33	aminotransferase
Ben3g7357	38	2.32	hypothetical protein
Ben3g4574	174	2.32	flavin reductase-like domain protein
Ben3g4654	194	2.32	NADH dehydrogenase
Ben3g4917	268	2.31	oxalate decarboxylase
Ben3g11032	68	2.31	2-methylcitrate dehydratase, putative
Ben3g8494	29	2.31	MFS general substrate transporter
Ben3g7567	2	2.31	MFS general substrate transporter
Ben3g4291	103	2.31	AhpC/TSA antioxidant enzyme
Ben3g4079	8	2.31	transmembrane protein, putative
Ben3g7993	311	2.30	chondroitin AC/alginate lyase
Ben3g8785	41	2.29	fungal-specific transcription factor domain protein
Ben3g9770	15	2.29	Bifunctional solanapyrone synthase
Ben3g1278	14	2.29	lysozyme (EC:3.2.1.17)
Ben3g4875	73	2.28	adenylosuccinate lyase
Ben3g2556	18	2.28	MFS transporter
Ben3g104	5	2.28	hypothetical protein
Ben3g11888	29	2.28	Cuticle-degrading protease
Ben3g11397	18	2.28	ATP phosphoribosyltransferase
Ben3g7241	19	2.28	short chain dehydrogenase
Ben3g191	15	2.27	Siderophore iron transporter
Ben3g9479	23	2.27	hypothetical protein
Ben3g8600	4	2.27	hypothetical protein
Ben3g2195	32	2.26	Probable sulfate permease
Ben3g7059	154	2.26	F-box-like protein
Ben3g9485	308	2.26	hypothetical protein
Ben3g7620	52	2.26	homogentisate 1,2-dioxygenase
Ben3g9567	1	2.26	H-type lectin domain protein
Ben3g7554	16	2.26	putative lipase domain protein
Ben3g2087	67	2.26	Sugar phosphatase
Ben3g10297	279	2.25	hypothetical protein
Ben3g6277	120	2.25	putative aryl alcohol dehydrogenase
Ben3g3673	2	2.24	vacuolar amino acid permease, putative
Ben3g9258	18	2.24	GTP/GDP carrier protein
Ben3g7299	53	2.24	GATA zinc finger protein
Ben3g11349	3	2.24	Protein CSF1
Ben3g7587	5	2.23	galactoside-binding lectin protein
Ben3g6826	5	2.23	acetyltransferase (GNAT) family containing protein
Ben3g9279	129	2.23	cellobiohydrolase II
Ben3g5376	22	2.22	AMP-binding enzyme
Ben3g11717	3	2.22	NADH:flavin oxidoreductase/NADH oxidase
Ben3g4402	57	2.22	pyridoxal-phosphate-dependent protein
Ben3g1546	396	2.22	Metal-nicotianamine transporter
Ben3g1691	7	2.21	beta-glucosidase

Ben3g11945	3	2.21	proteophosphoglycan 5
Ben3g5886	32	2.21	hypothetical protein
Ben3g12042	1	2.20	transposase family Tnp2 protein
Ben3g12169	1	2.19	hypothetical protein
Ben3g10881	4	2.19	Ubiquitin
Ben3g2549	236	2.19	hypothetical protein
Ben3g11644	3	2.19	Retrotransposable element Tf2 155 kDa protein type 1
Ben3g2632	193	2.19	MFS monocarboxylate transporter, putative
Ben3g2952	26	2.18	O-methylsterigmatocystin oxidoreductase
Ben3g2590	197	2.18	citrate synthase
Ben3g572	5	2.18	glutathione S-transferase carboxy-terminal-like protein
Ben3g2808	133	2.18	aldo/keto reductase family protein
Ben3g7301	15	2.18	WLM domain protein
Ben3g1661	3	2.17	hypothetical protein
Ben3g701	129	2.17	dihydrodipicolinate synthetase family protein
Ben3g5477	74	2.16	hydrogenase maturation factor hoxX
Ben3g3881	193	2.16	2,3-diketo-5-methylthio-1-phosphopentane phosphatase
Ben3g5110	42	2.16	MFS general substrate transporter
Ben3g4739	1	2.15	endo-polygalacturonase PG1
Ben3g2115	198	2.15	hypothetical protein
Ben3g1093	48	2.15	hypothetical protein
Ben3g10271	146	2.15	hypothetical protein
Ben3g5030	4	2.15	endo-polygalacturonase PG1
Ben3g1700	15	2.15	glycoside hydrolase catalytic core protein
Ben3g7229	1	2.15	alpha/beta hydrolase fold protein
Ben3g9493	27	2.14	hypothetical protein
Ben3g5259	26	2.14	rare lipoprotein A-like double-psi beta-barrel protein
Ben3g397	42	2.14	MFS transporter
Ben3g11102	102	2.13	hypothetical protein
Ben3g7881	2	2.13	pectinesterase
Ben3g11576	18	2.12	hypothetical protein
Ben3g2161	17	2.11	Esterase
Ben3g5783	226	2.11	alpha-mannosyltransferase, putative
Ben3g10520	27	2.11	hypothetical protein
Ben3g8391	2	2.11	putative reverse transcriptase from transposon X-element protein
Ben3g12497	1	2.11	hypothetical protein
Ben3g11080	84	2.10	hypothetical protein
Ben3g7813	135	2.10	peroxidase family 2 domain protein
Ben3g2015	317	2.10	UDP-glucuronic acid decarboxylase
Ben3g2947	115	2.10	polyamine transporter tpo5
Ben3g4393	12	2.09	MFS sugar transporter-like protein
Ben3g3034	7	2.08	subtilisin-like serine protease family protein
Ben3g9276	17	2.08	hypothetical protein
Ben3g9861	133	2.07	putative 2-amino-3-carboxymuconate-6-semialdehyde decarboxylase
Ben3g2544	21	2.07	hypothetical protein
Ben3g7637	7	2.07	glycoside hydrolase family 10 protein
Ben3g6102	62	2.07	histidinol-phosphate aminotransferase
Ben3g455	91	2.07	permease, cytosine/purine, uracil, thiamine, allantoin family protein
Ben3g8908	67	2.07	proline dehydrogenase
Ben3g5297	441	2.06	polysaccharide lyase family 8 protein
Ben3g12312	20	2.06	hypothetical protein
Ben3g6273	3	2.06	3-ketoacyl-(acyl-carrier) reductase
Ben3g7885	127	2.05	pantoate--beta-alanine ligase
Ben3g10105	42	2.05	NADH:flavin oxidoreductase/NADH oxidase
Ben3g4712	19	2.05	hypothetical protein
Ben3g4521	54	2.05	transporter, monovalent cation:proton antiporter-2 (CPA2) family protein
Ben3g5098	24	2.04	exopolygalacturonase, putative
Ben3g7851	322	2.04	hypothetical protein
Ben3g4057	13	2.04	L-mandelate dehydrogenase
Ben3g6915	7	2.04	putative lipase from carbohydrate esterase family CE10 protein
Ben3g5983	2035	2.04	3-deoxy-7-phosphoheptulonate synthase
Ben3g9764	2	2.04	6-hydroxy-D-nicotine oxidase
Ben3g8539	9	2.02	phytanoyl-CoA dioxygenase
Ben3g11995	33	2.02	Choline dehydrogenase
Ben3g8624	2	2.02	Benzoate 4-monooxygenase

Ben3g12489	4	2.02	surface layer protein, putative
Ben3g1893	44	2.01	aldo/keto reductase
Ben3g2462	20	2.01	methylenetetrahydrofolate dehydrogenase (NAD+)
Ben3g4134	14	2.01	endo-1,4-beta-xylanase
Ben3g4357	41	2.01	hypothetical protein
Ben3g8073	21	2.01	ketopantoate reductase PanE/ApbA carboxy-terminal protein
Ben3g4052	1	2.01	pectate lyase
Ben3g7630	209	2.00	hypothetical protein
Ben3g4643	346	2.00	hypothetical protein
Ben3g10175	56	2.00	glycoside hydrolase family 61 protein
Ben3g924	2114	-2.00	hypothetical protein
Ben3g3477	13	-2.00	hypothetical protein
Ben3g2721	230	-2.00	patatin-like phospholipase
Ben3g3339	22	-2.00	acetylcholinesterase
Ben3g4406	1	-2.01	related to alpha-galactosidase precursor
Ben3g11026	7	-2.02	hypothetical protein
Ben3g2776	316	-2.02	hypothetical protein
Ben3g6772	221	-2.02	oxidoreductase, putative
Ben3g2193	383	-2.02	hypothetical protein
Ben3g10025	3	-2.02	hypothetical protein
Ben3g1940	2	-2.02	hypothetical protein
Ben3g10026	2	-2.02	hypothetical protein
Ben3g7694	3	-2.02	Nitrogen assimilation transcription factor nit-4
Ben3g10598	131	-2.02	hypothetical protein
Ben3g10729	23	-2.02	vegetative incompatibility protein HET-E-1
Ben3g8669	4	-2.03	hypothetical protein
Ben3g4340	913	-2.03	hypothetical protein
Ben3g8800	2604	-2.03	hypothetical protein
Ben3g12080	5	-2.03	hypothetical protein
Ben3g3053	6	-2.03	tRNA-splicing ligase RtcB
Ben3g325	128	-2.03	hypothetical protein
Ben3g8443	12	-2.03	hypothetical protein
Ben3g4786	3	-2.03	Lipase
Ben3g11516	3	-2.03	hypothetical protein
Ben3g6609	10	-2.04	tyrosine kinase domain protein
Ben3g10106	13	-2.04	hypothetical protein
Ben3g6035	9	-2.04	hypothetical protein
Ben3g9607	25	-2.04	hypothetical protein
Ben3g2900	13	-2.04	small nuclear ribonucleoprotein E
Ben3g5705	64	-2.04	hypothetical protein
Ben3g4893	10	-2.05	hypothetical protein
Ben3g6127	28	-2.05	hypothetical protein
Ben3g2428	20	-2.05	hypothetical protein
Ben3g11616	37	-2.05	hypothetical protein
Ben3g9612	19	-2.05	hypothetical protein
Ben3g8116	31	-2.05	hypothetical protein
Ben3g26	65	-2.06	hypothetical protein
Ben3g4281	15	-2.06	hypothetical protein
Ben3g4561	3	-2.06	hypothetical protein
Ben3g6316	1	-2.06	hypothetical protein
Ben3g6146	55	-2.06	hypothetical protein
Ben3g10259	1	-2.06	hypothetical protein
Ben3g9777	32	-2.07	cytochrome P450 family protein
Ben3g3276	216	-2.07	hypothetical protein
Ben3g10936	61	-2.07	aromatic di-alanine and TPR containing protein
Ben3g9761	7	-2.07	hypothetical protein
Ben3g9549	95	-2.07	hypothetical protein
Ben3g3431	11	-2.07	tyrosine kinase catalytic domain protein
Ben3g5322	5	-2.08	kinesin light chain
Ben3g1008	36	-2.08	hypothetical protein
Ben3g2393	19	-2.08	JmjC domain-containing protein 7
Ben3g6289	49	-2.08	hypothetical protein
Ben3g10136	27	-2.08	response regulator receiver domain protein
Ben3g11499	5	-2.09	vegetative incompatibility protein HET-E-1
Ben3g2610	25	-2.09	Siderophore iron transporter 3

Ben3g7757	5	-2.09	hypothetical protein
Ben3g7699	202	-2.09	tyrosine kinase catalytic domain protein
Ben3g10626	18	-2.09	hypothetical protein
Ben3g9632	37	-2.09	hypothetical protein
Ben3g997	33	-2.09	hypothetical protein
Ben3g12261	2	-2.10	hypothetical protein
Ben3g3114	49	-2.10	hypothetical protein
Ben3g7104	10	-2.10	hypothetical protein
Ben3g8012	80	-2.10	hypothetical protein
Ben3g4937	30	-2.10	hypothetical protein
Ben3g11503	26	-2.10	mucoidy inhibitor A
Ben3g8074	65	-2.11	cytochrome P450 family protein
Ben3g2957	2	-2.11	hypothetical protein
Ben3g9358	18	-2.11	hypothetical protein
Ben3g11176	8	-2.11	hypothetical protein
Ben3g676	87	-2.11	hypothetical protein
Ben3g1444	11	-2.11	hypothetical protein
Ben3g5785	43	-2.12	alpha,alpha-trehalose-phosphate synthase
Ben3g6243	30	-2.12	hypothetical protein
Ben3g10170	81	-2.12	hypothetical protein
Ben3g2912	44	-2.12	hypothetical protein
Ben3g11031	3	-2.12	hypothetical protein
Ben3g8191	14	-2.13	hypothetical protein
Ben3g6260	11	-2.13	hypothetical protein
Ben3g10798	5	-2.13	hypothetical protein
Ben3g10037	2	-2.13	Cellulose-growth-specific protein
Ben3g10337	5	-2.13	hypothetical protein
Ben3g1972	73	-2.13	kinesin light chain
Ben3g5578	40	-2.13	hypothetical protein
Ben3g7627	58	-2.14	Putative NADPH-dependent methylglyoxal reductase GRP2
Ben3g3504	1	-2.14	hypothetical protein
Ben3g9406	12	-2.15	hypothetical protein
Ben3g11048	5	-2.15	hypothetical protein
Ben3g2363	88	-2.15	hypothetical protein
Ben3g10322	59	-2.15	hypothetical protein
Ben3g225	13	-2.15	hypothetical protein
Ben3g8446	1	-2.15	hypothetical protein
Ben3g5988	19	-2.15	hypothetical protein
Ben3g8617	18	-2.16	hypothetical protein
Ben3g5764	19	-2.16	permease/ATP-binding ABC transporter
Ben3g8684	93	-2.16	hypothetical protein
Ben3g11974	34	-2.16	calpain-1 catalytic subunit
Ben3g4655	74	-2.16	Cytochrome P450 4V2
Ben3g2570	24	-2.16	glucose oxidase-like protein
Ben3g3925	14	-2.17	hypothetical protein
Ben3g2392	123	-2.17	hypothetical protein
Ben3g7963	107	-2.17	deuterolysin metalloprotease (M35) family containing protein
Ben3g349	30	-2.17	hypothetical protein
Ben3g7119	15	-2.17	3-ketoacyl-(acyl-carrier) reductase
Ben3g10528	1	-2.17	DNA mismatch repair protein MutS
Ben3g1949	37	-2.17	AcnD-accessory protein PrpF, putative
Ben3g8761	23	-2.17	hypothetical protein
Ben3g10318	914	-2.17	RSN1-overexpression rescues sro7/sop1 in NaCl-like, putative
Ben3g9210	4	-2.18	mitochondrial mRNA processing protein
Ben3g7344	3	-2.18	hypothetical protein
Ben3g8757	44	-2.18	hypothetical protein
Ben3g7607	44	-2.18	aromatic di-alanine and TPR containing protein
Ben3g1259	244	-2.18	putative mucoidy inhibitor A
Ben3g3418	106	-2.18	fungal specific transcription factor domain protein
Ben3g5857	79	-2.18	minor extracellular protease vpr protein
Ben3g10574	9	-2.19	hypothetical protein
Ben3g527	63	-2.19	hypothetical protein
Ben3g8217	2	-2.19	hypothetical protein
Ben3g9103	17	-2.19	tyrosine kinase family catalytic domain protein
Ben3g11624	34	-2.19	hypothetical protein

Ben3g5793	9	-2.19	indoleamine 2,3-dioxygenase
Ben3g7524	84	-2.20	hypothetical protein
Ben3g4093	17	-2.20	phthalate transporter
Ben3g5878	30	-2.20	hypothetical protein
Ben3g9193	5	-2.21	hypothetical protein
Ben3g4459	200	-2.22	hypothetical protein
Ben3g1481	47	-2.22	hypothetical protein
Ben3g5786	957	-2.22	transporter ATM1
Ben3g2430	20	-2.22	hypothetical protein
Ben3g10107	125	-2.23	vegetative incompatibility protein HET-E-1, putative
Ben3g4178	52	-2.23	hypothetical protein
Ben3g272	11	-2.23	hypothetical protein
Ben3g8935	5	-2.23	Putative hydrolase Mb2247c
Ben3g4253	162	-2.23	hypothetical protein
Ben3g3580	4	-2.23	hypothetical protein
Ben3g9486	4	-2.23	cytochrome P450 family monooxygenase pc-3
Ben3g11612	60	-2.24	hypothetical protein
Ben3g4610	56	-2.24	hypothetical protein
Ben3g10681	2	-2.24	hypothetical protein
Ben3g6000	156	-2.24	hypothetical protein
Ben3g12140	2	-2.24	hypothetical protein
Ben3g9133	10	-2.24	tyrosine kinase catalytic domain protein
Ben3g6406	5	-2.24	O-methylsterigmatocystin oxidoreductase
Ben3g5485	12	-2.24	vegetative incompatibility protein HET-E-1
Ben3g8361	89	-2.24	putative vegetative incompatibility protein HET-E-1
Ben3g7482	16	-2.25	extracellular metalloprotease
Ben3g2354	27	-2.25	hypothetical protein
Ben3g7621	15	-2.25	hypothetical protein
Ben3g7327	264	-2.25	hypothetical protein
Ben3g510	34	-2.25	hypothetical protein
Ben3g9627	9	-2.25	hypothetical protein
Ben3g12302	5	-2.25	hypothetical protein
Ben3g11074	44	-2.25	hypothetical protein
Ben3g2661	52	-2.25	hypothetical protein
Ben3g5028	7	-2.25	hypothetical protein
Ben3g7683	7	-2.25	cutinase Cut4
Ben3g9323	70	-2.26	hypothetical protein
Ben3g6522	511	-2.26	Dyp-type peroxidase
Ben3g5880	4	-2.27	hypothetical protein
Ben3g8551	1	-2.27	hypothetical protein
Ben3g12138	5	-2.28	hypothetical protein
Ben3g2598	24	-2.28	vegetative incompatibility protein HET-E-1, putative
Ben3g10753	12	-2.28	hypothetical protein
Ben3g4899	2	-2.28	hypothetical protein
Ben3g7213	5	-2.29	hypothetical protein
Ben3g3575	6	-2.29	hypothetical protein
Ben3g8139	374	-2.29	hypothetical protein
Ben3g3335	21	-2.30	hypothetical protein
Ben3g6239	6	-2.30	GDSL-like lipase/acylhydrolase
Ben3g8597	2	-2.30	hypothetical protein
Ben3g5736	45	-2.31	hypothetical protein
Ben3g11897	2	-2.31	hypothetical protein
Ben3g1761	1175	-2.32	hypothetical protein
Ben3g4622	5	-2.32	extracellular metalloprotease
Ben3g8991	342	-2.32	MFS transporter
Ben3g12448	3	-2.32	hypothetical protein
Ben3g11543	2	-2.33	hypothetical protein
Ben3g9705	36	-2.33	hypothetical protein
Ben3g8190	24	-2.33	hypothetical protein
Ben3g6471	9	-2.34	hypothetical protein
Ben3g8919	4	-2.34	hypothetical protein
Ben3g10047	1	-2.34	glycosyltransferase family 2 protein
Ben3g8355	121	-2.35	hypothetical protein
Ben3g6538	121	-2.35	superoxide dismutase [Cu-Zn] protein
Ben3g11686	2	-2.35	endo-1,4-beta-xylanase, partial

Ben3g9783	17	-2.35	hypothetical protein
Ben3g11492	1	-2.35	glycine dehydrogenase
Ben3g6571	12	-2.35	hypothetical protein
Ben3g3112	5	-2.35	hypothetical protein
Ben3g7794	5	-2.35	hypothetical protein
Ben3g12518	1	-2.36	hypothetical protein
Ben3g10330	6	-2.36	endonuclease/exonuclease/phosphatase family protein
Ben3g10931	0	-2.36	Transposon Ty3-I Gag-Pol polyprotein
Ben3g5261	15	-2.37	hypothetical protein
Ben3g4299	8	-2.37	hypothetical protein
Ben3g8405	29	-2.37	hypothetical protein
Ben3g4992	360	-2.39	hypothetical protein
Ben3g4827	385	-2.39	hypothetical protein
Ben3g2662	7	-2.39	hypothetical protein
Ben3g8616	1	-2.39	hypothetical protein
Ben3g8463	0	-2.39	peroxisomal adenine nucleotide transporter 1, putative
Ben3g12273	15	-2.40	hypothetical protein
Ben3g4898	156	-2.40	hypothetical protein
Ben3g2806	56	-2.40	hypothetical protein
Ben3g6629	9	-2.40	hypothetical protein
Ben3g10845	0	-2.41	DnaK family protein
Ben3g9211	4	-2.41	hypothetical protein
Ben3g3038	26	-2.41	hypothetical protein
Ben3g4676	27	-2.41	cytochrome P450 family protein
Ben3g10385	3	-2.41	hypothetical protein
Ben3g1689	2	-2.41	pectate lyase
Ben3g9610	10	-2.41	hypothetical protein
Ben3g4822	42	-2.42	putative glucan 1,3-beta-glucosidase D
Ben3g6950	1	-2.42	hypothetical protein
Ben3g8858	8	-2.42	hypothetical protein
Ben3g7725	14	-2.42	hypothetical protein
Ben3g12154	3	-2.42	hypothetical protein
Ben3g9206	3	-2.43	hypothetical protein
Ben3g8114	27	-2.43	putative phosphate ABC transporter, ATP-binding protein
Ben3g5035	32	-2.44	cryptochrome, DASH family protein
Ben3g8727	116	-2.44	hypothetical protein
Ben3g4175	13	-2.44	hypothetical protein
Ben3g7846	0	-2.45	hypothetical protein
Ben3g9437	0	-2.45	polysaccharide lyase family 4 protein
Ben3g10493	48	-2.45	helicase carboxy-terminal domain protein
Ben3g4437	2	-2.45	hypothetical protein
Ben3g6635	33	-2.45	hypothetical protein
Ben3g7231	15	-2.46	hypothetical protein
Ben3g5286	8	-2.46	hypothetical protein
Ben3g367	230	-2.46	hypothetical protein
Ben3g8219	13	-2.46	kinesin light chain
Ben3g6017	40	-2.46	hypothetical protein
Ben3g3415	32	-2.46	hypothetical protein
Ben3g12355	23	-2.46	hypothetical protein
Ben3g10942	152	-2.47	heat shock 70 kDa protein 12A
Ben3g12407	1	-2.47	poly(A) polymerase
Ben3g11142	60	-2.47	hypothetical protein
Ben3g1703	1	-2.47	hypothetical protein
Ben3g505	790	-2.47	hypothetical protein
Ben3g5564	2	-2.47	fruiting body protein Sc7
Ben3g6262	176	-2.48	UvrD-like helicase carboxy-terminal domain protein
Ben3g1092	252	-2.48	SGT1, suppressor of G2 allele of SKP1 protein, putative
Ben3g1834	14	-2.48	hypothetical protein
Ben3g2325	3	-2.49	DUF1992 domain protein
Ben3g7751	8	-2.49	hypothetical protein
Ben3g11978	3	-2.49	hypothetical protein
Ben3g7122	266	-2.49	flavorubredoxin reductase
Ben3g8901	19	-2.50	hypothetical protein
Ben3g5453	1	-2.50	hypothetical protein
Ben3g8082	8	-2.50	hypothetical protein

Ben3g8359	62	-2.50	putative vegetative incompatibility protein HET-E-1
Ben3g1349	11	-2.51	Pectinesterase
Ben3g3378	3	-2.51	hypothetical protein
Ben3g2419	116	-2.51	RNA-dependent RNA polymerase
Ben3g10680	40	-2.52	hypothetical protein
Ben3g7026	2	-2.53	hypothetical protein
Ben3g11040	136	-2.53	cytochrome P450 family protein
Ben3g11290	4	-2.53	vegetative incompatibility protein HET-E-1
Ben3g5597	73	-2.53	hypothetical protein
Ben3g10108	24	-2.53	hypothetical protein
Ben3g9016	5	-2.53	glycoside hydrolase family 61 protein
Ben3g10754	18	-2.53	hypothetical protein
Ben3g11164	20	-2.54	hypothetical protein
Ben3g9230	95	-2.54	cytochrome P450 family monooxygenase
Ben3g5404	1	-2.55	hypothetical protein
Ben3g9501	3	-2.55	hypothetical protein
Ben3g10079	41	-2.55	formate/nitrite transporter family protein
Ben3g11522	1	-2.55	hypothetical protein
Ben3g6896	2	-2.56	tyrosinase tyrosinase: common central domain protein
Ben3g5823	30	-2.57	hypothetical protein
Ben3g12450	8	-2.57	hypothetical protein
Ben3g5308	60	-2.57	subtilisin-like serine protease family protein
Ben3g10641	16	-2.57	hypothetical protein
Ben3g5653	1	-2.58	hypothetical protein
Ben3g1818	136	-2.58	mannose-6-phosphate isomerase
Ben3g4271	1	-2.58	cytochrome P450 family protein
Ben3g4150	84	-2.58	hypothetical protein
Ben3g10083	305	-2.58	calpain-1 catalytic subunit
Ben3g7830	4	-2.59	hypothetical protein
Ben3g9070	1	-2.59	putative casein kinase
Ben3g755	381	-2.59	VHS domain protein
Ben3g11588	65	-2.59	aromatic di-alanine and TPR containing protein
Ben3g7363	247	-2.59	von willebrand factor type A domain protein
Ben3g4710	3	-2.59	hypothetical protein
Ben3g10320	71	-2.61	3-ketoacyl-(acyl-carrier) reductase
Ben3g2513	4	-2.62	hypothetical protein
Ben3g7215	24	-2.63	hypothetical protein
Ben3g7676	18	-2.63	hypothetical protein
Ben3g6742	602	-2.63	UV excision repair protein RAD23
Ben3g3168	27	-2.64	hypothetical protein
Ben3g10612	14	-2.64	hypothetical protein
Ben3g6397	3	-2.64	endonuclease/exonuclease/phosphatase family protein
Ben3g4728	20	-2.64	patatin-like phospholipase
Ben3g5619	237	-2.64	putative hydrolase
Ben3g1124	3	-2.65	hypothetical protein
Ben3g2659	59	-2.65	permease, cytosine/purine, uracil, thiamine, allantoin
Ben3g3357	10	-2.65	hypothetical protein
Ben3g7251	19	-2.65	hypothetical protein
Ben3g8925	25	-2.65	hypothetical protein
Ben3g10070	1	-2.66	integrase core domain protein
Ben3g11538	1	-2.66	hypothetical protein
Ben3g9836	19	-2.67	hypothetical protein
Ben3g7250	21	-2.67	hypothetical protein
Ben3g7656	9	-2.67	aromatic di-alanine and TPR containing protein
Ben3g3441	10	-2.68	hypothetical protein
Ben3g1944	53	-2.69	hypothetical protein
Ben3g7723	73	-2.69	hypothetical protein
Ben3g3133	4	-2.69	glutathione S-transferase
Ben3g4407	221	-2.69	hypothetical protein
Ben3g11587	13	-2.70	hypothetical protein
Ben3g6931	4	-2.70	hypothetical protein
Ben3g10906	1	-2.70	hypothetical protein
Ben3g2316	169	-2.70	hypothetical protein
Ben3g8228	71	-2.71	hypothetical protein
Ben3g5953	184	-2.71	FAD-linked oxidoreductase, putative

Ben3g10176	8	-2.71	hypothetical protein
Ben3g7517	40	-2.71	F-box protein
Ben3g8891	5	-2.72	MFS general substrate transporter
Ben3g10894	1	-2.72	hypothetical protein
Ben3g12420	1	-2.72	hypothetical protein
Ben3g6970	4	-2.73	hypothetical protein
Ben3g7334	8	-2.73	hypothetical protein
Ben3g2257	108	-2.73	major facilitator (MFS1) transporter-like protein
Ben3g1704	418	-2.74	band 7 family protein, putative
Ben3g9878	10	-2.74	hypothetical protein
Ben3g6488	41	-2.74	Putative 30S ribosomal protein S17P-like
Ben3g11175	12	-2.75	hypothetical protein
Ben3g9095	11	-2.75	tyrosine kinase family catalytic domain protein
Ben3g11848	1	-2.75	hypothetical protein
Ben3g11471	11	-2.76	hypothetical protein
Ben3g6676	35	-2.76	hypothetical protein
Ben3g10021	1	-2.76	reverse transcriptase
Ben3g8938	287	-2.78	myxovirus resistance protein
Ben3g6965	17	-2.78	hypothetical protein
Ben3g9907	73	-2.79	aromatic di-alanine and TPR containing protein
Ben3g7657	196	-2.80	aromatic di-alanine and TPR containing protein
Ben3g2365	39	-2.80	hypothetical protein
Ben3g6461	12	-2.81	hypothetical protein
Ben3g9512	1	-2.81	hypothetical protein
Ben3g1945	35	-2.81	hypothetical protein
Ben3g4693	1	-2.82	hypothetical protein
Ben3g8513	187	-2.82	Putative 30S ribosomal protein S17P-like
Ben3g3589	5	-2.83	hypothetical protein
Ben3g2741	20	-2.84	hypothetical protein
Ben3g1004	117	-2.84	heat shock protein HSP104, putative
Ben3g9119	40	-2.84	aromatic di-alanine and TPR containing protein
Ben3g7898	2	-2.85	endonuclease/exonuclease/phosphatase family protein
Ben3g12033	27	-2.86	hypothetical protein
Ben3g11873	7	-2.86	hypothetical protein
Ben3g1213	509	-2.87	peroxisomal membrane protein pex16
Ben3g10640	1	-2.87	hypothetical protein
Ben3g8464	194	-2.87	Albumin-2
Ben3g6311	10	-2.88	hypothetical protein
Ben3g7235	160	-2.88	hypothetical protein
Ben3g1925	20	-2.88	arginase family protein
Ben3g8161	189	-2.89	hypothetical protein
Ben3g8734	23	-2.89	cytochrome P450 family protein
Ben3g11629	4	-2.90	Putative 30S ribosomal protein S17P-like
Ben3g7367	114	-2.91	hypothetical protein
Ben3g7294	21	-2.91	hypothetical protein
Ben3g5193	3	-2.91	hypothetical protein
Ben3g8947	42	-2.92	carbohydrate esterase family 4 protein
Ben3g5860	5	-2.92	tyrosinase tyrosinase: common central domain protein
Ben3g6559	149	-2.93	hypothetical protein
Ben3g4206	1	-2.93	hypothetical protein
Ben3g3352	415	-2.93	putative short-chain dehydrogenase/reductase
Ben3g8246	21	-2.93	hypothetical protein
Ben3g9247	13	-2.94	Putative 30S ribosomal protein S17P-like
Ben3g11162	2	-2.94	hypothetical protein
Ben3g10877	227	-2.94	Smr domain protein C11H11,03c
Ben3g12536	6	-2.96	3-ketoacyl-(acyl-carrier) reductase
Ben3g532	74	-2.96	hypothetical protein
Ben3g8218	56	-2.97	cytokinesis protein sepA
Ben3g12342	7	-2.97	hypothetical protein
Ben3g10835	14	-2.98	hypothetical protein
Ben3g4277	23	-2.99	O-methylsterigmatocystin oxidoreductase
Ben3g9782	7	-2.99	hypothetical protein
Ben3g8351	183	-2.99	hypothetical protein
Ben3g2499	42	-3.00	hypothetical protein
Ben3g8846	60	-3.01	hypothetical protein

Ben3g5394	6	-3.03	hypothetical protein
Ben3g12212	3	-3.03	hypothetical protein
Ben3g2006	10	-3.03	tyrosine kinase catalytic domain protein
Ben3g4757	319	-3.03	hypothetical protein
Ben3g5650	11	-3.03	vegetative incompatibility protein HET-E-1
Ben3g4800	14	-3.04	hypothetical protein
Ben3g4213	8	-3.06	hypothetical protein
Ben3g11844	40	-3.06	hypothetical protein
Ben3g7651	10	-3.06	aromatic di-alanine and TPR containing protein
Ben3g10611	36	-3.06	hypothetical protein
Ben3g8613	566	-3.08	Dyp-type peroxidase
Ben3g3437	177	-3.08	tyrosinase tyrosinase: common central domain protein
Ben3g8180	22	-3.09	hypothetical protein
Ben3g6271	25	-3.09	enoyl-(acyl carrier) reductase
Ben3g10260	232	-3.09	hypothetical protein
Ben3g12020	3	-3.10	hypothetical protein
Ben3g7596	303	-3.11	lipase class 3
Ben3g3830	9	-3.12	hypothetical protein
Ben3g7857	3	-3.14	streptomycin biosynthesis protein StrI
Ben3g8353	29	-3.14	hypothetical protein
Ben3g9994	11	-3.14	hypothetical protein
Ben3g11172	6	-3.15	putative vegetative incompatibility protein HET-E-1
Ben3g9595	759	-3.15	hypothetical protein
Ben3g10500	7	-3.15	hypothetical protein
Ben3g7658	5	-3.15	hypothetical protein
Ben3g2519	6	-3.16	hypothetical protein
Ben3g3607	93	-3.17	hypothetical protein
Ben3g4809	34	-3.18	hypothetical protein
Ben3g7668	1	-3.18	hypothetical protein
Ben3g6374	6	-3.18	Putative 30S ribosomal protein S17P-like
Ben3g6028	588	-3.19	hypothetical protein
Ben3g7352	4	-3.19	Putative 30S ribosomal protein S17P-like
Ben3g11140	39	-3.22	hypothetical protein
Ben3g12461	2	-3.23	hypothetical protein
Ben3g12440	10	-3.23	hypothetical protein
Ben3g6434	8	-3.24	hypothetical protein
Ben3g7247	1	-3.24	hypothetical protein
Ben3g12035	12	-3.25	putative vegetative incompatibility protein HET-E-1
Ben3g8587	4	-3.25	hypothetical protein
Ben3g8548	1	-3.25	Putative 30S ribosomal protein S17P-like
Ben3g2516	75	-3.25	Putative 30S ribosomal protein S17P-like
Ben3g5314	12	-3.25	Putative 30S ribosomal protein S17P-like
Ben3g8794	11	-3.27	hypothetical protein
Ben3g11325	6	-3.27	Putative 30S ribosomal protein S17P-like
Ben3g4830	373	-3.27	hypothetical protein
Ben3g7659	10	-3.28	hypothetical protein
Ben3g9972	1	-3.29	hypothetical protein
Ben3g12279	1	-3.30	hypothetical protein
Ben3g4130	16	-3.30	hypothetical protein
Ben3g7137	1	-3.33	hypothetical protein
Ben3g7701	85	-3.34	hypothetical protein
Ben3g7	30	-3.34	vegetative incompatibility protein HET-E-1
Ben3g2532	10	-3.34	hypothetical protein
Ben3g11133	2	-3.34	hypothetical protein
Ben3g8758	28	-3.35	hypothetical protein
Ben3g2599	92	-3.36	hypothetical protein
Ben3g7779	214	-3.37	Dyp-type peroxidase
Ben3g9835	47	-3.38	hypothetical protein
Ben3g2359	19	-3.39	hypothetical protein
Ben3g2941	64	-3.39	hypothetical protein
Ben3g12381	8	-3.39	hypothetical protein
Ben3g11131	1	-3.41	hypothetical protein
Ben3g4183	22	-3.42	hypothetical protein
Ben3g2062	158	-3.42	hypothetical protein
Ben3g6414	200	-3.43	ICE-like protease (caspase) p20 domain protein

Ben3g5666	2	-3.44	hypothetical protein
Ben3g9790	3	-3.44	hypothetical protein
Ben3g10	67	-3.44	hypothetical protein
Ben3g12060	61	-3.45	hypothetical protein
Ben3g11904	15	-3.46	hypothetical protein
Ben3g1789	5	-3.47	hypothetical protein
Ben3g11324	32	-3.47	Putative 30S ribosomal protein S17P-like
Ben3g6991	4	-3.47	hypothetical protein
Ben3g10182	98	-3.47	metallopeptidase MepB
Ben3g4958	193	-3.49	subtilisin-like serine protease family protein
Ben3g11590	53	-3.52	Putative 30S ribosomal protein S17P-like
Ben3g11472	13	-3.53	hypothetical protein
Ben3g3479	2	-3.57	hypothetical protein
Ben3g10804	23	-3.57	hypothetical protein
Ben3g2488	12	-3.58	Putative 30S ribosomal protein S17P-like
Ben3g8644	29	-3.58	hypothetical protein
Ben3g8788	10	-3.58	hypothetical protein
Ben3g12475	5	-3.59	hypothetical protein
Ben3g6173	114	-3.64	hypothetical protein
Ben3g11562	27	-3.65	hypothetical protein
Ben3g5449	63	-3.66	membrane protein TVP38
Ben3g6500	29	-3.67	Putative 30S ribosomal protein S17P-like
Ben3g11567	21	-3.68	vegetative incompatibility protein HET-E-1
Ben3g2518	2	-3.72	Putative 30S ribosomal protein S17P-like
Ben3g9851	5	-3.73	tyrosine kinase catalytic domain protein
Ben3g6936	172	-3.74	putative hydrolase
Ben3g5443	7	-3.76	hypothetical protein
Ben3g10636	37	-3.77	hypothetical protein
Ben3g11502	16	-3.77	hypothetical protein
Ben3g7754	378	-3.80	peptidyl-prolyl cis-trans isomerase
Ben3g8848	25	-3.81	hypothetical protein
Ben3g8843	30	-3.83	hypothetical protein
Ben3g5317	75	-3.88	putative ubiquitin family protein
Ben3g12530	2	-3.93	hypothetical protein
Ben3g12384	119	-3.95	hypothetical protein
Ben3g7536	74	-3.96	hypothetical protein
Ben3g8313	37	-3.98	hypothetical protein
Ben3g7065	35	-4.00	C4-dicarboxylate transporter/malic acid transporter
Ben3g10821	7	-4.03	putative vegetative incompatibility protein HET-E-1
Ben3g2184	179	-4.03	putative MFS sugar transporter
Ben3g7030	61	-4.03	hemerythrin HHE cation-binding domain protein
Ben3g3829	652	-4.04	hypothetical protein
Ben3g10363	2	-4.05	hypothetical protein
Ben3g8992	30	-4.06	hypothetical protein
Ben3g7353	8	-4.11	hypothetical protein
Ben3g8286	3	-4.11	hypothetical protein
Ben3g2533	2	-4.15	hypothetical protein
Ben3g6494	60	-4.18	Putative 30S ribosomal protein S17P-like
Ben3g9373	6	-4.20	hypothetical protein
Ben3g6667	17	-4.20	hypothetical protein
Ben3g10797	73	-4.33	hypothetical protein
Ben3g10044	5	-4.35	glycosyltransferase family 8 protein
Ben3g7242	5	-4.36	hypothetical protein
Ben3g8352	2211	-4.37	hypothetical protein
Ben3g11336	13	-4.39	hypothetical protein
Ben3g12344	72	-4.44	hypothetical protein
Ben3g10805	86	-4.49	hypothetical protein
Ben3g2402	35	-4.54	hypothetical protein
Ben3g9443	3	-4.55	kinesin light chain
Ben3g9837	103	-4.56	hypothetical protein
Ben3g9194	6	-4.59	hypothetical protein
Ben3g6561	45	-4.60	hypothetical protein
Ben3g7354	25	-4.63	Putative 30S ribosomal protein S17P-like
Ben3g6493	29	-4.65	hypothetical protein
Ben3g9367	42	-4.69	hypothetical protein

Ben3g10800	65	-4.73	hypothetical protein
Ben3g1637	976	-4.94	hypothetical protein
Ben3g7892	185	-4.94	hypothetical protein
Ben3g8592	1516	-4.00	hypothetical protein
Ben3g8849	83	-5.08	hypothetical protein
Ben3g10164	340	-5.25	hypothetical protein
Ben3g9368	4138	-5.44	hypothetical protein
Ben3g12400	64	-5.52	hypothetical protein
Ben3g7031	1073	-6.23	hemerythrin HHE cation-binding domain protein
Ben3g8963	661	-8.35	hypothetical protein

hypothetical proteins
putative transcriptional regulators
involved in protein degradation
involved in cell wall degradation
lectin domain containing proteins
specifically mentioned proteins
nitrogen assimilation
bold SeqNames are in common 3dpi-Ben and 8dpi-Ben

Supplemental Table 9: List of genes differentially transcribed at 8 dpi

SeqName	BaseMean	log2FoldChange	Description
Ben3g6247	590	12.06	RTA1-domain protein
Ben3g2563	335	11.39	cytochrome P450 family 6 protein
Ben3g5920	50	9.38	C4-dicarboxylate transporter/malic acid transporter
Ben3g9965	31	9.14	protocatechuate 3,4-dioxygenase beta subunit, putative
Ben3g8007	139	8.93	polysaccharide lyase family 1 protein
Ben3g1929	69	8.80	putative extracellular dioxygenase
Ben3g5454	114	8.73	xyloglucan-specific endo-beta-1,4-glucanase A
Ben3g7787	53	8.64	chitin-binding domain protein
Ben3g2619	27	8.33	peroxidase, putative
Ben3g8431	51	8.25	aminopeptidase
Ben3g5368	97	8.20	alpha/beta hydrolase fold protein
Ben3g3800	106	8.19	glycoside hydrolase family 18 protein
Ben3g818	69	8.19	transmembrane protein, putative
Ben3g7223	20	8.14	pectin lyase
Ben3g1242	64	8.14	cutinase
Ben3g4553	17	8.12	C2H2-type zinc-finger protein
Ben3g8486	14	7.99	hypothetical protein
Ben3g8979	28	7.81	putative transmembrane protein
Ben3g3530	181	7.79	pectate lyase, putative
Ben3g8133	13	7.69	hypothetical protein
Ben3g9480	132	7.57	hypothetical protein
Ben3g5299	9	7.53	glycoside hydrolase family 61 protein
Ben3g6837	14	7.53	FMN-dependent dehydrogenase
Ben3g1717	15	7.46	lactonase, 7-bladed beta-propeller
Ben3g1779	208	7.43	MFS multidrug transporter, putative
Ben3g5383	14	7.42	glycoside hydrolase family 61 protein
Ben3g5936	45	7.41	glycoside hydrolase family 18 protein
Ben3g4785	10	7.41	lipase
Ben3g6353	192	7.34	endo-polygalacturonase PG1
Ben3g9325	15	7.29	carbohydrate esterase family 4 protein
Ben3g2997	68	7.25	carboxyphosphonoenolpyruvate phosphonomutase, putative
Ben3g2090	59	7.15	putative transmembrane protein
Ben3g8474	15	7.15	hypothetical protein
Ben3g8251	46	7.12	Cuticle-degrading protease
Ben3g1457	10	7.11	family 16 endo-1,3(4)-beta-glucanase from glycoside hydrolase
Ben3g8250	115	7.07	peptidase S8/S53 subtilisin kexin sedolisin
Ben3g3368	11	7.00	pectate lyase, putative
Ben3g4845	41	6.90	transmembrane protein, putative
Ben3g7552	138	6.87	glycoside hydrolase family 1 protein
Ben3g12473	8	6.87	pectate lyase, putative
Ben3g8740	27	6.85	glycoside hydrolase family 61 protein
Ben3g9025	13	6.80	endoglucanase II
Ben3g54	14	6.78	NADPH-dependent aldehyde reductase
Ben3g7378	79	6.73	lipase from carbohydrate esterase family CE10 protein, putative
Ben3g2852	8	6.65	putative cutinase Mb2006c
Ben3g4382	47	6.64	4-hydroxybenzoate polyprenyltransferase
Ben3g9957	75	6.54	GMC oxidoreductase
Ben3g8198	75	6.52	glycoside hydrolase family 61 protein
Ben3g2720	10	6.43	5'/3'-nucleotidase SurE family protein
Ben3g9172	7	6.43	amidohydrolase
Ben3g4748	90	6.41	serine protease
Ben3g4236	25	6.29	hypothetical protein
Ben3g11520	3	6.27	SNF2 family amino-terminal protein
Ben3g7568	9	6.18	homeobox domain protein
Ben3g2071	543	6.17	extracellular metalloprotease
Ben3g11537	3	6.17	hypothetical protein
Ben3g7870	34	6.17	glycoside hydrolase family 3 protein
Ben3g7261	29	6.11	putative pectate lyase
Ben3g6555	33	6.10	GDSL-like lipase/acylhydrolase
Ben3g28	144	6.08	pectate lyase
Ben3g5324	655	6.07	glycoside hydrolase family protein
Ben3g8294	62	6.02	macrofage activating glycoprotein, putative
Ben3g2757	10	6.02	L-ornithine 5-monooxygenase

Ben3g2800	4	5.99	hypothetical protein
Ben3g6898	118	5.99	glycoside hydrolase family 3 protein
Ben3g4242	67	5.88	endo-polygalacturonase PG1
Ben3g3249	70	5.87	cysteine-rich secretory family protein
Ben3g189	13	5.87	lysine N-acyltransferase, putative
Ben3g7637	64	5.79	glycoside hydrolase family 10 protein
Ben3g251	140	5.78	haloacid dehalogenase-like hydrolase
Ben3g7364	118	5.76	glycoside hydrolase family 62 protein
Ben3g2601	3	5.75	Benzoate 4-monooxygenase
Ben3g2639	33	5.73	aromatic peroxygenase
Ben3g935	333	5.72	peptidyl-Lys metalloendopeptidase
Ben3g6910	51	5.70	putative iron reductase
Ben3g6962	321	5.65	putative 1,4-beta-D-glucan cellobiohydrolase B
Ben3g11300	6	5.65	hypothetical protein
Ben3g4865	9	5.63	pectate lyase, putative
Ben3g10605	13	5.62	GDSL-like lipase/acylhydrolase
Ben3g10264	17	5.60	glycoside hydrolase family 45 protein
Ben3g8520	21	5.59	carboxylesterase
Ben3g5658	43	5.56	glycoside hydrolase family 61 protein
Ben3g7512	82	5.54	hypothetical protein
Ben3g8536	19	5.54	hypothetical protein
Ben3g9675	60	5.51	cytochrome P450 family monooxygenase
Ben3g8173	19	5.51	chitin-binding domain protein
Ben3g1311	13	5.50	arabinan endo-1,5-alpha-L-arabinosidase
Ben3g1281	92	5.48	alpha/beta hydrolase family containing protein
Ben3g7612	110	5.48	putative gEgh 16 protein
Ben3g7678	519	5.43	cuticle-degrading protease
Ben3g8214	405	5.42	transmembrane protein, putative
Ben3g3590	2	5.42	cutinase
Ben3g9507	34	5.42	peptidyl-Lys metalloendopeptidase
Ben3g2070	127	5.39	extracellular metalloprotease
Ben3g6249	38	5.32	RTA1-domain protein
Ben3g12402	2	5.30	hypothetical protein
Ben3g9854	3	5.28	Extracellular metalloprotease 1
Ben3g7020	109	5.27	mandelate racemase/muconate lactonizing enzyme family protein
Ben3g3232	4	5.25	glycoside hydrolase family 61 protein
Ben3g2996	84	5.23	oxaloacetate acetylhydrolase
Ben3g11267	1	5.23	peptidyl-lys metalloendopeptidase
Ben3g8400	24	5.22	glycoside hydrolase family 5 protein
Ben3g7263	4	5.22	putative pectate lyase
Ben3g3031	44	5.22	cell wall glycosyl hydrolase, putative
Ben3g3400	7	5.21	phosphotransferase enzyme family protein
Ben3g2821	43	5.19	citrate synthase
Ben3g8213	22	5.18	transmembrane protein, putative
Ben3g1421	34	5.17	hypothetical protein
Ben3g11298	3	5.16	hypothetical protein
Ben3g9506	341	5.16	deuterolysin metalloprotease (M35) family containing protein
Ben3g4089	24	5.15	glycoside hydrolase family 61 protein
Ben3g6269	16	5.12	Guanyl-specific ribonuclease F1
Ben3g7821	57	5.11	glycoside hydrolase family 61 protein
Ben3g12096	2	5.10	hypothetical protein
Ben3g8824	406	5.10	trehalose utilization protein
Ben3g4243	83	5.09	endo-polygalacturonase PG1
Ben3g11054	273	5.09	glycoside hydrolase family 61 protein
Ben3g11935	4	5.08	zinc-binding oxidoreductase ToxD, putative
Ben3g8490	10	5.04	glycoside hydrolase family 61 protein
Ben3g5065	51	5.03	pectate lyase
Ben3g6323	8	5.03	glycoside hydrolase family 18 and carbohydrate-binding 5
Ben3g6829	124	5.02	alpha-L-rhamnosidase, partial
Ben3g1351	6	4.99	putative pectinesterase A
Ben3g4463	131	4.98	gEgh 16 protein, putative
Ben3g5302	10	4.92	pectin lyase F, putative
Ben3g4251	247	4.89	glycoside hydrolase family 16 protein
Ben3g4874	3	4.89	adenylosuccinate lyase
Ben3g9386	41	4.89	alpha/beta hydrolase family containing protein
Ben3g4134	73	4.87	endo-1,4-beta-xylanase

**Ben3g6715**	1163	4.82	extracellular metalloproteinase MEP
**Ben3g9518**	4	4.82	hypothetical protein
**Ben3g537**	77	4.81	DUF3129 family protein
Ben3g578	101	4.79	PHB depolymerase family esterase
Ben3g860	20	4.78	glycoside hydrolase family 61 protein
**Ben3g4060**	233	4.75	glycoside hydrolase family 61 protein
**Ben3g12106**	1	4.74	hypothetical protein
**Ben3g3286**	9	4.71	alpha/beta hydrolase fold protein
**Ben3g4864**	6	4.71	pectate lyase, putative
**Ben3g4688**	19	4.71	peptidase family S41 domain protein
**Ben3g9120**	46	4.70	guanyl-specific ribonuclease F1
**Ben3g7019**	93	4.70	MFS sugar transporter, putative
Ben3g7617	164	4.68	putative gEgh 16 protein
**Ben3g7024**	148	4.68	transmembrane protein, putative
**Ben3g2636**	302	4.67	pleiotropic drug resistance ABC transporter
**Ben3g10009**	16	4.67	peptidase family S41 domain protein
**Ben3g12073**	9	4.66	DDE family endonuclease
**Ben3g2524**	4	4.65	cytochrome P450 family protein
Ben3g3004	105	4.65	potassium/sodium efflux P-type ATPase
**Ben3g7380**	5	4.64	hypothetical protein
**Ben3g7379**	17	4.63	endo-polygalacturonase PG1
**Ben3g7036**	4	4.61	sugar transporter
**Ben3g7563**	52	4.61	Phosphatidylinositol 3-kinase VPS34
Ben3g4270	2	4.60	lipase from carbohydrate esterase family CE10 protein, putative
Ben3g5054	104	4.58	mannan endo-1,4-beta-mannosidase
**Ben3g4462**	81	4.57	hypothetical protein
**Ben3g2953**	56	4.53	hypothetical protein
**Ben3g2705**	14	4.53	sugar porter (SP) family MFS transporter
Ben3g6723	3	4.52	pectate lyase, putative
Ben3g4700	243	4.50	alpha-amylase domain protein
**Ben3g5353**	6	4.50	putative cyclin-dependent kinase 9
**Ben3g3871**	88	4.49	nonribosomal peptide synthetase
**Ben3g3048**	6	4.47	GDSL-like lipase/acylhydrolase
**Ben3g4274**	103	4.45	hypothetical protein
**Ben3g2663**	290	4.44	amino acid transporter
**Ben3g7068**	1	4.40	NADPH-dependent methylglyoxal reductase GRE2
**Ben3g7035**	3	4.40	sugar transporter
**Ben3g2756**	1	4.38	L-ornithine 5-monooxygenase
**Ben3g9725**	69	4.38	isoamyl alcohol oxidase, putative
Ben3g8505	44	4.35	glycoside hydrolase family 61 protein
Ben3g5145	77	4.35	transmembrane protein, putative
**Ben3g9248**	2	4.34	NADH:flavin oxidoreductase/NADH oxidase
**Ben3g2183**	72	4.33	glycoside hydrolase family 31 protein
Ben3g12206	75	4.32	DDE family endonuclease
**Ben3g8017**	6	4.31	retrotransposon gag protein
**Ben3g3119**	84	4.31	alpha-L-arabinofuranosidase
**Ben3g7510**	65	4.28	zinc-binding dehydrogenase
**Ben3g5101**	25	4.28	hypothetical protein
**Ben3g2370**	1	4.28	glutathione S-transferase-like protein, putative
**Ben3g79**	118	4.26	pleiotropic drug resistance ABC transporter
**Ben3g8966**	1	4.26	hypothetical protein
**Ben3g11003**	29	4.26	GDSL-like lipase acylhydrolase
Ben3g12036	39	4.25	hypothetical protein
**Ben3g9897**	149	4.25	aminopeptidase Y
**Ben3g815**	1	4.21	cytidine and deoxycytidylate deaminase zinc-binding region protein
**Ben3g5192**	16	4.21	concanamycin induced protein C CipC1 protein, putative
**Ben3g732**	26	4.20	hypothetical protein
**Ben3g8927**	5	4.19	putative transmembrane protein
**Ben3g10325**	28	4.18	cytochrome P450 family oxidoreductase
**Ben3g9490**	73	4.18	cytochrome P450 family oxidoreductase
**Ben3g2096**	27	4.18	MFS polyamine transporter
**Ben3g3051**	16	4.18	laccase, multicopper oxidase, benzenediol:oxygen oxidorectuctase
Ben3g8750	2	4.17	hypothetical protein
**Ben3g9489**	16	4.16	cytochrome P450 family oxidoreductase
**Ben3g12315**	36	4.16	hypothetical protein
Ben3g248	97	4.15	hypothetical protein

Ben3g11071	17	4.15	hypothetical protein
Ben3g3347	104	4.14	tyrosinase tyrosinase: common central domain protein
Ben3g5094	42	4.13	acetyltransferase (GNAT) family containing protein
Ben3g4881	4	4.13	pectate lyase, putative
Ben3g2443	42	4.13	xylitol dehydrogenase
Ben3g2801	1	4.12	glutathione transferase, putative
Ben3g6204	49	4.11	putative endo-beta-1,4-glucanase D
Ben3g3500	13	4.11	pectate lyase, putative
Ben3g5280	8	4.10	pectin methylesterase family protein
Ben3g8649	4	4.09	glycoside hydrolase family 61 protein
Ben3g5034	22	4.09	pectate lyase
Ben3g6201	2	4.08	putative pectinesterase A
Ben3g7808	1	4.07	hypothetical protein
Ben3g12499	48	4.07	exopolygalacturonase, putative
Ben3g23	2	4.04	tigger transposable element-derived protein
Ben3g3950	2	4.02	major intrinsic protein (MIP) family transporter
Ben3g9387	642	4.00	alpha/beta hydrolase family containing protein
Ben3g4824	101	3.99	1,4-beta-D-glucan cellobiohydrolase
Ben3g2285	113	3.97	hypothetical protein
Ben3g10189	4	3.97	Glutamate receptor 3,4
Ben3g12385	1	3.97	hypothetical protein
Ben3g12329	4	3.97	hypothetical protein
Ben3g4799	11	3.96	transmembrane protein, putative
Ben3g4123	25	3.96	cryptococcal mannosyltransferase
Ben3g3308	2	3.94	glutathione S-transferase
Ben3g7073	22	3.93	feruloyl esterase B
Ben3g8650	136	3.93	glycoside hydrolase family 61 protein, putative
Ben3g11957	7	3.92	HAT family dimerization protein
Ben3g29	183	3.90	pectate lyase
Ben3g6322	50	3.88	tyrosinase tyrosinase: common central domain protein
Ben3g4441	8	3.88	glycoside hydrolase family 5 protein
Ben3g4176	3	3.87	DUF3294 family protein
Ben3g190	22	3.85	siderophore iron transporter 3
Ben3g7687	72	3.84	bacterial leucyl aminopeptidase
Ben3g4052	3	3.84	pectate lyase
Ben3g8430	240	3.80	aminopeptidase
Ben3g4759	114	3.80	glucan 1,3-beta-glucosidase
Ben3g6170	3	3.79	hypothetical protein
Ben3g9413	1	3.77	hypothetical protein
Ben3g5375	6	3.76	AMP-binding enzyme
Ben3g6417	24	3.75	arabinogalactan endo-1,4-beta-galactosidase
Ben3g12497	3	3.75	transmembrane protein, putative
Ben3g3019	33	3.75	Cutinase
Ben3g8259	4	3.75	hypothetical protein
Ben3g8325	12	3.75	putative peptide transporter ptr2
Ben3g7241	43	3.74	short chain dehydrogenase
Ben3g6916	7	3.74	hypothetical protein
Ben3g6930	2	3.73	protocatechuate-dioxygenase beta subunit protein, putative
Ben3g6533	38	3.73	carbohydrate-binding module family 1 protein
Ben3g6713	14	3.72	extracellular metalloproteinase MEP
Ben3g9112	4	3.72	FMN-dependent dehydrogenase
Ben3g7467	3	3.72	alcohol dehydrogenase zinc-binding domain protein
Ben3g12353	88	3.71	hypothetical protein
Ben3g1322	49	3.71	putative transmembrane protein
Ben3g4772	334	3.69	transmembrane protein, putative
Ben3g4739	3	3.69	endo-polygalacturonase PG1
Ben3g1691	14	3.67	glycoside hydrolase family 3 protein
Ben3g8399	80	3.66	carboxypeptidase a2
Ben3g7541	2	3.66	glycoside hydrolase family 61 protein
Ben3g2161	34	3.66	endoplasmic reticulum protein
Ben3g7799	35	3.65	homeobox domain protein
Ben3g4220	20	3.65	tyrosinase tyrosinase: common central domain protein
Ben3g10404	5	3.65	alcohol dehydrogenase zinc-binding domain protein
Ben3g5944	17	3.62	glycoside hydrolase family 35 protein
Ben3g6225	1	3.59	hypothetical protein
Ben3g3868	86	3.58	cell wall glycosyl hydrolase YteR

Ben3g4033	13	3.58	alpha-ketoglutarate-dependent taurine dioxygenase
Ben3g1988	25	3.57	putative MFS multidrug resistance transporter
Ben3g10948	3	3.54	hAT family dimerization protein
Ben3g8452	96	3.54	lipoic acid synthetase
Ben3g11519	1	3.54	DDE family endonuclease
Ben3g2791	3	3.53	glycoside hydrolase family 13 protein
Ben3g24	1	3.53	tigger transposable element-derived protein
Ben3g2952	42	3.53	O-methylsterigmatocystin oxidoreductase
Ben3g5635	16	3.53	GDSL-like lipase/acylhydrolase
Ben3g710	37	3.52	peptidyl-Lys metalloendopeptidase
Ben3g9730	8	3.51	endo-1,4-beta-xylanase
Ben3g6321	19	3.50	tyrosinase tyrosinase: common central domain protein
Ben3g797	160	3.49	cellobiohydrolase II
Ben3g7444	1	3.48	1,4-alpha-glucan branching enzyme GlgB 2
Ben3g7864	2	3.47	jacalin-like lectin domain protein, putative
Ben3g760	6	3.46	related to ASD-1 rhamnogalacturonase B precursor
Ben3g6077	19	3.46	GDSL lipase/acylhydrolase family protein
Ben3g7735	172	3.45	peptidase family s41 domain protein
Ben3g3312	35	3.44	laccase, multicopper oxidase, benzenediol:oxygen oxidorectuctase
Ben3g8926	36	3.44	fasciclin domain protein
Ben3g7077	1	3.42	CFEM domain protein
Ben3g9438	36	3.42	rhamnogalacturonate lyase
Ben3g9057	4	3.42	putative GNAT family acetyltransferase
Ben3g11689	81	3.41	glycoside hydrolase family 13 protein
Ben3g3189	10	3.41	glycosyl hydrolase family 10 protein
Ben3g1716	1	3.40	Putative AC transposase
Ben3g8715	6	3.40	xyloglucan-specific endo-beta-1,4-glucanase A
Ben3g4814	2	3.40	hypothetical protein
Ben3g11437	12	3.39	GDSL-like lipase/acylhydrolase domain protein
Ben3g8630	95	3.36	pectate lyase
Ben3g5191	88	3.36	CipC protein, putative
Ben3g8746	143	3.35	putative glycoside hydrolase family 43 protein
Ben3g2539	4	3.35	NADP-dependent alcohol dehydrogenase
Ben3g7384	84	3.34	GDSL-like lipase/acylhydrolase domain protein
Ben3g1313	181	3.34	glycoside hydrolase family 16 protein
Ben3g12556	3	3.34	hypothetical protein
Ben3g1829	2	3.33	COP8 protein
Ben3g2341	133	3.32	hypothetical protein
Ben3g2638	72	3.32	aromatic peroxygenase
Ben3g7116	16	3.31	carboxylesterase
Ben3g6660	2	3.31	tyrosinase tyrosinase: common central domain protein
Ben3g10783	166	3.30	glycoside hydrolase family protein
Ben3g2447	10	3.30	carbohydrate-binding domain protein, putative
Ben3g2502	42	3.30	putative transmembrane protein
Ben3g10100	36	3.30	endonuclease/exonuclease/phosphatase family protein
Ben3g9185	11	3.30	glycoside hydrolase family 61 protein
Ben3g2085	22	3.30	glycoside hydrolase family 92 protein
Ben3g7942	6	3.30	pyranose 2-oxidase
Ben3g3267	32	3.29	epoxide hydrolase
Ben3g31	192	3.29	pectate lyase
Ben3g3569	45	3.29	MUC1 extracellular alpha-1,4-glucan glucosidase-like protein
Ben3g12545	3	3.28	putative Ty3/Gypsy polyprotein/retrotransposon
Ben3g10096	2	3.28	hypothetical protein
Ben3g2501	2	3.27	hypothetical protein
Ben3g6731	24	3.27	Plasma membrane proteolipid 3
Ben3g701	179	3.26	dihydrodipicolinate synthetase family protein
Ben3g4330	46	3.25	putative ethanolamine utilization protein (EutQ)
Ben3g4773	1124	3.25	transmembrane protein, putative
Ben3g2647	13	3.25	trimethyllysine dioxygenase
Ben3g3501	22	3.25	pectate lyase, putative
Ben3g2824	25	3.24	glycoside hydrolase family 5 protein
Ben3g9140	173	3.22	extracellular metalloproteinase MEP
Ben3g10227	76	3.22	tyrosinase tyrosinase: common central domain protein
Ben3g4628	11	3.22	F-box-like domain protein
Ben3g3490	10	3.22	putative pectate lyase
Ben3g9980	133	3.22	hexose transporter

Ben3g9393	203	3.20	MFS sugar transporter, putative
Ben3g8872	114	3.20	protoglobin protein
Ben3g7230	1	3.19	alpha/beta hydrolase fold protein
Ben3g9493	38	3.19	hypothetical protein
Ben3g8483	1	3.17	deuterolysin metalloprotease (M35) family containing protein
Ben3g7326	144	3.17	delta(24(24(1)))-sterol reductase
Ben3g982	13	3.17	hypothetical protein
Ben3g10258	1	3.16	transmembrane protein, putative
Ben3g733	34	3.16	conidiation protein
Ben3g5283	1	3.16	rhamnogalacturonate lyase
Ben3g7322	45	3.16	triacylglycerol lipase
Ben3g6491	1	3.15	pectate lyase domain-containing protein
Ben3g8671	24	3.15	methyltransferase FkbM domain protein
Ben3g10922	1	3.14	heat shock protein
Ben3g10213	3	3.14	helix loop helix DNA-binding domain protein
Ben3g6771	19	3.13	putative mannan endo-1,4-beta-mannosidase
Ben3g9363	1	3.13	hAT family dimerization protein
Ben3g7881	3	3.13	pectinesterase
Ben3g8879	111	3.12	MFS general substrate transporter
Ben3g12114	10	3.12	hAT family dimerization protein
Ben3g12037	1	3.11	cytochrome P450 family protein
Ben3g4735	27	3.11	dihydrodipicolinate synthetase family protein
Ben3g4240	22	3.11	endo-polygalacturonase PG1
Ben3g8595	35	3.10	hypothetical protein
Ben3g7749	3	3.10	3-ketoacyl-(acyl-carrier) reductase
Ben3g101	42	3.09	glycoside hydrolase family 28 protein
Ben3g9272	25	3.09	DUF1349 family protein
Ben3g7252	70	3.09	xanthine/uracil permease C887, putative
Ben3g5921	22	3.09	sterol 24-C-methyltransferase Erg6
Ben3g5516	3	3.08	Monocarboxylate transporter 12
Ben3g4483	10	3.07	WD domain, G-beta repeat protein
Ben3g12151	38	3.07	glycoside hydrolase family protein
Ben3g11982	5	3.07	hypothetical protein
Ben3g10275	51	3.07	Tc5 transposase DNA-binding domain protein
Ben3g12507	3	3.06	DEAD/DEAH-box helicase
Ben3g6074	31	3.06	polysaccharide lyase family 4 protein
Ben3g10614	0	3.04	putative Transposon Tf2-1 polyprotein
Ben3g9442	153	3.03	thioesterase family protein
Ben3g8183	29	3.02	RNA 3'-terminal phosphate cyclase-like protein
Ben3g12446	4	3.02	C2H2-type zinc-finger protein
Ben3g10405	1	3.02	rhamnogalacturonate lyase
Ben3g4275	39	3.01	rare lipoprotein A-like double-psi beta-barrel protein
Ben3g5439	64	3.01	GPI-anchored domain-containing protein
Ben3g2508	63	3.00	amino acid transporter
Ben3g5686	340	3.00	hypothetical protein
Ben3g7920	9	2.99	putative transmembrane protein
Ben3g6979	115	2.99	six-hairpin glycosidase-like protein, putative
Ben3g7836	55	2.99	glycoside hydrolase family 27 protein
Ben3g9145	3	2.99	NADH:flavin oxidoreductase/NADH oxidase
Ben3g6767	13	2.97	solute symporter family transporter
Ben3g12567	3	2.97	tyrosine kinase family catalytic domain protein
Ben3g2881	7	2.97	MFS general substrate transporter
Ben3g5836	42	2.96	C2H2 domain-containing protein
Ben3g10156	160	2.96	ABC family B (MDR/TAP),protein
Ben3g2977	39	2.95	putative dephospho-CoA kinase
Ben3g3824	269	2.95	chitin deacetylase
Ben3g5851	152	2.94	glycoside hydrolase family 3 protein
Ben3g1278	17	2.94	lysozyme M1 (1,4-beta-N-acetylmuramidase) protein
Ben3g10263	2	2.93	Putative endoglucanase type K
Ben3g1546	428	2.93	OPT oligopeptide transporter
Ben3g3659	222	2.93	L-serine dehydratase
Ben3g5104	32	2.93	glycoside hydrolase family 61 protein
Ben3g1301	369	2.93	transmembrane protein, putative
Ben3g4342	29	2.93	glutathione S-transferase, putative
Ben3g1689	45	2.93	pectate lyase
Ben3g3571	1	2.92	endo-polygalacturonase, partial

Ben3g2880	46	2.92	MFS general substrate transporter
Ben3g7540	20	2.91	glycoside hydrolase family 61 protein
Ben3g6820	31	2.91	choline dehydrogenase
Ben3g8539	11	2.91	phytanoyl-CoA dioxygenase
Ben3g1658	51	2.90	putative glycosidase C21B10,07
Ben3g10343	1	2.90	alcohol dehydrogenase zinc-binding domain protein
Ben3g8563	235	2.89	chitin deacetylase
Ben3g6266	158	2.88	MFS transporter, FHS family, L-fucose permease
Ben3g1256	11	2.88	pectin lyase, putative
Ben3g4076	110	2.88	hypothetical protein
Ben3g572	5	2.87	glutathione S-transferase carboxy-terminal-like protein
Ben3g9398	10	2.87	ICE-like protease (caspase) p20 domain protein
Ben3g5367	11	2.86	UDP-glycosyltransferase 74C1
Ben3g6444	1	2.85	transmembrane protein, putative
Ben3g8621	28	2.85	allergen protein
Ben3g188	0	2.85	GNAT family acetyltransferase
Ben3g4393	15	2.84	MFS sugar transporter-like protein
Ben3g12004	9	2.83	Retrovirus-related Pol polyprotein from transposon TNT 1-94
Ben3g6197	2	2.83	F-box-like domain protein
Ben3g2309	52	2.83	transmembrane protein, putative
Ben3g10912	0	2.82	hypothetical protein
Ben3g7009	35	2.81	phosphoadenosine phosphosulfate reductase
Ben3g6835	53	2.81	mannoprotein
Ben3g6162	1	2.80	arabinan endo-1,5-alpha-L-arabinosidase
Ben3g7173	17	2.78	cuticle-degrading protease, putative
Ben3g1621	5	2.77	putative indole-diterpene biosynthesis protein PaxU
Ben3g5910	2	2.77	trehalose utilization protein
Ben3g8522	85	2.77	MFS transporter
Ben3g364	27	2.77	RNA exonuclease 4
Ben3g9271	46	2.77	glycoside hydrolase family 6 protein
Ben3g12445	1	2.76	Dynactin subunit 1
Ben3g6905	20	2.76	L-lysine 6-monooxygenase (NADPH-requiring) protein
Ben3g4854	50	2.75	glycoside hydrolase family 5 protein
Ben3g346	367	2.74	2-nitropropane dioxygenase
Ben3g8379	86	2.74	CVNH domain protein
Ben3g2549	225	2.74	hypothetical protein
Ben3g4813	113	2.74	hypothetical protein
Ben3g9873	4	2.73	hypothetical protein
Ben3g1751	75	2.72	tripeptidyl-peptidase I
Ben3g4624	1	2.72	fungal cellulose binding domain-containing protein
Ben3g2547	32	2.72	hypothetical protein
Ben3g1575	15	2.72	MFS transporter
Ben3g1700	13	2.71	glycoside hydrolase catalytic core protein
Ben3g5859	61	2.71	beta-glucosidase, glycoside hydrolase family 3 protein
Ben3g7044	2	2.71	pathogenesis-related protein PR5K (thaumatin family)
Ben3g5824	103	2.70	hypothetical protein
Ben3g425	1	2.70	glycopeptide protein
Ben3g7381	1	2.70	hypothetical protein
Ben3g12549	24	2.69	glycoside hydrolase family protein
Ben3g3224	67	2.69	feruloyl esterase B
Ben3g5424	1	2.69	hypothetical protein
Ben3g858	3	2.69	peptidoglycan-binding domain 1 protein, putative
Ben3g3298	3	2.69	Dynactin subunit 1
Ben3g12330	2	2.67	DDE family endonuclease
Ben3g2916	137	2.66	lactonase, 7-bladed beta-propeller
Ben3g8525	15	2.66	glycosyltransferase family 8 protein
Ben3g7475	0	2.65	alcohol dehydrogenase zinc-binding domain protein
Ben3g7842	75	2.65	NADH:flavin oxidoreductase
Ben3g10407	101	2.64	hypothetical protein
Ben3g5900	230	2.64	fungal specific transcription factor domain protein
Ben3g7240	1	2.64	transmembrane protein-like protein C30D11,06c
Ben3g7243	42	2.63	hypothetical protein
Ben3g10169	5	2.63	hypothetical protein
Ben3g8112	1	2.61	isoflavone reductase-like protein
Ben3g5111	17	2.61	UPF0743 protein
Ben3g4656	7	2.60	cytochrome P450 family protein

Ben3g12196	7	2.60	hAT family dimerization protein
Ben3g2522	10	2.60	40S ribosomal protein S14, putative
Ben3g7333	1	2.60	hypothetical protein
Ben3g3511	30	2.58	glycoside hydrolase family 27 protein
Ben3g8833	9	2.58	glycoside hydrolase family 61 protein
Ben3g6875	122	2.58	putative 1,4-beta-D-glucan cellobiohydrolase B
Ben3g9767	2	2.57	Phosphatidylinositol 3-kinase VPS34
Ben3g4786	30	2.57	Lipase
Ben3g10297	233	2.57	Rgp1 domain protein, putative
Ben3g10660	164	2.57	glutamate dehydrogenase (NADP+)
Ben3g11597	1	2.56	hypothetical protein
Ben3g191	12	2.55	siderophore iron transporter 3
Ben3g7570	16	2.55	methyltransferase domain protein, putative
Ben3g7949	4	2.55	hypothetical protein
Ben3g1140	79	2.54	D-galacturonic acid reductase
Ben3g8494	23	2.54	MFS general substrate transporter
Ben3g7260	51	2.54	hypothetical protein
Ben3g1375	42	2.53	hypothetical protein
Ben3g9137	4	2.53	hypothetical protein
Ben3g8760	5	2.52	GDSL-like lipase/acylhydrolase
Ben3g5030	4	2.52	endo-polygalacturonase PG1
Ben3g5563	4	2.52	fruiting body protein Sc7
Ben3g5976	1	2.52	GDSL-like lipase/acylhydrolase
Ben3g5028	54	2.52	pectin lyase
Ben3g1260	67	2.52	glycoside hydrolase family 16 protein
Ben3g2808	114	2.51	aldo/keto reductase family protein
Ben3g4043	128	2.51	MFS lactose permease, putative
Ben3g3872	14	2.51	enoyl-CoA hydratase
Ben3g2544	20	2.50	DSBA domain-containing protein
Ben3g2058	18	2.50	general amino acid permease (AGP2), putative
Ben3g6076	4	2.49	GDSL-like lipase/acylhydrolase
Ben3g12484	0	2.49	tyrosinase tyrosinase: common central domain protein
Ben3g104	4	2.48	hypothetical protein
Ben3g8367	380	2.48	hypothetical protein
Ben3g10860	0	2.48	hypothetical protein
Ben3g4369	2	2.48	APC amino acid permease
Ben3g9670	108	2.48	Minor extracellular protease vpr
Ben3g975	564	2.47	alpha-1,3-glucan synthase
Ben3g5297	379	2.46	polysaccharide lyase family 8 protein
Ben3g2536	17	2.46	glycoside hydrolase catalytic core protein
Ben3g8574	66	2.46	alkaline phosphatase
Ben3g3882	152	2.46	aldo/keto reductase
Ben3g3886	8	2.46	phosphoglycerate mutase family protein
Ben3g1402	193	2.46	1,4-alpha-glucan branching enzyme
Ben3g9426	190	2.45	deuterolysin metalloprotease (M35) family containing protein
Ben3g6886	16	2.45	NADH flavin oxidoreductase/NADH oxidase
Ben3g7064	25	2.44	C4-dicarboxylate transporter/malic acid transporter
Ben3g2930	91	2.44	hypothetical protein
Ben3g6912	64	2.44	peroxisomal 2,4-dienoyl-CoA reductase
Ben3g12356	3	2.44	tyrosinase tyrosinase: common central domain protein
Ben3g796	1689	2.43	delta 9-fatty acid desaturase
Ben3g6897	62	2.43	hypothetical protein
Ben3g2503	54	2.43	GMC oxidoreductase
Ben3g7567	2	2.43	MFS general substrate transporter
Ben3g2012	51	2.43	cellobiose dehydrogenase
Ben3g62	50	2.43	ABC transporter, Pdr11p
Ben3g9797	2	2.42	putative serine-rich protein C13G6,10c
Ben3g9437	7	2.42	polysaccharide lyase family 4 protein
Ben3g7766	30	2.42	glycoside hydrolase family 1 protein
Ben3g6920	4	2.41	nucleoside diphosphate kinase
Ben3g8707	21	2.41	glycoside hydrolase family 43 protein
Ben3g1160	3	2.41	MFS transporter
Ben3g6497	11	2.40	hypothetical protein
Ben3g3064	3	2.40	basic region leucine zipper protein
Ben3g7995	1	2.40	4-oxalocrotonate tautomerase
Ben3g1580	110	2.40	cytochrome P450 family protein

Ben3g5487	11	2.39	hypothetical protein
Ben3g6678	6	2.39	FIP1 motif protein
Ben3g3486	187	2.38	NAD(P)H-dependent D-xylose reductase xyl1, putative
Ben3g7809	2	2.37	hypothetical protein
Ben3g5198	297	2.37	thioredoxin reductase (NADPH)
Ben3g9566	6	2.37	H-type lectin domain protein
Ben3g2298	15	2.36	D-tyrosyl-tRNA(Tyr) deacylase
Ben3g9485	218	2.36	hypothetical protein
Ben3g3107	3	2.36	beta-1,4-mannosyl-glycoprotein beta-1,4-N-acetylglucosaminyltransferase
Ben3g9593	172	2.35	sugar porter (SP) family MFS transporter
Ben3g7317	36	2.35	Lipase
Ben3g249	131	2.35	hypothetical protein
Ben3g7180	74	2.35	fungal specific transcription factor domain protein
Ben3g5878	204	2.34	hypothetical protein
Ben3g6325	146	2.34	1,4-beta-D-glucan cellobiohydrolase B, putative
Ben3g2013	209	2.34	cytoplasmic tRNA 2-thiolation-like protein
Ben3g3221	1	2.33	WSC domain protein
Ben3g10561	10	2.32	WD repeat-containing protein JIP5
Ben3g11831	67	2.32	putative MFS-type transporter C409,08
Ben3g5665	121	2.32	small conductance mechanosensitive ion channel (MscS) family protein
Ben3g8263	0	2.32	hAT family dimerization protein
Ben3g1203	51	2.32	3-oxoacyl-(acyl-carrier) reductase
Ben3g5974	7	2.31	hypothetical protein
Ben3g11079	29	2.31	cuticle-degrading protease
Ben3g4913	188	2.31	putative glycoside hydrolase family 43 protein
Ben3g11417	1	2.31	SCO-spondin
Ben3g5468	137	2.30	chitin-binding domain protein
Ben3g3503	2	2.30	N-acyl homoserine lactonase AttM
Ben3g4291	69	2.30	AhpC/TSA antioxidant enzyme
Ben3g3367	14	2.30	cysteine-rich secretory family protein
Ben3g1805	100	2.30	D-arabinitol 2-dehydrogenase
Ben3g2858	72	2.30	FAD-dependent oxidoreductase, putative
Ben3g4841	3	2.30	3-beta hydroxysteroid dehydrogenase/isomerase family protein
Ben3g11083	4	2.29	putative 1,4-beta-D-glucan cellobiohydrolase B
Ben3g11686	27	2.29	endo-1,4-beta-xylanase, partial
Ben3g11544	43	2.29	PII uridylyl-transferase
Ben3g2145	43	2.29	hypothetical protein
Ben3g1215	27	2.29	hypothetical protein
Ben3g2277	43	2.28	glycoside hydrolase family 9 protein
Ben3g10943	252	2.28	hypothetical protein
Ben3g1833	1	2.27	winged helix turn helix protein
Ben3g4065	1	2.27	FAD-linked oxidoreductase
Ben3g7577	92	2.27	fungal Zn(2)-cys(6) binuclear cluster domain protein
Ben3g7843	77	2.26	glycoside hydrolase family 74 protein
Ben3g4982	5	2.26	carbohydrate esterase family 16 protein, putative
Ben3g2264	53	2.25	PX domain protein
Ben3g6502	3	2.25	MFS general substrate transporter
Ben3g3570	49	2.24	mannosyltransferase domain protein
Ben3g4448	414	2.24	long-chain-fatty-acid-CoA ligase
Ben3g2767	7	2.24	lactonase, 7-bladed beta-propeller
Ben3g3043	21	2.24	FAD-dependent oxidoreductase
Ben3g6060	52	2.24	mannan endo-1,4-beta-mannosidase
Ben3g7900	103	2.23	glycoside hydrolase family 16 protein
Ben3g9977	3	2.23	glycoside hydrolase family 16 protein
Ben3g9296	4	2.23	bestrophin protein
Ben3g9388	237	2.23	thiamine biosynthesis protein
Ben3g1694	600	2.22	hypothetical protein
Ben3g8328	28	2.22	hypothetical protein
Ben3g5544	11	2.22	hypothetical protein
Ben3g3014	6	2.22	F-box-like domain protein
Ben3g4404	32	2.22	ammonium transporter
Ben3g1282	2	2.21	cytochrome P450 family oxidoreductase OrdA, putative
Ben3g3928	988	2.21	60S ribosomal protein L28
Ben3g4812	52	2.21	transmembrane protein, putative
Ben3g2590	133	2.21	citrate synthase
Ben3g8965	38	2.21	endonuclease/exonuclease/phosphatase family protein

Ben3g2618	21	2.21	peroxidase, putative
Ben3g9099	22	2.20	AAA domain protein
Ben3g3519	17	2.20	3-methyl-2-oxobutanoate hydroxymethyltransferase
Ben3g1229	38	2.19	glycoside hydrolase family 16 protein
Ben3g10539	19	2.19	hypothetical protein
Ben3g4981	22	2.18	Serine/Threonine kinase
Ben3g2972	10	2.17	aldo/keto reductase family protein
Ben3g10274	75	2.17	carbohydrate esterase family 4 protein
Ben3g5716	42	2.17	ZIP zinc/iron transport family protein
Ben3g233	35	2.16	O-methyltransferase, putative
Ben3g9338	2	2.16	hypothetical protein
Ben3g3853	30	2.16	myo-inositol oxygenase
Ben3g7981	2	2.16	hypothetical protein
Ben3g987	17	2.16	siderochrome-iron transporter Sit1 protein, putative
Ben3g2186	39	2.15	C6 transcription factor
Ben3g3	1	2.15	hypothetical protein
Ben3g2991	17	2.15	succinate/fumarate transporter
Ben3g9276	12	2.15	hypothetical protein
Ben3g2924	48	2.15	sexual differentiation process protein isp4, related protein
Ben3g2768	82	2.15	transmembrane protein, putative
Ben3g9568	1	2.15	H-type lectin domain protein
Ben3g4528	6	2.14	glycoside hydrolase family 43 protein
Ben3g2632	124	2.14	MFS monocarboxylate transporter, putative
Ben3g6159	545	2.14	OPT oligopeptide transporter
Ben3g2749	34	2.14	aminopeptidase Y, putative
Ben3g11280	1	2.14	hypothetical protein
Ben3g3284	96	2.14	MFS nicotinic acid transporter
Ben3g11825	6	2.14	glycosyl hydrolase family 10 domain-containing protein
Ben3g5152	11	2.13	3-ketoacyl-(acyl-carrier) reductase
Ben3g5834	154	2.13	2-nitropropane dioxygenase family protein, putative
Ben3g533	68	2.13	glycoside hydrolase family 3 protein
Ben3g4940	41	2.13	putative extracellular exo-polygalacturonase
Ben3g10105	26	2.12	NADH:flavin oxidoreductase/NADH oxidase
Ben3g7266	3	2.12	DJ-1/PfpI family protein
Ben3g4250	46	2.12	glycoside hydrolase family 16 protein
Ben3g11917	26	2.11	pathogenesis-related protein PR5K (thaumatin family)
Ben3g1738	5	2.11	glycoside hydrolase family 28 protein
Ben3g9018	2	2.11	S-adenosyl-L-methionine-dependent methyltransferase
Ben3g4121	1	2.11	putative transmembrane protein
Ben3g12312	15	2.11	Mip1 protein
Ben3g12053	30	2.11	endo-1,4-beta-xylanase
Ben3g10323	1	2.11	WW domain protein
Ben3g7620	33	2.10	homogentisate 1,2-dioxygenase
Ben3g2525	42	2.10	Ecl1 domain protein
Ben3g1212	28	2.10	3-oxoacyl-(acyl-carrier) reductase
Ben3g5819	139	2.10	C-5 sterol desaturase
Ben3g5833	218	2.10	ketol-acid reductoisomerase
Ben3g7287	9	2.09	DUF4598 domain protein
Ben3g1309	50	2.09	arabinan endo-1,5-alpha-L-arabinosidase
Ben3g6587	114	2.09	omega-6 fatty acid desaturase (delta-12 desaturase)
Ben3g2903	169	2.09	NADPH oxidase regulator NoxR
Ben3g5768	661	2.08	bZIP transcription factor domain-containing protein
Ben3g195	12	2.08	iron uptake cluster protein, putative
Ben3g2731	135	2.08	inorganic phosphate transporter
Ben3g11921	140	2.08	hypothetical protein
Ben3g70	25	2.08	hypothetical protein
Ben3g865	650	2.08	mannose-1-phosphate guanylyltransferase
Ben3g1469	60	2.07	glycosyl hydrolase family 88 protein
Ben3g8978	2	2.07	hypothetical protein
Ben3g4479	106	2.07	pria protein
Ben3g12259	5	2.07	hypothetical protein
Ben3g5628	122	2.06	enoyl-CoA hydratase/isomerase family protein
Ben3g7554	9	2.06	putative lipase domain protein
Ben3g802	635	2.06	plastocyanin-like domain protein
Ben3g11039	85	2.06	glycoside hydrolase family 79 protein
Ben3g6220	117	2.06	Frag1/DRAM/Sfk1 family protein

Ben3g8813	252	2.06	2-nitropropane dioxygenase
Ben3g7633	232	2.05	cytochrome P450 family 6 protein
Ben3g5155	41	2.05	putative xylosylphosphotransferase
Ben3g8936	3	2.05	hydrolase, putative
Ben3g11349	1	2.05	Protein CSF1
Ben3g10101	2	2.05	endonuclease/exonuclease/phosphatase family protein
Ben3g999	35	2.04	ribosome assembly protein
Ben3g9012	89	2.04	guanylate kinase
Ben3g10607	7	2.04	hypothetical protein
Ben3g2825	46	2.04	glycoside hydrolase family 5 protein
Ben3g699	421	2.04	UDP-galactose transporter Gms1
Ben3g4093	96	2.04	phthalate transporter
Ben3g6867	20	2.04	alpha-amylase-domain protein
Ben3g4523	22	2.04	hypothetical protein
Ben3g4361	17	2.03	acetamidase/formamidase
Ben3g6873	20	2.03	carboxyesterase-postia placenta Mad-698-R-like protein
Ben3g12163	46	2.03	H+/nucleoside cotransporter
Ben3g2162	120	2.02	Esterase
Ben3g1687	238	2.02	thiamine thiazole synthase
Ben3g7297	525	2.02	ATP-dependent RNA helicase ded1
Ben3g1414	5	2.02	hypothetical protein
Ben3g1893	29	2.02	aldo/keto reductase
Ben3g9479	13	2.02	hypothetical protein
Ben3g7671	2	2.02	cold-shock DNA-binding domain protein
Ben3g9502	20	2.02	deuterolysin metalloprotease (M35) family containing protein
Ben3g7845	77	2.01	nucleoside diphosphatase GDA1
Ben3g4201	7	2.01	putative pheromone-regulated membrane protein
Ben3g1365	119	2.01	hypothetical protein
Ben3g7619	20	2.01	FAD/NAD(P)-binding domain protein
Ben3g5159	229	2.01	transmembrane protein, putative
Ben3g2819	2	2.00	glycoside hydrolase family 76 protein
Ben3g10669	4	-2.00	hypothetical protein
Ben3g74	4	-2.00	hypothetical protein
Ben3g11472	12	-2.01	hypothetical protein
Ben3g8626	24	-2.01	hypothetical protein
Ben3g3168	19	-2.01	hypothetical protein
Ben3g10136	18	-2.02	response regulator receiver domain protein
Ben3g10	58	-2.02	hypothetical protein
Ben3g10519	3	-2.03	hypothetical protein
Ben3g1945	27	-2.03	hypothetical protein
Ben3g4810	26	-2.03	hypothetical protein
Ben3g2402	36	-2.03	hypothetical protein
Ben3g6605	2	-2.03	hypothetical protein
Ben3g8805	325	-2.03	hypothetical protein
Ben3g8970	12	-2.03	hypothetical protein
Ben3g7343	1	-2.03	F-box-like domain protein, putative
Ben3g3724	1	-2.03	hypothetical protein
Ben3g11499	3	-2.04	vegetative incompatibility protein HET-E-1
Ben3g6471	6	-2.04	hypothetical protein
Ben3g2324	46	-2.04	hypothetical protein
Ben3g11616	25	-2.04	hypothetical protein
Ben3g9993	29	-2.04	hypothetical protein
Ben3g3112	4	-2.05	hypothetical protein
Ben3g52	152	-2.05	Ras small GTPase family Ras protein
Ben3g9418	3	-2.06	aromatic di-alanine and TPR containing protein
Ben3g9002	32	-2.06	3-oxo-5-alpha-steroid 4-dehydrogenase
Ben3g9474	15	-2.06	hypothetical protein
Ben3g1092	185	-2.06	SGT1, suppressor of G2 allele of SKP1 protein, putative
Ben3g8734	18	-2.06	cytochrome P450 family protein
Ben3g9405	3	-2.07	hypothetical protein
Ben3g1834	9	-2.07	hypothetical protein
Ben3g7517	30	-2.08	F-box protein
Ben3g7798	11	-2.08	peptidase S9 prolyl oligopeptidase active site protein, putative
Ben3g7861	32	-2.08	hypothetical protein
Ben3g8054	6	-2.09	hypothetical protein
Ben3g4800	11	-2.09	hypothetical protein

Ben3g5880	3	-2.09	hypothetical protein
Ben3g5646	302	-2.09	hypothetical protein
Ben3g2900	9	-2.10	small nuclear ribonucleoprotein E
Ben3g9146	2335	-2.10	hypothetical protein
Ben3g8352	2076	-2.11	hypothetical protein
Ben3g4177	12	-2.11	hypothetical protein
Ben3g7757	3	-2.11	hypothetical protein
Ben3g6262	125	-2.11	UvrD-like helicase carboxy-terminal domain protein
Ben3g8336	26	-2.11	Tetracycline resistance protein from transposon Tn4351/Tn4400
Ben3g10906	1	-2.13	hypothetical protein
Ben3g7136	6	-2.13	hypothetical protein
Ben3g3891	17	-2.13	hypothetical protein
Ben3g1604	10	-2.14	hypothetical protein
Ben3g7923	48	-2.14	hypothetical protein
Ben3g5156	6	-2.14	hypothetical protein
Ben3g4749	1	-2.14	serine protease
Ben3g7858	7	-2.15	hypothetical protein
Ben3g9852	1	-2.15	hypothetical protein
Ben3g9713	5	-2.15	aromatic di-alanine and TPR containing protein
Ben3g1593	39	-2.16	putative maternal effect embryo arrest 18 protein
Ben3g11974	23	-2.16	calpain-1 catalytic subunit
Ben3g6374	5	-2.16	aromatic di-alanine and TPR containing protein
Ben3g9811	7	-2.16	transposase family Tnp2 protein
Ben3g2488	11	-2.17	aromatic di-alanine and TPR containing protein
Ben3g7536	67	-2.17	hypothetical protein
Ben3g12515	20	-2.17	hypothetical protein
Ben3g10331	55	-2.17	transitional endoplasmic reticulum ATPase
Ben3g11172	5	-2.17	putative vegetative incompatibility protein HET-E-1
Ben3g8611	105	-2.18	hypothetical protein
Ben3g9282	68	-2.18	hypothetical protein
Ben3g8089	34	-2.19	hypothetical protein
Ben3g10307	50	-2.20	hypothetical protein
Ben3g2160	50	-2.20	DUF2828 domain protein
Ben3g6538	81	-2.21	superoxide dismutase [Cu-Zn] protein
Ben3g1481	29	-2.22	hypothetical protein
Ben3g11855	1	-2.22	hypothetical protein
Ben3g9107	27	-2.22	cys/met metabolism PLP-dependent enzyme
Ben3g9468	6	-2.22	vegetative incompatibility protein HET-E-1
Ben3g7251	14	-2.22	hypothetical protein
Ben3g1004	88	-2.22	heat shock protein HSP104, putative
Ben3g7367	87	-2.23	hypothetical protein
Ben3g4717	56	-2.23	cytochrome P450 family protein
Ben3g9417	1	-2.23	hypothetical protein
Ben3g7122	188	-2.24	flavorubredoxin reductase
Ben3g1670	6	-2.24	hypothetical protein
Ben3g11138	7	-2.25	hypothetical protein
Ben3g5036	21	-2.25	cyclopropane-fatty-acyl-phospholipid synthase
Ben3g6522	331	-2.25	Dyp-type peroxidase
Ben3g8041	9	-2.25	hypothetical protein
Ben3g7220	27	-2.25	hypothetical protein
Ben3g9207	6	-2.26	hypothetical protein
Ben3g8632	18	-2.26	cytochrome P450 family protein
Ben3g10568	6	-2.26	hypothetical protein
Ben3g3018	13	-2.27	glycoside hydrolase family 18 protein
Ben3g8848	21	-2.27	hypothetical protein
Ben3g8006	54	-2.27	lipase class 3
Ben3g4213	6	-2.27	hypothetical protein
Ben3g6335	179	-2.27	COG4 transporter
Ben3g4335	6	-2.28	hypothetical protein
Ben3g8446	0	-2.28	hypothetical protein
Ben3g7898	2	-2.29	endonuclease/exonuclease/phosphatase family protein
Ben3g8816	0	-2.29	hypothetical protein
Ben3g4984	7	-2.29	hypothetical protein
Ben3g7472	0	-2.29	hypothetical protein
Ben3g8801	192	-2.30	hypothetical protein
Ben3g5957	9	-2.31	tyrosine kinase catalytic domain protein

Ben3g6603	1	-2.32	hypothetical protein
Ben3g6026	19	-2.32	hypothetical protein
Ben3g2912	26	-2.33	hypothetical protein
Ben3g6965	12	-2.33	hypothetical protein
Ben3g4730	15	-2.33	hypothetical protein
Ben3g9801	1	-2.33	hypothetical protein
Ben3g3479	1	-2.34	hypothetical protein
Ben3g4215	31	-2.34	hypothetical protein
Ben3g4130	12	-2.34	hypothetical protein
Ben3g10377	40	-2.34	hypothetical protein
Ben3g4372	0	-2.35	hypothetical protein
Ben3g8792	5	-2.35	hypothetical protein
Ben3g10083	212	-2.35	calpain-1 catalytic subunit
Ben3g5314	9	-2.35	aromatic di-alanine and TPR containing protein
Ben3g10581	240	-2.35	hypothetical protein
Ben3g12367	1	-2.36	hypothetical protein
Ben3g4298	2	-2.36	hypothetical protein
Ben3g10552	37	-2.37	hypothetical protein
Ben3g8800	1536	-2.37	hypothetical protein
Ben3g1818	93	-2.38	mannose-6-phosphate isomerase
Ben3g2661	33	-2.38	hypothetical protein
Ben3g12454	7	-2.38	cytochrome P450 family protein
Ben3g6559	112	-2.39	hypothetical protein
Ben3g7363	170	-2.39	von willebrand factor type A domain protein
Ben3g8991	217	-2.41	MFS transporter
Ben3g2984	14	-2.41	NADPH-dependent methylglyoxal reductase GRE2 protein
Ben3g3276	127	-2.41	hypothetical protein
Ben3g9745	1	-2.42	hypothetical protein
Ben3g3426	7	-2.43	hypothetical protein
Ben3g740	17	-2.43	hypothetical protein
Ben3g8925	17	-2.43	hypothetical protein
Ben3g7581	1	-2.44	tyrosine kinase domain protein
Ben3g11757	240	-2.46	hypothetical protein
Ben3g11670	4	-2.46	Pol polyprotein/retrotransposon, putative
Ben3g11715	7	-2.47	vegetative incompatibility protein HET-E-1
Ben3g8849	77	-2.47	hypothetical protein
Ben3g8901	13	-2.47	hypothetical protein
Ben3g11709	18	-2.48	hypothetical protein
Ben3g7250	13	-2.48	hypothetical protein
Ben3g12140	1	-2.49	hypothetical protein
Ben3g8616	1	-2.49	hypothetical protein
Ben3g7342	1	-2.49	hypothetical protein
Ben3g3352	295	-2.49	putative short-chain dehydrogenase/reductase
Ben3g2985	419	-2.49	NADPH-dependent methylglyoxal reductase GRE2 protein
Ben3g6936	141	-2.50	putative hydrolase
Ben3g12536	5	-2.51	3-ketoacyl-(acyl-carrier) reductase
Ben3g6950	1	-2.51	hypothetical protein
Ben3g9433	29	-2.52	hypothetical protein
Ben3g9994	8	-2.52	hypothetical protein
Ben3g9194	5	-2.53	hypothetical protein
Ben3g12344	63	-2.56	hypothetical protein
Ben3g8761	14	-2.56	hypothetical protein
Ben3g1732	8	-2.57	DEAD/DEAH-box helicase
Ben3g8772	2	-2.57	hypothetical protein
Ben3g10320	47	-2.59	3-ketoacyl-(acyl-carrier) reductase
Ben3g6311	7	-2.59	hypothetical protein
Ben3g12464	1	-2.61	hypothetical protein
Ben3g1710	8	-2.62	hypothetical protein
Ben3g7167	6	-2.63	hypothetical protein
Ben3g417	1	-2.64	putative NADPH-dependent methylglyoxal reductase GRE2
Ben3g5449	47	-2.65	membrane protein TVP38
Ben3g7652	1	-2.66	hypothetical protein
Ben3g10796	1	-2.68	hypothetical protein
Ben3g2649	55	-2.68	hypothetical protein
Ben3g9972	1	-2.68	hypothetical protein
Ben3g4753	1	-2.68	hypothetical protein

Ben3g3556	94	-2.69	hypothetical protein
Ben3g11552	2	-2.70	hypothetical protein
Ben3g12336	1	-2.71	kinesin light chain
Ben3g7137	1	-2.72	hypothetical protein
Ben3g5870	15	-2.72	glycoside hydrolase family 15 protein
Ben3g4934	127	-2.73	hypothetical protein
Ben3g9851	3	-2.73	tyrosine kinase catalytic domain protein
Ben3g4920	6	-2.78	hypothetical protein
Ben3g8948	940	-2.78	protein arginine N-methyltransferase 3
Ben3g8958	2	-2.79	hypothetical protein
Ben3g9695	17	-2.80	hypothetical protein
Ben3g12400	57	-2.80	hypothetical protein
Ben3g8012	41	-2.81	hypothetical protein
Ben3g524	21	-2.81	cyclopropane-fatty-acyl-phospholipid synthase
Ben3g11140	27	-2.85	hypothetical protein
Ben3g8588	2	-2.87	hypothetical protein
Ben3g9837	83	-2.88	hypothetical protein
Ben3g10138	8	-2.89	fungal zn(2)-cys(6) binuclear cluster domain protein, putative
Ben3g9367	35	-2.93	hypothetical protein
Ben3g2659	37	-2.94	permease, cytosine/purine, uracil, thiamine, allantoin protein
Ben3g8587	3	-2.95	hypothetical protein
Ben3g5993	2	-2.98	STE-like pheromone receptor
Ben3g8246	14	-2.98	hypothetical protein
Ben3g9368	3387	-2.98	hypothetical protein
Ben3g3015	11	-2.99	hypothetical protein
Ben3g10877	147	-2.99	Smr domain protein C11H11,03c
Ben3g3830	5	-2.99	hypothetical protein
Ben3g2806	32	-2.99	hypothetical protein
Ben3g9323	37	-3.01	hypothetical protein
Ben3g6772	113	-3.02	oxidoreductase, putative
Ben3g1637	788	-3.04	hypothetical protein
Ben3g8612	8	-3.08	hypothetical protein
Ben3g8613	370	-3.08	Dyp-type peroxidase
Ben3g9749	77	-3.10	SNF2 family amino-terminal protein
Ben3g5443	5	-3.12	hypothetical protein
Ben3g11169	7	-3.13	SNF2 family amino-terminal protein
Ben3g9835	32	-3.17	hypothetical protein
Ben3g6561	35	-3.19	hypothetical protein
Ben3g9373	4	-3.23	hypothetical protein
Ben3g9627	5	-3.25	hypothetical protein
Ben3g7779	142	-3.26	Dyp-type peroxidase
Ben3g12530	1	-3.30	hypothetical protein
Ben3g11893	22	-3.30	hypothetical protein
Ben3g6062	24	-3.32	hypothetical protein
Ben3g10636	26	-3.32	hypothetical protein
Ben3g10285	305	-3.33	phosphatidylserine decarboxylase
Ben3g5355	2	-3.33	hypothetical protein
Ben3g9260	143	-3.37	FAD-binding domain protein
Ben3g7031	869	-3.38	hemerythrin HHE cation-binding domain protein
Ben3g3436	155	-3.41	tyrosinase tyrosinase: common central domain protein
Ben3g12033	16	-3.41	hypothetical protein
Ben3g3378	2	-3.54	hypothetical protein
Ben3g3829	453	-3.54	hypothetical protein
Ben3g9597	596	-3.56	hypothetical protein
Ben3g10164	256	-3.58	hypothetical protein
Ben3g10576	3	-3.63	hypothetical protein
Ben3g8992	20	-3.73	hypothetical protein
Ben3g11040	71	-3.81	SGT1, suppressor of G2 allele of SKP1 protein, putative
Ben3g7892	133	-3.82	hypothetical protein
Ben3g4958	121	-3.86	subtilisin-like serine protease family protein
Ben3g997	14	-3.94	hypothetical protein
Ben3g4277	13	-4.18	O-methylsterigmatocystin oxidoreductase
Ben3g6862	13	-4.19	hypothetical protein
Ben3g9595	419	-4.54	hypothetical protein
Ben3g5035	15	-4.57	cryptochrome, DASH family protein
Ben3g8592	921	-7.85	hypothetical protein

hypothetical proteins
putative transcriptional regulators
involved in protein degradation
involved in cell wall degradation
lectin domain containing proteins
specifically mentioned proteins
nitrogen assimilation

bold SeqNames are in common 3dpi-Ben and 8dpi-Ben

Supplemental Table 10: List of genes differentially transcribed between 3 dpi and 8 dpi

SeqName	BaseMean	log2FoldChange	Description
Ben3g6862	24	-5.39	carbohydrate-binding module family 13 protein, putative
Ben3g6361	134	-5.34	nitrite reductase (NAD(P)H) large subunit
Ben3g6147	197	-5.32	ammonium transporter
Ben3g6359	170	-5.21	nitrate reductase (NADPH)
Ben3g6767	101	-4.86	probable urea active transporter 1
Ben3g6360	247	-4.73	putative high affinity nitrate transporter
Ben3g11893	35	-4.63	putative ribonuclease H-like protein
Ben3g4369	16	-4.52	putative amino-acid permease
Ben3g475	21	-4.05	oxalate decarboxylase
Ben3g11079	156	-4.03	cuticle-degrading protease
Ben3g9716	38	-3.90	isoflavone reductase-like protein
Ben3g7530	5	-3.84	cytochrome P450, family 51 (sterol 14-demethylase)
Ben3g9280	14	-3.83	extracellular elastinolytic metalloproteinase
Ben3g10121	3	-3.80	peptidase inhibitor i9
Ben3g4917	47	-3.70	Protein priA
Ben3g1845	37	-3.66	extracellular metalloprotease
Ben3g7775	11	-3.62	xanthine/uracil permease
Ben3g6155	13	-3.59	putative quinate permease
Ben3g5801	60	-3.59	MFS general substrate transporter
Ben3g10377	49	-3.53	chondroitin AC/alginate lyase
Ben3g10113	149	-3.50	pyridoxal-dependent decarboxylase domain protein
Ben3g4197	23	-3.48	putative pheromone-regulated membrane protein
Ben3g46	19	-3.45	hypothetical protein
Ben3g11080	14	-3.43	heterokaryon incompatibility protein
Ben3g4404	84	-3.33	chaperone protein DnaJ
Ben3g10114	47	-3.33	pyridoxal-dependent decarboxylase domain protein
Ben3g7999	79	-3.25	Protein rds1
Ben3g4357	7	-3.21	acetamidase/formamidase
Ben3g6863	72	-3.20	trehalose utilization protein
Ben3g2177	46	-3.17	glutathione-dependent formaldehyde-activating protein
Ben3g4046	268	-3.16	hypothetical protein
Ben3g10213	8	-3.16	helix loop helix DNA-binding domain protein
Ben3g4361	44	-3.16	APC amino acid permease
Ben3g10576	2	-3.15	hypothetical protein
Ben3g3282	7	-3.11	high-affinity nicotinic acid transporter
Ben3g8592	24	-3.09	hypothetical protein
Ben3g362	80	-3.06	glutathione transferase, putative
Ben3g3436	80	-3.06	MFS sugar transporter
Ben3g5460	3	-3.05	oxalate decarboxylase
Ben3g7084	20	-3.04	iron permease FTR1
Ben3g7643	6	-3.01	putative aspartyl protease
Ben3g2881	17	-2.98	zinc type alcohol dehydrogenase, putative
Ben3g9270	12	-2.94	aldo/keto reductase family containing protein
Ben3g2324	42	-2.93	carbohydrate-binding domain protein, putative
Ben3g5901	22	-2.93	hypothetical protein
Ben3g11328	9	-2.92	ubiquitin C
Ben3g3391	4	-2.90	m6a methyltransferase
Ben3g3816	14	-2.90	phosphoglycerate mutase family protein
Ben3g6269	33	-2.90	Guanyl-specific ribonuclease F1
Ben3g7993	58	-2.86	chondroitin AC/alginate lyase
Ben3g8626	21	-2.86	putative malate dehydrogenase
Ben3g2142	267	-2.84	endoplasmic reticulum protein
Ben3g6088	26	-2.82	serine carboxypeptidase
Ben3g5072	74	-2.81	glycoside hydrolase family 61 protein
Ben3g1402	384	-2.80	hypothetical protein
Ben3g9597	235	-2.79	fasciclin domain protein
Ben3g3426	5	-2.77	tyrosinase tyrosinase: common central domain protein
Ben3g11169	3	-2.74	SNF2 family amino-terminal protein
Ben3g6450	8	-2.73	DNA ligase 4

SeqName	BaseMean	log2FoldChange	Description
Ben3g6962	84	6.31	putative 1,4-beta-D-glucan cellobiohydrolase B
Ben3g8963	62	6.20	flavin containing amine oxidase
Ben3g28	37	6.08	pectate lyase
Ben3g5658	11	5.71	glycoside hydrolase family 61 protein
Ben3g8505	11	5.25	glycoside hydrolase family 61 protein
Ben3g8198	20	5.19	glycoside hydrolase family 61 protein
Ben3g7512	22	5.03	glycoside hydrolase family 5 protein
Ben3g6555	9	5.02	GDSL-like lipase/acylhydrolase
Ben3g860	5	5.02	glycoside hydrolase family 61 protein
Ben3g4089	6	4.92	glycoside hydrolase family 61 protein
Ben3g11844	34	4.89	hypothetical protein
Ben3g11686	7	4.86	endo-1,4-beta-xylanase, partial
Ben3g7261	8	4.85	putative pectate lyase
Ben3g8832	6	4.75	hypothetical protein
Ben3g10605	3	4.64	GDSL-like lipase/acylhydrolase
Ben3g10825	95	4.61	transmembrane protein, putative
Ben3g3368	3	4.58	pectate lyase, putative
Ben3g5383	4	4.57	glycoside hydrolase family 61 protein
Ben3g5878	52	4.52	hypothetical protein
Ben3g2669	3	4.51	CsbD domain protein
Ben3g5028	14	4.50	pectin lyase
Ben3g7787	14	4.50	chitin-binding domain protein
Ben3g797	42	4.41	cellobiohydrolase II
Ben3g7723	79	4.40	hypothetical protein
Ben3g7821	15	4.36	glycoside hydrolase family 61 protein
Ben3g5936	12	4.36	glycoside hydrolase family 18 protein
Ben3g11336	6	4.35	hypothetical protein
Ben3g9980	35	4.34	hexose transporter
Ben3g4824	27	4.31	1,4-beta-D-glucan cellobiohydrolase
Ben3g5824	27	4.29	hypothetical protein
Ben3g1689	11	4.29	pectate lyase
Ben3g3031	12	4.26	cell wall glycosyl hydrolase, putative
Ben3g4785	3	4.25	lipase
Ben3g6325	37	4.21	1,4-beta-D-glucan cellobiohydrolase B, putative
Ben3g9016	7	4.21	glycoside hydrolase family 61 protein
Ben3g4093	24	4.20	phthalate transporter
Ben3g4786	8	4.20	Lipase
Ben3g5054	28	4.19	mannan endo-1,4-beta-mannosidase
Ben3g1311	3	4.15	arabinan endo-1,5-alpha-L-arabinosidase
Ben3g7242	3	4.14	short chain dehydrogenase
Ben3g2852	2	4.12	putative cutinase Mb2006c
Ben3g7552	38	4.11	glycoside hydrolase family 1 protein
Ben3g10275	13	4.09	Tc5 transposase DNA-binding domain protein
Ben3g9025	4	4.08	endoglucanase II
Ben3g7235	123	4.06	short chain dehydrogenase/reductase, putative
Ben3g11071	5	4.01	hypothetical protein
Ben3g29	49	4.01	pectate lyase
Ben3g4251	67	3.99	glycoside hydrolase family 16 protein
Ben3g5299	2	3.97	glycoside hydrolase family 61 protein
Ben3g5104	8	3.95	glycoside hydrolase family 61 protein
Ben3g10680	32	3.94	hypothetical protein
Ben3g1717	4	3.94	lactonase, 7-bladed beta-propeller
Ben3g8935	6	3.91	Putative hydrolase Mb2247c
Ben3g11325	4	3.88	putative 30S ribosomal protein
Ben3g7942	2	3.84	pyranose 2-oxidase
Ben3g3500	3	3.83	pectate lyase, putative
Ben3g2499	28	3.81	hypothetical protein
Ben3g7384	22	3.80	GDSL-like lipase/acylhydrolase domain protein
Ben3g11324	10	3.79	putative 30S ribosomal protein

Ben3g710	75	-2.72	peptidyl-Lys metalloendopeptidase	*Ben3g6493*	5	3.78	hypothetical protein
Ben3g11549	9	-2.72	hypothetical protein	*Ben3g1944*	60	3.76	CsbD domain protein
Ben3g12503	6	-2.71	DUF3294 family protein	*Ben3g732*	7	3.75	hypothetical protein
Ben3g4201	17	-2.69	hypothetical protein	*Ben3g1349*	9	3.73	Pectinesterase OS=Aspergillus aculeatus GN=pme1 PE=2 SV=1
Ben3g8452	179	-2.68	lipoic acid synthetase	*Ben3g1770*	2	3.70	carbonate dehydratase
Ben3g5102	7	-2.67	glycoside hydrolase family 61 protein	*Ben3g8173*	5	3.70	chitin-binding domain protein
Ben3g7136	6	-2.67	rRNA (adenine-N6,N6)-dimethyltransferase	*Ben3g11590*	21	3.69	putative 30S ribosomal protein
Ben3g8036	28	-2.64	putative malate dehydrogenase	*Ben3g6074*	8	3.68	polysaccharide lyase family 4 protein
Ben3g5103	20	-2.62	3-ketoacyl-(acyl-carrier) reductase	*Ben3g10100*	10	3.66	endonuclease/exonuclease/phosphatase family protein
Ben3g12367	1	-2.59	cold response protein 1	*Ben3g5145*	21	3.65	transmembrane protein, putative
Ben3g10908	6	-2.59	putative transmembrane protein	*Ben3g4441*	2	3.65	glycoside hydrolase family 5 protein
Ben3g12534	1	-2.57	1,4-alpha-glucan branching enzyme	*Ben3g9185*	3	3.65	glycoside hydrolase family 61 protein
Ben3g11214	1	-2.57	hypothetical protein	*Ben3g4822*	38	3.65	putative glucan 1,3-beta-glucosidase D
Ben3g10581	144	-2.55	tyrosinase tyrosinase: common central domain protein	*Ben3g6571*	11	3.62	hypothetical protein
Ben3g4967	8	-2.53	cryptochrome, DASH family protein	*Ben3g8367*	100	3.60	hypothetical protein
Ben3g5646	209	-2.53	macrofage activating glycoprotein, putative	*Ben3g8947*	24	3.60	carbohydrate esterase family 4 protein
Ben3g2879	50	-2.53	MFS general substrate transporter	*Ben3g3570*	13	3.57	mannosyltransferase domain protein
Ben3g2772	54	-2.53	hypothetical protein	*Ben3g9437*	2	3.57	polysaccharide lyase family 4 protein
Ben3g1127	260	-2.53	trehalose 6-phosphate phosphatase	*Ben3g8490*	3	3.55	glycoside hydrolase family 61 protein
Ben3g11709	11	-2.53	alkaline phosphatase-like protein	*Ben3g12332*	1	3.55	endo-1,4-beta-xylanase
Ben3g4934	65	-2.51	dienelactone hydrolase family protein	*Ben3g12114*	3	3.55	hAT family dimerization protein
Ben3g4370	32	-2.50	ammonium transporter	*Ben3g7327*	209	3.55	PXB domain protein
Ben3g3283	12	-2.49	High-affinity nicotinic acid transporter	*Ben3g532*	42	3.53	hypothetical protein
Ben3g8469	8	-2.49	transmembrane protein, putative	*Ben3g4183*	9	3.52	pectate lyase
Ben3g5790	15	-2.47	cytochrome P450 family protein	*Ben3g10805*	17	3.50	hypothetical protein
Ben3g7357	7	-2.47	hypothetical protein	*Ben3g2421*	6	3.49	cellulose-binding GDSL lipase/acylhydrolase-like protein, putative
Ben3g1497	7	-2.46	apoptosis-inducing factor 2	*Ben3g9438*	10	3.48	rhamnogalacturonate lyase
Ben3g2854	77	-2.46	diphthine synthase	*Ben3g8520*	6	3.47	carboxylesterase
Ben3g9769	15	-2.45	putative 6-hydroxy-D-nicotine oxidase	*Ben3g12353*	24	3.46	hypothetical protein
Ben3g1018	2	-2.44	hypothetical protein	*Ben3g12450*	6	3.46	glycoside hydrolase family 3 protein
Ben3g4920	3	-2.44	oxalate decarboxylase	*Ben3g5920*	14	3.45	C4-dicarboxylate transporter/malic acid transporter
Ben3g8237	24	-2.41	phosphatidylcholine-hydrolyzing phospholipase C	*Ben3g1457*	3	3.45	family 16 endo-1,3(4)-beta-glucanase from glycoside hydrolase, putative
Ben3g406	222	-2.40	hypothetical protein	*Ben3g12053*	8	3.44	endo-1,4-beta-xylanase
Ben3g2501	3	-2.38	haloacid dehalogenase-like hydrolase	*Ben3g10539*	5	3.42	hypothetical protein
Ben3g8329	4	-2.38	bestrophin protein	*Ben3g2522*	2	3.42	40S ribosomal protein S14, putative
Ben3g4422	11	-2.36	NAD(P)H-binding family protein	*Ben3g12473*	2	3.42	pectate lyase, putative
Ben3g5306	22	-2.36	NAD-dependent histone deacetylase SIR2	*Ben3g4043*	34	3.41	MFS lactose permease, putative
Ben3g2705	23	-2.34	membrane protein, putative	*Ben3g10238*	2	3.40	hypothetical protein
Ben3g8600	1	-2.31	hypothetical protein	*Ben3g7706*	7	3.38	tetrapyrrole methylase
Ben3g251	235	-2.30	aromatic peroxygenase	*Ben3g4757*	157	3.38	glycoside hydrolase family 16 protein
Ben3g8185	3	-2.30	transmembrane protein, putative	*Ben3g1925*	10	3.37	arginase family protein
Ben3g1654	130	-2.30	transmembrane protein, putative	*Ben3g5857*	58	3.28	minor extracellular protease vpr protein
Ben3g2070	222	-2.29	extracellular metalloprotease	*Ben3g3004*	30	3.27	potassium/sodium efflux P-type ATPase
Ben3g2638	106	-2.29	sugar porter (SP) family MFS transporter	*Ben3g12138*	4	3.26	hypothetical protein
Ben3g8377	11	-2.29	ICE-like protease (caspase) p20 domain protein	*Ben3g10800*	10	3.26	hypothetical protein
Ben3g5355	1	-2.29	hypothetical protein	*Ben3g7482*	11	3.25	extracellular metalloprotease
Ben3g6826	1	-2.28	acetyltransferase (GNAT) family containing protein	*Ben3g8796*	5	3.25	rhamnogalacturonate lyase
Ben3g10210	58	-2.28	interferon-induced 6-16 family protein	*Ben3g8833*	2	3.23	glycoside hydrolase family 61 protein
Ben3g6724	6	-2.28	3-ketoacyl-(acyl-carrier) reductase	*Ben3g6875*	33	3.17	putative 1,4-beta-D-glucan cellobiohydrolase B
Ben3g302	1	-2.27	hypothetical protein	*Ben3g3232*	1	3.14	glycoside hydrolase family 61 protein
Ben3g4104	1	-2.27	S1/P1 nuclease	*Ben3g3188*	15	3.14	glycoside hydrolase family 10 protein
Ben3g4921	8	-2.26	S-adenosyl-L-methionine-dependent methyltransferase	*Ben3g5065*	14	3.14	pectate lyase
Ben3g908	10	-2.26	Probable peptide/nitrate transporter	*Ben3g11917*	7	3.10	pathogenesis-related protein PR5K (thaumatin family)
Ben3g8865	2	-2.25	cytochrome P450 family protein	*Ben3g4437*	2	3.09	glycoside hydrolase family 5 protein
Ben3g9114	18	-2.24	Pantothenate transporter liz1	*Ben3g9790*	1	3.09	ATP-dependent Clp protease ATP-binding subunit ClpX
Ben3g1597	2	-2.24	bacterial leucyl aminopeptidase	*Ben3g2536*	5	3.06	glycoside hydrolase catalytic core protein
Ben3g8948	408	-2.24	protein arginine N-methyltransferase 3	*Ben3g7030*	16	3.05	hemerythrin HHE cation-binding domain protein
Ben3g5495	1	-2.21	hypothetical protein	*Ben3g9296*	1	3.05	bestrophin protein
Ben3g7872	6	-2.20	Asp domain protein, putative	*Ben3g12499*	14	3.04	exopolygalacturonase, putative
Ben3g10853	7	-2.19	Mitochondrial dicarboxylate transporter	*Ben3g4874*	1	3.04	adenylosuccinate lyase
Ben3g4643	68	-2.18	serine protease	*Ben3g3019*	9	3.03	cutinase
Ben3g1179	7	-2.17	ICE-like protease (caspase) p20 domain protein	*Ben3g31*	53	3.02	pectate lyase

Ben3g9164	5	-2.17	hypothetical protein
Ben3g2143	22	-2.17	permease cytosine/purine uracil thiamine allantoin
Ben3g8596	57	-2.16	extracellular elastinolytic metalloproteinase
Ben3g997	2	-2.16	terpene synthase family, metal-binding domain protein
Ben3g7498	1	-2.15	putative alcohol dehydrogenase
Ben3g5035	2	-2.15	malate dehydrogenase (oxaloacetate-decarboxylating)
Ben3g10027	4	-2.15	peptidase family S41 domain protein
Ben3g6625	22	-2.14	putative secreted protein
Ben3g1698	5	-2.14	putative serine-rich protein C13G6,10c
Ben3g3059	10	-2.13	high-affinity nicotinic acid transporter
Ben3g8595	51	-2.13	hypothetical protein
Ben3g6277	24	-2.13	putative aryl alcohol dehydrogenase
Ben3g11468	42	-2.13	transmembrane protein, putative
Ben3g7851	63	-2.12	hypothetical protein
Ben3g5782	49	-2.12	transmembrane protein, putative
Ben3g5993	1	-2.12	STE-like pheromone receptor
Ben3g8612	3	-2.12	hypothetical protein
Ben3g2713	32	-2.10	hypothetical protein
Ben3g7232	16	-2.10	hypothetical protein
Ben3g2992	32	-2.10	galactose-proton symport protein, putative
Ben3g5566	3	-2.09	Uncharacterized protein
Ben3g4747	35	-2.09	BTB domain protein
Ben3g10077	10	-2.09	formate/nitrite transporter family protein
Ben3g7854	1	-2.09	hypothetical protein
Ben3g5152	13	-2.09	subtilisin-like serine protease family protein
Ben3g10094	1	-2.08	hypothetical protein
Ben3g7652	0	-2.07	DNA replication regulator SLD3
Ben3g5594	1	-2.07	beta-aspartyl-peptidase (threonine type)
Ben3g9967	11	-2.06	hypothetical protein
Ben3g8907	14	-2.06	proline dehydrogenase
Ben3g4536	7	-2.06	putative acetyl-CoA synthetase-like protein
Ben3g12264	1	-2.05	hAT family dimerization protein
Ben3g2958	5	-2.05	neutral/alkaline nonlysosomal ceramidase
Ben3g11519	2	-2.05	hypothetical protein
Ben3g3299	0	-2.05	double-stranded RNA-binding motif protein
Ben3g8673	260	-2.05	genomic scaffold, msy-sf-12 protein
Ben3g9775	3	-2.05	drug:H+ antiporter
Ben3g5320	36	-2.04	putative transmembrane protein
Ben3g9474	9	-2.04	transmembrane protein, putative
Ben3g5434	20	-2.03	hypothetical protein
Ben3g3886	9	-2.03	carboxylic acid transporter
Ben3g5369	1	-2.02	secreted protein, putative
Ben3g8739	1	-2.02	meiotic recombination protein DMC1, related protein
Ben3g2135	22	-2.02	chitin deacetylase
Ben3g862	4	-2.01	peptidyl-prolyl cis-trans isomerase
Ben3g9054	20	-2.01	putative acetyl-CoA synthetase-like protein
Ben3g1699	0	-2.01	putative formate/nitrite transporter family protein
Ben3g5783	46	-2.01	alpha-mannosyltransferase, putative
Ben3g2447	13	-2.00	hypothetical protein
Ben3g7876	2	-2.00	SMP-30/gluconolaconase/LRE-like region protein
Ben3g776	1	-2.00	rare lipoprotein A-like double-psi beta-barrel protein
Ben3g10780	16	-2.00	putative transmembrane protein

hypothetical proteins
putative transcriptional regulators
involved in protein degradation
involved in cell wall degradation
lectin domain containing proteins
specifically mentioned proteins
nitrogen assimilation

Ben3g7064	7	3.02	C4-dicarboxylate transporter/malic acid transporter
Ben3g9271	13	2.99	glycoside hydrolase family 6 protein
Ben3g8474	4	2.99	hypothetical protein
Ben3g10239	2	2.98	tyrosinase tyrosinase: common central domain protein
Ben3g6494	11	2.98	putative 30S ribosomal protein
Ben3g2619	8	2.98	peroxidase, putative
Ben3g5764	11	2.98	permease/ATP-binding ABC transporter
Ben3g9137	1	2.97	hypothetical protein
Ben3g12567	1	2.97	tyrosine kinase family catalytic domain protein
Ben3g5302	3	2.96	pectin lyase F, putative
Ben3g3501	6	2.96	pectate lyase, putative
Ben3g2563	98	2.95	cytochrome P450 family 6 protein
Ben3g7949	1	2.95	hypothetical protein
Ben3g8602	158	2.94	deuterolysin M35 metalloprotease
Ben3g8344	13	2.93	subtilisin-like serine protease family protein
Ben3g6509	1	2.92	hypothetical protein
Ben3g9730	2	2.91	endo-1,4-beta-xylanase
Ben3g7031	55	2.90	hemerythrin HHE cation-binding domain protein
Ben3g5935	27	2.90	papain family cysteine protease domain protein
Ben3g3461	86	2.90	ML domain protein
Ben3g2670	30	2.89	CsbD domain protein
Ben3g4134	21	2.89	endo-1,4-beta-xylanase
Ben3g578	29	2.89	PHB depolymerase family esterase
Ben3g6353	53	2.88	endo-polygalacturonase PG1
Ben3g5245	28	2.88	FAD-linked oxidoreductase
Ben3g4220	6	2.87	tyrosinase tyrosinase: common central domain protein
Ben3g12440	3	2.87	hypothetical protein
Ben3g11562	7	2.87	hypothetical protein
Ben3g8286	1	2.87	Flagellar attachment zone protein 1
Ben3g2518	1	2.85	putative 30S ribosomal protein
Ben3g5394	3	2.85	hypothetical protein
Ben3g7637	19	2.83	glycoside hydrolase family 10 protein
Ben3g4302	18	2.82	hypothetical protein
Ben3g10611	12	2.81	hypothetical protein
Ben3g1301	105	2.81	transmembrane protein, putative
Ben3g12507	1	2.81	DEAD/DEAH-box helicase
Ben3g8644	7	2.80	hypothetical protein
Ben3g5899	284	2.79	cyclin protein
Ben3g11689	22	2.79	glycoside hydrolase family 13 protein
Ben3g4407	94	2.78	DUF3602 family protein
Ben3g2363	48	2.78	putative fungal zn(2)-cys(6) binuclear cluster domain protein
Ben3g8513	70	2.75	putative 30S ribosomal protein
Ben3g7354	3	2.75	putative 30S ribosomal protein
Ben3g10797	10	2.75	hypothetical protein
Ben3g7065	7	2.74	C4-dicarboxylate transporter/malic acid transporter
Ben3g5454	31	2.74	xyloglucan-specific endo-beta-1,4-glucanase A
Ben3g1182	114	2.74	ABC transporter related
Ben3g2930	25	2.73	hypothetical protein
Ben3g10021	1	2.73	reverse transcriptase
Ben3g6012	31	2.73	homeobox domain protein
Ben3g7247	1	2.73	putative transmembrane protein
Ben3g6060	14	2.72	mannan endo-1,4-beta-mannosidase
Ben3g6771	5	2.72	putative mannan endo-1,4-beta-mannosidase
Ben3g6916	2	2.72	hypothetical protein
Ben3g4178	26	2.72	hypothetical protein
Ben3g760	2	2.71	related to ASD-1 rhamnogalacturonase B precursor
Ben3g4854	14	2.71	glycoside hydrolase family 5 protein
Ben3g8650	40	2.70	glycoside hydrolase family 61 protein, putative
Ben3g9797	1	2.70	putative serine-rich protein C13G6,10c
Ben3g6761	25	2.70	putative ribonuclease E inhibitor RraA
Ben3g6488	15	2.70	putative 30S ribosomal protein
Ben3g12400	5	2.69	hypothetical protein

Ben3g71	11	2.69	hypothetical protein
Ben3g12196	2	2.68	hAT family dimerization protein
Ben3g5441	9	2.68	glycoside hydrolase family 27 protein
Ben3g1580	31	2.68	cytochrome P450 family protein
Ben3g8746	41	2.68	putative glycoside hydrolase family 43 protein
Ben3g6896	1	2.66	tyrosinase tyrosinase: common central domain protein
Ben3g10948	1	2.66	hAT family dimerization protein
Ben3g2597	93	2.66	putative genomic scaffold, msy-sf-3 protein
Ben3g4240	6	2.65	endo-polygalacturonase PG1
Ben3g8640	2	2.64	hypothetical protein
Ben3g9439	25	2.64	putative hydrolase
Ben3g2773	4	2.63	homoaconitate hydratase
Ben3g4175	6	2.63	ICE-like protease (caspase) p20 domain protein
Ben3g1414	1	2.63	hypothetical protein
Ben3g5597	30	2.63	F-box-like domain protein
Ben3g1694	168	2.63	hypothetical protein
Ben3g10363	1	2.62	hypothetical protein
Ben3g5512	34	2.62	protocatechuate 3,4-dioxygenase beta subunit, putative
Ben3g8318	2	2.62	GNAT family acetyltransferase
Ben3g4472	12	2.61	FAD-binding domain protein
Ben3g1309	14	2.60	arabinan endo-1,5-alpha-L-arabinosidase
Ben3g8849	8	2.60	hypothetical protein
Ben3g10804	5	2.60	hypothetical protein
Ben3g3256	53	2.59	cyclin domain protein
Ben3g11921	40	2.59	hypothetical protein
Ben3g7748	1	2.59	3-ketoacyl-(acyl-carrier) reductase
Ben3g3441	4	2.59	hypothetical protein
Ben3g6355	122	2.59	alcohol oxidase
Ben3g4937	14	2.59	proteophosphoglycan protein, putative
Ben3g8294	19	2.58	macrofage activating glycoprotein, putative
Ben3g3125	12	2.57	GNAT family acetyltransferase
Ben3g7243	12	2.57	hypothetical protein
Ben3g4523	6	2.56	hypothetical protein
Ben3g10244	5	2.55	cys/met metabolism PLP-dependent enzyme
Ben3g7664	7	2.54	mucoidy inhibitor A
Ben3g10835	4	2.54	hypothetical protein
Ben3g4865	2	2.53	pectate lyase, putative
Ben3g11544	12	2.53	PII uridylyl-transferase
Ben3g3080	4	2.52	chitin deacetylase
Ben3g7754	76	2.52	peptidyl-prolyl cis-trans isomerase
Ben3g6067	36	2.52	alcohol oxidase
Ben3g3126	148	2.51	glutamyl-tRNA(Gln) amidotransferase subunit A
Ben3g8351	60	2.51	hypothetical protein
Ben3g9443	1	2.50	kinesin light chain
Ben3g2012	14	2.49	cellobiose dehydrogenase
Ben3g2199	1	2.49	DUF537-domain-containing protein
Ben3g7857	1	2.49	streptomycin biosynthesis protein StrI
Ben3g12281	1	2.49	hypothetical protein
Ben3g7353	1	2.49	30S ribosomal S17P-like protein, putative
Ben3g4236	7	2.48	hypothetical protein
Ben3g3590	0	2.48	cutinase
Ben3g3053	3	2.48	tRNA-splicing ligase RtcB
Ben3g1421	10	2.48	hypothetical protein
Ben3g7263	1	2.48	putative pectate lyase
Ben3g2768	23	2.48	transmembrane protein, putative
Ben3g4542	2	2.47	putative glutaminase GtaA
Ben3g3553	7	2.46	short-chain dehydrogenase/reductase family protein
Ben3g5563	1	2.46	fruiting body protein Sc7
Ben3g1313	54	2.45	glycoside hydrolase family 16 protein
Ben3g1789	1	2.45	hypothetical protein
Ben3g6035	5	2.44	ICE-like protease (caspase) p20 domain protein
Ben3g342	75	2.43	carboxylic acid transporter

Ben3g2636	92	2.42	pleiotropic drug resistance ABC transporter
Ben3g6316	1	2.42	ribosomal RNA large subunit methyltransferase J
Ben3g2402	5	2.42	hypothetical protein
Ben3g6201	1	2.42	putative pectinesterase A
Ben3g4096	37	2.41	hexose transporter
Ben3g1979	91	2.40	hypothetical protein
Ben3g2314	7	2.39	hypothetical protein
Ben3g6667	2	2.39	hypothetical protein
Ben3g865	187	2.39	mannose-1-phosphate guanylyltransferase
Ben3g11300	2	2.39	hypothetical protein
Ben3g10865	1	2.39	kinase domain protein
Ben3g10951	56	2.39	LRR protein
Ben3g8180	7	2.39	CHAT domain protein
Ben3g5944	5	2.38	glycoside hydrolase family 35 protein
Ben3g11131	0	2.38	hypothetical protein
Ben3g2519	2	2.37	hypothetical protein
Ben3g3144	19	2.37	hypothetical protein
Ben3g2791	1	2.37	glycoside hydrolase family 13 protein
Ben3g7734	2	2.36	BTB domain protein, putative
Ben3g9376	9	2.36	putative aromatic di-alanine and TPR containing protein
Ben3g7364	36	2.36	glycoside hydrolase family 62 protein
Ben3g7751	3	2.36	hypothetical protein
Ben3g8750	1	2.35	hypothetical protein
Ben3g8630	28	2.35	pectate lyase
Ben3g4267	1	2.35	short-chain dehydrogenase
Ben3g3814	189	2.34	fungal specific transcription factor domain protein
Ben3g9219	22	2.33	DJ-1/PfpI family protein
Ben3g9223	4	2.33	DJ-1/PfpI family protein
Ben3g3607	23	2.32	G protein coupled glucose receptor regulating Gpa2 protein
Ben3g8139	142	2.32	zinc finger, C2H2 type protein
Ben3g10156	48	2.32	ABC family B (MDR/TAP),protein
Ben3g12036	11	2.32	hypothetical protein
Ben3g8330	53	2.31	amino-acid N-acetyltransferase
Ben3g1708	20	2.31	regulator of G protein signaling domain protein
Ben3g5510	4	2.31	hypothetical protein
Ben3g9380	8	2.31	hypothetical protein
Ben3g6022	27	2.30	hypothetical protein
Ben3g8814	9	2.30	MFS sugar transporter
Ben3g8788	2	2.30	hypothetical protein
Ben3g8879	33	2.29	MFS general substrate transporter
Ben3g7725	6	2.29	NAD-dependent aldehyde dehydrogenase family protein
Ben3g6759	75	2.29	DUF1768 domain protein
Ben3g12545	1	2.28	putative Ty3/Gypsy polyprotein/retrotransposon
Ben3g5635	5	2.28	GDSL-like lipase/acylhydrolase
Ben3g6406	2	2.28	O-methylsterigmatocystin oxidoreductase
Ben3g8512	1	2.28	30S ribosomal S17P-like protein, putative
Ben3g6742	189	2.28	UV excision repair protein RAD23
Ben3g9155	11	2.28	cystathionine beta-lyase
Ben3g6034	102	2.28	1-phosphatidylinositol-4,5-bisphosphate phosphodiesterase
Ben3g1215	8	2.27	hypothetical protein
Ben3g8813	74	2.27	2-nitropropane dioxygenase
Ben3g5317	13	2.27	putative ubiquitin family protein
Ben3g4076	33	2.27	hypothetical protein
Ben3g2610	10	2.27	Siderophore iron transporter 3
Ben3g3800	32	2.26	glycoside hydrolase family 18 protein
Ben3g10868	1	2.26	BTB domain-containing protein
Ben3g9247	4	2.25	putative 30S ribosomal protein
Ben3g11982	1	2.25	hypothetical protein
Ben3g9368	267	2.25	hypothetical protein
Ben3g1787	3	2.24	ER membrane (Pkr1)
Ben3g2184	25	2.24	putative MFS sugar transporter
Ben3g2490	15	2.24	putative transmembrane protein

Ben3g2533	0	2.24	hypothetical protein
Ben3g3367	4	2.23	cysteine-rich secretory family protein
Ben3g4528	2	2.23	glycoside hydrolase family 43 protein
Ben3g4033	4	2.23	alpha-ketoglutarate-dependent taurine dioxygenase
Ben3g4340	359	2.22	fungal zn(2)-cys(6) binuclear cluster domain protein
Ben3g3824	82	2.22	chitin deacetylase
Ben3g4804	1	2.21	hypothetical protein
Ben3g12060	10	2.21	hypothetical protein
Ben3g364	8	2.20	RNA exonuclease 4
Ben3g249	39	2.20	hypothetical protein
Ben3g9230	29	2.19	cytochrome P450 family monooxygenase
Ben3g101	13	2.19	glycoside hydrolase family 28 protein
Ben3g2599	19	2.19	hypothetical protein
Ben3g7470	145	2.19	hypothetical protein
Ben3g9061	14	2.19	putative ubiquitin-protein ligase
Ben3g12330	0	2.19	DDE family endonuclease
Ben3g6068	4	2.19	Alcohol oxidase
Ben3g7541	0	2.19	glycoside hydrolase family 61 protein
Ben3g4333	3	2.19	cytochrome b5-like heme/steroid-binding domain protein
Ben3g870	8	2.18	guanine nucleotide-binding protein alpha-4 subunit
Ben3g5022	30	2.18	peroxisomal targeting signal 1 receptor
Ben3g2085	7	2.18	glycoside hydrolase family 92 protein
Ben3g8707	6	2.18	glycoside hydrolase family 43 protein
Ben3g8846	15	2.18	hypothetical protein
Ben3g4610	20	2.17	zinc finger, ZZ type protein
Ben3g6500	4	2.17	putative 30S ribosomal protein
Ben3g6731	7	2.17	Plasma membrane proteolipid 3
Ben3g5160	71	2.16	serine protease
Ben3g8760	1	2.16	GDSL-like lipase/acylhydrolase
Ben3g919	46	2.16	Ecl1 domain protein, putative
Ben3g2698	65	2.16	peptidyl-lys metalloendopeptidase
Ben3g11502	3	2.15	hypothetical protein

Ben3g7379	5	2.15	endo-polygalacturonase PG1
Ben3g1910	34	2.15	phosphatase regulatory subunit, putative
Ben3g2325	1	2.14	DUF1992 domain protein
Ben3g3381	22	2.14	putative transmembrane protein
Ben3g7656	3	2.13	hypothetical protein
Ben3g6961	0	2.13	RNA recognition motif protein, putative
Ben3g11825	2	2.13	glycosyl hydrolase family 10 domain-containing protein
Ben3g2372	30	2.13	DSBA domain-containing protein
Ben3g7633	69	2.13	cytochrome P450 family 6 protein
Ben3g2506	2	2.13	hypothetical protein
Ben3g2570	9	2.12	glucose oxidase-like protein
Ben3g6981	15	2.12	von willebrand factor type A domain protein
Ben3g12384	19	2.12	anucleate primary sterigmata protein B, putative
Ben3g10943	75	2.11	hypothetical protein
Ben3g2062	31	2.11	cytochrome P450 family monooxygenase
Ben3g4809	7	2.10	Wbp11 domain protein, putative
Ben3g244	99	2.10	U-box protein
Ben3g12244	6	2.10	MFS transporter, putative
Ben3g2124	56	2.09	ADP-ribosylation factor-like protein 2
Ben3g661	2	2.09	putative lysine decarboxylase
Ben3g2741	5	2.08	hypothetical protein
Ben3g8352	260	2.08	anucleate primary sterigmata protein B, putative
Ben3g11904	3	2.08	hypothetical protein
Ben3g6835	16	2.08	mannoprotein
Ben3g606	58	2.07	hypothetical protein
Ben3g8899	3	2.07	aldose 1-epimerase
Ben3g8161	51	2.07	2-keto-gluconate dehydrogenase, putative
Ben3g4382	14	2.07	4-hydroxybenzoate polyprenyltransferase
Ben3g8313	6	2.07	hypothetical protein
Ben3g11826	1	2.07	short chain dehydrogenase/reductase family protein
Ben3g4823	46	2.07	P-loop nucleoside triphosphate hydrolase, putative
Ben3g1259	83	2.07	putative mucoidy inhibitor A
Ben3g6204	14	2.07	putative endo-beta-1,4-glucanase D
Ben3g1526	6	2.07	glycoside hydrolase family 3 protein
Ben3g699	126	2.06	UDP-galactose transporter Gms1
Ben3g12432	0	2.06	endo-1,4-beta-xylanase
Ben3g7843	23	2.06	glycoside hydrolase family 74 protein
Ben3g1704	110	2.06	band 7 family protein, putative
Ben3g1008	13	2.06	flavin containing monooxygenase/FMO family protein
Ben3g7770	0	2.05	hypothetical protein
Ben3g4273	154	2.05	acyl-CoA dehydrogenase
Ben3g5619	65	2.04	putative hydrolase
Ben3g225	5	2.04	methyltransferase domain protein
Ben3g2359	4	2.04	putative transmembrane protein
Ben3g3419	6	2.04	GMC oxidoreductase
Ben3g3851	375	2.03	chitin synthase
Ben3g10821	1	2.03	putative vegetative incompatibility protein HET-E-1
Ben3g10515	0	2.02	ATP-dependent helicase/deoxyribonuclease subunit B
Ben3g23	1	2.02	tigger transposable element-derived protein
Ben3g9325	4	2.02	carbohydrate esterase family 4 protein
Ben3g3684	2	2.02	copper radical oxidase
Ben3g7481	22	2.01	hypothetical protein
Ben3g307	32	2.01	oligosaccharyl transferase subunit OST3/OST6 family protein, putative
Ben3g1356	105	2.01	glycosyltransferase family 32 protein
Ben3g4752	14	2.01	fasciclin domain protein
Ben3g10044	1	2.01	glycosyltransferase family 8 protein
Ben3g2277	13	2.01	glycoside hydrolase family 9 protein
Ben3g10047	1	2.01	glycosyltransferase family 2 protein
Ben3g6127	10	2.01	alcohol dehydrogenase, catalytic domain, GroES family protein
Ben3g2800	1	2.01	hypothetical protein
Ben3g1115	9	2.00	glycosyl hydrolase family 10 protein

Supplemental Table 11: List of differentially transcribed genes coding for carbohydrate active enzymes

**early up (57)**	
**SeqName**	**CAZy Family**
Ben3g3051	AA1
Ben3g6820	AA3
Ben3g9957	AA3
Ben3g136	AA6
Ben3g9725	AA7
Ben3g9957	AA8
Ben3g4060	AA9
Ben3g4703	AA9
Ben3g8649	AA9
Ben3g8740	AA9
Ben3g1402	CBM48
Ben3g9325	CE4
Ben3g1351	CE8
Ben3g7881	CE8
Ben3g4929	GH0
Ben3g7552	GH1
Ben3g7766	GH1
Ben3g3189	GH10
Ben3g4134	GH10
Ben3g7637	GH10
Ben3g8715	GH12
Ben3g1402	GH13
Ben3g7110	GH13
Ben3g3800	GH18
Ben3g3800	GH18
Ben3g5936	GH18
Ben3g1278	GH25
Ben3g7836	GH27
Ben3g4243	GH28
Ben3g4739	GH28
Ben3g4759	GH28
Ben3g5030	GH28
Ben3g7379	GH28
Ben3g1691	GH3
Ben3g5851	GH3
Ben3g6898	GH3
Ben3g7870	GH3
Ben3g2183	GH31
Ben3g1421	GH43
Ben3g10264	GH45
Ben3g3119	GH51
Ben3g6417	GH53
Ben3g4759	GH55
Ben3g9279	GH6
Ben3g7364	GH62
Ben3g1127	GT20
Ben3g4088	GT3
Ben3g8525	GT8
Ben3g1256	PL1
Ben3g4052	PL1

**late up (136)**	
**SeqName**	**CAZy Family**
Ben3g3051	AA1
Ben3g2503	AA3
Ben3g6820	AA3
Ben3g9957	AA3
Ben3g2012	AA3
Ben3g9725	AA7
Ben3g9957	AA8
Ben3g2503	AA8
Ben3g2012	AA8
Ben3g5658	AA9
Ben3g8740	AA9
Ben3g7821	AA9
Ben3g6204	AA9
Ben3g9025	AA9
Ben3g4089	AA9
Ben3g4060	AA9
Ben3g8649	AA9
Ben3g8198	AA9
Ben3g860	AA9
Ben3g5383	AA9
Ben3g5299	AA9
Ben3g8833	AA9
Ben3g5104	AA9
Ben3g8490	AA9
Ben3g8505	AA9
Ben3g3232	AA9
Ben3g9185	AA9
Ben3g1402	CBM48
Ben3g578	CE1
Ben3g4700	CE12
Ben3g6555	CE12
Ben3g8760	CE12
Ben3g5635	CE12
Ben3g10605	CE3
Ben3g3824	CE4
Ben3g8563	CE4
Ben3g9325	CE4
Ben3g1351	CE8
Ben3g6201	CE8
Ben3g7881	CE8
Ben3g7766	GH1
Ben3g7552	GH1
Ben3g4134	GH10
Ben3g11825	GH10
Ben3g11686	GH10
Ben3g12053	GH10
Ben3g9730	GH10
Ben3g3189	GH10
Ben3g7637	GH10
Ben3g1469	GH105

**differential (97)**	
**SeqName**	**CAZy Family**
Ben3g2570	AA3
Ben3g3419	AA3
Ben3g6355	AA3
Ben3g6068	AA3
Ben3g2012	AA3
Ben3g2570	AA8
Ben3g2012	AA8
Ben3g5658	AA9
Ben3g7821	AA9
Ben3g9016	AA9
Ben3g6204	AA9
Ben3g9025	AA9
Ben3g4089	AA9
Ben3g8198	AA9
Ben3g860	AA9
Ben3g5383	AA9
Ben3g5299	AA9
Ben3g8833	AA9
Ben3g5104	AA9
Ben3g8490	AA9
Ben3g8505	AA9
Ben3g3232	AA9
Ben3g9185	AA9
Ben3g1115	CBM22
Ben3g3188	CBM22
Ben3g578	CE1
Ben3g6555	CE12
Ben3g8760	CE12
Ben3g5635	CE12
Ben3g2421	CE16
Ben3g10605	CE3
Ben3g8947	CE4
Ben3g3824	CE4
Ben3g9325	CE4
Ben3g1349	CE8
Ben3g6201	CE8
Ben3g7552	GH1
Ben3g1115	GH10
Ben3g4134	GH10
Ben3g6571	GH10
Ben3g3188	GH10
Ben3g11825	GH10
Ben3g11686	GH10
Ben3g12053	GH10
Ben3g9730	GH10
Ben3g7637	GH10
Ben3g11689	GH13
Ben3g1313	GH16
Ben3g4757	GH16
Ben3g3800	GH18

Ben3g7223	PL1
Ben3g8007	PL1
Ben3g3530	PL3
Ben3g4864	PL3
Ben3g4865	PL3
Ben3g4881	PL3
Ben3g5034	PL9

Ben3g8715	GH12
Ben3g1402	GH13
Ben3g11689	GH13
Ben3g4700	GH13
Ben3g6533	GH131
Ben3g1313	GH16
Ben3g7900	GH16
Ben3g1229	GH16
Ben3g3800	GH18
Ben3g5936	GH18
Ben3g1278	GH25
Ben3g3511	GH27
Ben3g7836	GH27
Ben3g4243	GH28
Ben3g6353	GH28
Ben3g4240	GH28
Ben3g4739	GH28
Ben3g5030	GH28
Ben3g7379	GH28
Ben3g4759	GH28
Ben3g7870	GH3
Ben3g1691	GH3
Ben3g533	GH3
Ben3g5851	GH3
Ben3g6897	GH3
Ben3g6898	GH3
Ben3g5859	GH3
Ben3g2183	GH31
Ben3g5944	GH35
Ben3g1421	GH43
Ben3g4528	GH43
Ben3g6162	GH43
Ben3g1309	GH43
Ben3g1311	GH43
Ben3g10263	GH45
Ben3g10264	GH45
Ben3g7510	GH5
Ben3g2825	GH5
Ben3g2824	GH5
Ben3g6060	GH5
Ben3g5054	GH5
Ben3g6771	GH5
Ben3g4441	GH5
Ben3g3119	GH51
Ben3g6417	GH53
Ben3g4759	GH55
Ben3g797	GH6
Ben3g9271	GH6
Ben3g7364	GH62
Ben3g6962	GH7
Ben3g4824	GH7
Ben3g6325	GH7
Ben3g7843	GH74
Ben3g2085	GH92

Ben3g5936	GH18
Ben3g5441	GH27
Ben3g6353	GH28
Ben3g4240	GH28
Ben3g7379	GH28
Ben3g1526	GH3
Ben3g5944	GH35
Ben3g1421	GH43
Ben3g4528	GH43
Ben3g1309	GH43
Ben3g1311	GH43
Ben3g7512	GH5
Ben3g4437	GH5
Ben3g4822	GH5
Ben3g6060	GH5
Ben3g5054	GH5
Ben3g6771	GH5
Ben3g4441	GH5
Ben3g797	GH6
Ben3g9271	GH6
Ben3g7364	GH62
Ben3g6962	GH7
Ben3g4824	GH7
Ben3g6325	GH7
Ben3g7843	GH74
Ben3g2085	GH92
Ben3g3851	GT2
Ben3g31	PL1
Ben3g29	PL1
Ben3g8630	PL1
Ben3g28	PL1
Ben3g1689	PL1
Ben3g5028	PL1
Ben3g4865	PL3
Ben3g3500	PL3
Ben3g4183	PL3
Ben3g7261	PL3
Ben3g7263	PL3
Ben3g12473	PL3
Ben3g3501	PL3
Ben3g3553	PL3
Ben3g8796	PL4
Ben3g6074	PL4
Ben3g760	PL4
Ben3g9438	PL4
Ben3g9437	PL4
Ben3g5065	PL9

Ben3g3107	GT17
Ben3g975	GT5
Ben3g8525	GT8
Ben3g31	PL1
Ben3g29	PL1
Ben3g8630	PL1
Ben3g28	PL1
Ben3g8007	PL1
Ben3g1689	PL1
Ben3g6491	PL1
Ben3g1256	PL1
Ben3g4052	PL1
Ben3g7223	PL1
Ben3g5028	PL1
Ben3g4865	PL3
Ben3g3500	PL3
Ben3g7261	PL3
Ben3g7263	PL3
Ben3g12473	PL3
Ben3g3530	PL3
Ben3g6723	PL3
Ben3g4864	PL3
Ben3g4881	PL3
Ben3g3501	PL3
Ben3g6074	PL4
Ben3g760	PL4
Ben3g10405	PL4
Ben3g9438	PL4
Ben3g9437	PL4
Ben3g5283	PL4
Ben3g5065	PL9
Ben3g5034	PL9
